# Supplementary material for: Neoadjuvant immunotherapy with nivolumab and ipilimumab induces major pathological responses in patients with head and neck squamous cell carcinoma
Source: Nat Commun. 2021 Dec 22;12:7348. doi: 10.1038/s41467-021-26472-9 (PMC8695578; doi:10.1038/s41467-021-26472-9)
Supplement: Supplementary file 1 — Supplementary Information [file 41467_2021_26472_MOESM1_ESM.pdf]

Supplementary information

Supplement to: “Neoadjuvant immunotherapy with nivolumab and ipilimumab induces major pathological responses in patients with head and neck squamous cell carcinoma”

**Contents**	**Page(s)**
Supplementary Table 1	2
Supplementary Table 2	3
Supplementary Table 3	4
Supplementary Table 4	5
Supplementary Table 5	6
Supplementary Table 6	7
Supplementary Figure 1	8
Supplementary Figure 2	9
Supplementary Figure 3	10
Supplementary Figure 4	11
Supplementary Figure 5	12, 13
Supplementary Figure 6	14, 15
Supplementary Figure 7	16
Supplementary Figure 8	17
Supplementary References	18
Supplementary Note 1: Trial Protocol	19–118

ID	Trial phase	Subsite	Tumor status	Previous treatments for same HNSCC	Salvaged	HPV	cTNM	cStage	Regimen	Primary tumor surgery	Neck dissection	Reconstruction	pTNM	pStage	Adjuvant therapy	MR-RECIST	Primary tumor response (%)	Best nodal response
**NPR**																		
IMC-2	Phase Ib	oropharynx	recurrence	Surgery + RT	yes	neg	rT2N0	Rec	NIVO MONO	Left hemiglossectomy with base of tongue resection	Ipsilateral level I-III	FRFF	rT2N0	Rec	none	SD	0	NA
IMC-6	Phase Ib	oral cavity	recurrence	Surgery	no	neg	rT4aN0	Rec	NIVO MONO	Left anterior floor of mouth resection with segmental mandibulectomy	Ipsilateral completive level IV-V	PM	rT4aN0	Rec	none	PD	0	NPR
IMC-7	Phase Ib	oropharynx	recurrence	Cetuximab-RT	yes	neg	rT3N0	Rec	NIVO MONO	Right hemiglossectomy with base of tongue resection and segmental mandibulectomy	Ipsilateral level I-IV	PM	rT2N2b	Rec	none	PD	0	NPR
IMC-9	Phase Ib	oral cavity	primary		no	neg	T3N2c	IVA	COMBO	Left hemiglossectomy with segmental mandibulectomy	Bilateral level I-III	FRFF	T3N3b	IVB	RT	SD	0	NPR
IMC-33	Phase IIa	oral cavity	primary		no	neg	T3N0	III	COMBO	Left hemiglossectomy	Ipsilateral level I-IV	FRFF	T3N3b	IVB	RT	NE	0	NPR
IMC-37	Phase IIa	oral cavity	primary		no	upp	T3N0	III	COMBO	Subtotal glossectomy	Bilateral level I-IV	ALT	T3N2b	IVA	RT	PD	0	NPR
IMC-38	Phase IIa	oral cavity	primary		no	neg	T3N0	III	COMBO	Subtotal glossectomy with anterior floor of mouth resection and segmental mandibulectomy	Bilateral level I-IV	FFF	T3N2b	IVA	RT	SD	0	NPR
IMC-3	Phase Ib	oral cavity	primary		no	neg	T4aN2b	IVA	NIVO MONO	Left anterior floor of mouth resection with segmental mandibulectomy	Ipsilateral level I-III	FRFF	T4aN2b	IVA	RT	SD	0	NPR
IMC-26	Phase IIa	oral cavity	primary		no	neg	T4aN2b	IVA	COMBO	Right hemimaxillectomy with orbital exenteration	Ipsilateral level I-III	ALT	T4aN2b	IVA	RT	PD	13	NPR
IMC-10	Phase Ib	oral cavity	primary		no	neg	T4aN2c	IVA	COMBO	Left anterior floor of mouth resection with segmental mandibulectomy	Bilateral level I-IV	FFF	T4aN2c	IVA	RT	SD	19	MPR
IMC-30	Phase IIa	oral cavity	recurrence	Surgery	no	neg	rT2N0	Rec	COMBO	Anterior floor of mouth resection with segmental mandibulectomy	Ipsilateral level I-III	FFF	rT4aN0	Rec	RT	PD	19	NA
IMC-13	Phase Ib	oral cavity	primary		no	neg	T2N0	II	COMBO	Left anterior floor of mouth resection with marginal mandibulectomy	Bilateral level I-III	ALT	T2N0	II	none	NE	21	NA
IMC-25	Phase IIa	oral cavity	primary		no	neg	T3N2a	IVA	COMBO	Right hemiglossectomy with base of tongue, anterior pharyngeal arch and soft palate resection with marginal mandibulectomy	Ipsilateral level I-IV	FRFF	T2N2b	IVA	RT	SD	22	MPR
IMC-23	Phase IIa	oral cavity	primary		no	neg	T4aN1	IVA	COMBO	Right anterior floor of mouth resection with segmental mandibulectomy	Ipsilateral level I-IV	FFF	T4aN2a	IVA	RT	SD	26	NPR
IMC-35	Phase IIa	oral cavity	primary		no	neg	T4aN0	IVA	COMBO	Right anterior floor of mouth resection with partial glossectomy and segmental mandibulectomy	Ipsilateral level I-III	FFF	T2N0	II	none	SD	27	NA
IMC-24	Phase IIa	oral cavity	recurrence	Cetuximab-RT	yes	neg	rT3N0	Rec	COMBO	Subtotal glossectomy	Bilateral level I-IV	PM	rT3N0	Rec	none	SD	28	NA
IMC-28	Phase IIa	oral cavity	primary		no	neg	T3N0	III	COMBO	Right anterior floor of mouth resection with marginal mandibulectomy	Ipsilateral level I-IV	FRFF	T3N1	III	none	NE	33	NPR
IMC-8	Phase Ib	oropharynx	recurrence	Cisplatin-RT	yes	neg	rT4aN0	Rec	NIVO MONO	Total glossectomy with right segmental mandibulectomy	Ipsilateral level I-V, contralateral level I	PM	rT2N0	Rec	none	SD	43	NA
**PPR**																		
IMC-11	Phase Ib	larynx	recurrence	RT	yes	neg	rT3N0	Rec	COMBO	Total laryngectomy	none	none	rT3N0	Rec	none	SD	63	NA
IMC-27	Phase IIa	oral cavity	primary		no	neg	T4aN2b	IVA	COMBO	Right anterior floor of mouth resection with segmental mandibulectomy	Bilateral level I-IV	DCIA	T4aN2b	IVA	RT	SD	69	NPR
**MPR**																		
IMC-22	Phase IIa	oropharynx	primary		no	pos	T4N1	III	COMBO	Right resection of base of tongue, soft palate and anterior pharyngeal arch	Ipsilateral level I-V	ALT	T2N1	I	RT	SD	90	PPR
IMC-17	Phase IIa	oral cavity	primary		no	neg	T4aN0	IVA	COMBO	Right hemimaxillectomy	Selective dissection of 1 lipsilateral evel 2 node	none	T1N0	I	RT	SD	92	NA
IMC-39	Phase IIa	oral cavity	primary		no	neg	T3N0	III	COMBO	Right hemiglossectomy and floor of mouth resection	Ipsilateral level I-IV	FRFF	T1N0	I	none	PR	92	NA
IMC-15	Phase Ib	oral cavity	primary		no	neg	T3N1	III	COMBO	Left hemiglossectomy with floor of mouth, base of tongue and anterior pharyngeal arch resection with segmental mandibulectomy	Ipsilateral level I-IV	FRFF	T2N2b	IVA	RT	SD	93	NPR
IMC-36	Phase IIa	oral cavity	recurrence	Surgery	no	neg	rT2N0	Rec	COMBO	Left buccal resection	Selective dissection of 1 lipsilateral evel 2 node	FRFF	rT1N0	Rec	RT	NE	94	NA
IMC-4	Phase Ib	oral cavity	primary		no	neg	T3N1	III	NIVO MONO	Right hemiglossectomy with base of tongue, soft palate and floor of mouth resection with marginal mandibulectomy	Ipsilateral level I-IV	FRFF	T1N2b	IVA	none	SD	95	MPR
IMC-12	Phase Ib	oral cavity	recurrence	Surgery	no	neg	rT4aN0	Rec	COMBO	Left hemiglossectomy with segmental mandibulectomy	Ipsilateral level I-V	PM	rT1N0	Rec	none	NE	95	NA
IMC-31	Phase IIa	oral cavity	primary		no	neg	T2N0	II	COMBO	Right hemiglossectomy	Ipsilateral level I-III	FRFF	T1N0	I	none	SD	99	NA
IMC-29	Phase IIa	oral cavity	primary		no	neg	T2N0	II	COMBO	Right hemiglossectomy	Ipsilateral level I-III	none	T0N1	III	none	PR	100	NPR
**NE**																		
IMC-21	Phase IIa	oral cavity	primary		no	neg	T3N1	III	COMBO	NA	NA	NA	NE	NE	NA	PR	NE	NE
IMC-32	Phase IIa	oral cavity	recurrence	Surgery + RT	yes	neg	rT3N1	Rec	COMBO	NA	NA	NA	NE	NE	NA	PD	NE	NE
IMC-34	Phase IIa	oral cavity	primary		no	neg	T3N3b	IVB	COMBO	NA	NA	NA	NE	NE	NA	NE	NE	NE

Supplementary Table 1 | Overview of individual clinical, imaging and pathological response data.
TNM and staging according to AJCC 8th edition. Rec, recurrent; NIVO, nivolumab; COMBO, nivolumab + ipilimumab; FRFF, free radial forearm flap; PM; pectoralis major flap; ALT, anterolateral thigh flap; FFF, free fibula flap; DCIA, deep circumflex iliac artery flap; NE, not evaluable; NA, not applicable; PD, progressive disease; SD, stable disease; PR, partial response; MPR, major pathological response; PPR, partial pathological response; NPR, no pathological response.

	NIVO MONO (*n* =6)		COMBO (*n* =26)		Total (*n* =32)	
**Adverse event**	**Any grade**	**Grade 3–4**	**Any grade**	**Grade 3–4**	**Any grade**	**Grade 3–4**
Any irAE, *n* (%)	4 (67)	2 (33)	18 (69)	10 (38)	22 (69)	12 (38)
ALT increased, *n* (%)	0	0	11 (42)	5 (19)	11 (34)	5 (16)
AST increased, *n* (%)	0	0	10 (38)	0	10 (31)	0
GGT increased, *n* (%)	0	0	9 (35)	4 (15)	9 (28)	4 (13)
Alkaline phosphatase increased, *n* (%)	0	0	8 (31)	1 (4)	8 (25)	1 (3)
Fatigue, *n* (%)	0	0	4 (15)	0	4 (13)	0
Thyroiditis, *n* (%)	0	0	4 (15)	1 (4)	4 (13)	1 (3)
Colitis, *n* (%)	1 (17)	1 (17)	2 (8)	2 (8)	3 (9)	3 (9)
Rash, *n* (%)	0	0	3 (12)	1 (4)	3 (9)	1 (3)
Sarcoid-like reaction, *n* (%)	0	0	3 (12)	0	3 (9)	0
Diarrhea, *n* (%)	1 (17)	0	1 (4)	0	2 (6)	0
Pruritus, *n* (%)	1 (17)	0	1 (4)	0	2 (6)	0
Pericarditis, *n* (%)	1 (17)	1 (17)	0	0	1 (3)	1 (3)
Lymphopenia, *n* (%)	0	0	1 (4)	1 (4)	1 (3)	1 (3)
Allergic reaction, *n* (%)	0	0	1 (4)	0	1 (3)	0
Bilirubin increased, *n* (%)	1 (17)	0	0	0	1 (3)	0
Dry mouth, *n* (%)	0	0	1 (4)	0	1 (3)	0
Eosinophilia, *n* (%)	0	0	1 (4)	0	1 (3)	0
Gastritis, *n* (%)	0	0	1 (4)	0	1 (3)	0
Hepatitis, *n* (%)	0	0	1 (4)	0	1 (3)	0
Hyperthyroidism, *n* (%)	0	0	1 (4)	0	1 (3)	0
Hypothyroidism, *n* (%)	0	0	1 (4)	0	1 (3)	0

Supplementary Table 2 | Adverse events at least possibly related to neoadjuvant treatment according to CTCAE v.4,03, per neoadjuvant treatment and for the whole trial population.

CTCAE, common terminology criteria for adverse events; irAE, immune-related adverse event;
ALT, alanine aminotransferase; AST, aspartate aminotransferase; GGT, gamma-glutamyl transferase.

**Characteristic**	**NIVO MONO (*n* =6)**	**COMBO (*n* =23)**	**Total (*n* =29)**
Median length of hospitalization, days (IQR)	17 (5)	16 (16)	16 (12)
60-day postoperative complications*, *n* of patients (%)			
0	0	2 (9)	2 (7)
I	0	1 (4)	1 (3)
II	4 (67)	13 (57)	17 (59)
III-a	0	3 (13)	3 (10)
III-b	2 (33)	3 (13)	5 (17)
IV	0	1 (4)	1 (3)
Frequent postoperative complications**, *n* of patients with complication (%)			
Wound infection requiring antibiotics	2 (33)	12 (52)	14 (48)
Pneumonia requiring antibiotics	2 (33)	6 (26)	8 (28)
Anemia requiring transfusion	0	5 (22)	5 (17)
Oral fetor requiring antibiotics	0	4 (17)	4 (14)
Unsafe oral intake requiring gastrostomy tube placement	0	4 (17)	4 (14)
Hypertension requiring treatment	1 (17)	2 (9)	3 (10)
Elektrolyte disbalance requiring treatment	1 (17)	2 (9)	3 (10)
Reconstruction flap failure requiring new flap reconstruction	0	3 (13)	3 (10)
60-day surgical reinterventions, *n* of surgeries			
Nectrotectomy + new flap reconstruction	0	4	4
Necrotectomy	3	0	3
Postoperative hemorrhage coagulation	1	1	2
Cervical exploration	0	2	2
30-day readmission, *n* of patients (%)	2 (33)	6 (26)	8 (28)
Margin status, *n* of patients (%)			
Negative	6 (100)	22 (96)	28 (97)
Positive	0	1 (4)	1 (3)

Supplementary Table 3 | Surgical specifications and post-surgical complications according to Clavien-Dindo, per neoadjuvant treatment, and for the whole trial population.

Percentages may not add up to 100 because of rounding.

* Patients classified according to highest-grade complication. **Grade ≥ 2, occurring in at least 3 patients. IQR, interquartile range.

	All patients treated with PORT (n=15)	
**Adverse event**	**Any grade, *n (%)***	**Grade 3-4, *n (%)***
Any	15 (100)	9 (60)
Mucositis	13 (87)	3 (20)
Dermatitis	10 (67)	2 (13)
Dysphagia	8 (53)	5 (33)
Dry mouth	5 (33)	0
Localized edema	4 (27)	0
Dysgeusia	2 (13)	0
Localized pain	1 (7)	0
Radionecrosis of mandible	1 (7)	1 (7)

Supplementary Table 4 | Postoperative radiotherapy-associated adverse events according to CTCAE v.4.03.

CTCAE, common terminology criteria for adverse events; PORT, post-operative radiotherapy.

Characteristic	Historical cohort (N=114)
Median age, years (range)	62 (23-92)
Sex, *N (%)*	
Male	67 (59)
Female	47 (41)
Tumor site, *N (%)*	
Oral cavity	99 (87)
Oropharynx	15 (13)
HPV status, *N* of the 15 oropharyngeal tumors	
Positive	2
Negative	9
Unknown	4
HNSCC status, *N (%)*	
Primary	92 (81)
Recurrent	22 (19)
Clinical T-stage, *N (%)*	
T2	38 (33)
T3	36 (32)
T4	40 (35)
Clinical N-stage, *N (%)*	
N0	60 (53)
N1	12 (11)
N2	25 (22)
N3	17 (15)
Clinical disease stage (AJCC 7th ed), *N (%)*	
II	18 (16)
III	16 (14)
IV	58 (51)
Recurrent	22 (19)
Received adjuvant (chemo) radiotherapy	
Yes	60 (53)
No	54 (47)

Supplementary Table 5 | Characteristics of patients in the historical HNSCC cohort.
The historical cohort consisted of 114 HNSCC patients treated with extensive (salvage) surgery (with or without post-operative (chemo)radiotherapy) between 2013 and 2017 at our hospital, without neoadjuvant treatment. HNSCC, head and neck squamous cell carcinoma.

	MPR	PPR or NPR	Total
**PR or CR**	2	0	2
**SD or PD**	5	17	22
**Total**	7	17	
	**Sensitivity**: 29%	**Specificity**: 100%	**Accuracy**: 79%

Supplementary Table 6 | Matrix of all 24 patients with MR-RECIST and corresponding pathological response evaluation available. MR-RECIST underestimates the frequency of MPR.

MPR, major pathological response; PPR, partial pathological response; NPR, no pathological response; PR, partial response; CR, complete response; SD, stable disease; PD, progressive disease.

Supplementary Figure 1

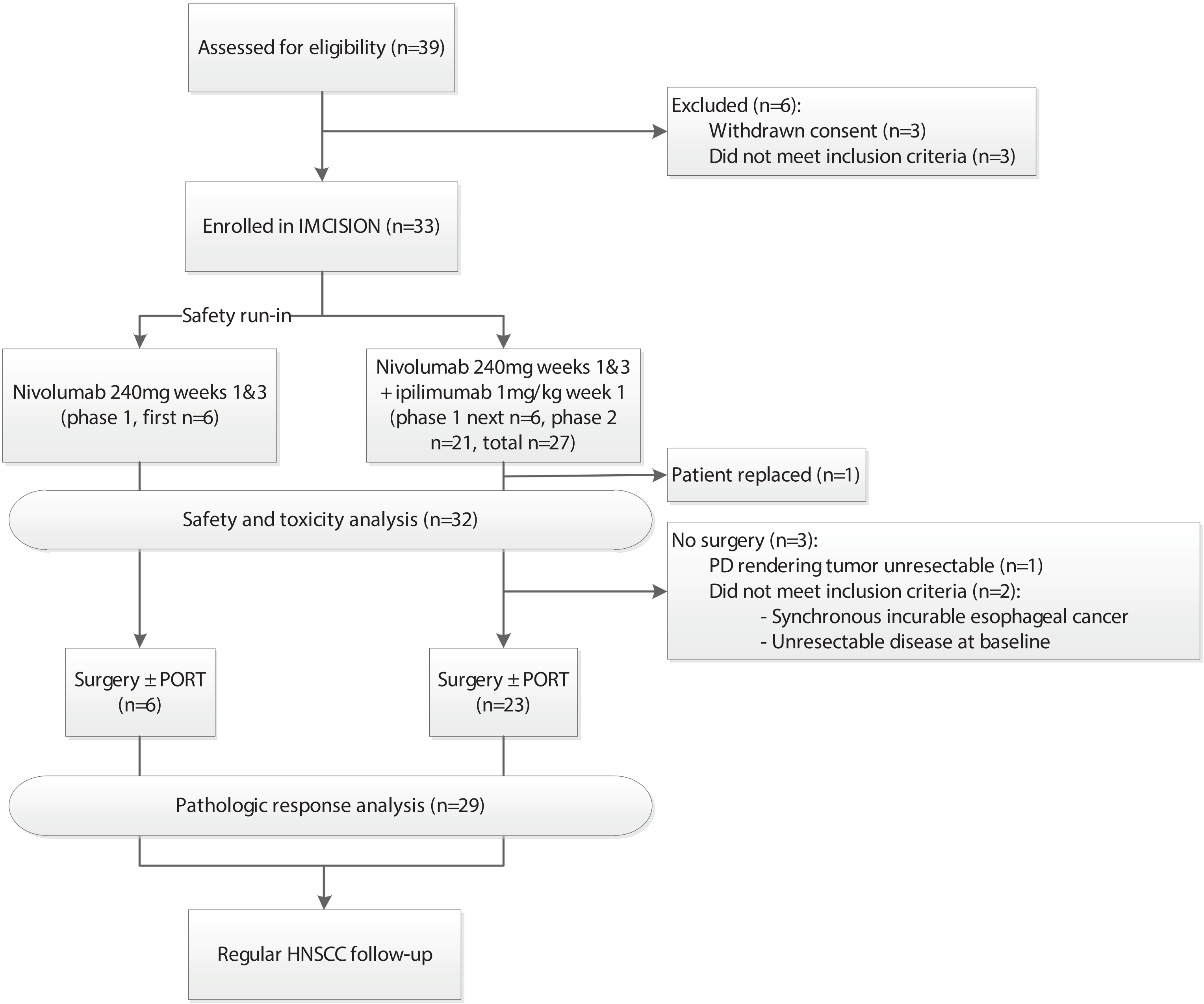

Supplementary Fig. 1| Flow chart of patients enrolled in IMCISION. Thirty-three patients were treated with neoadjuvant nivolumab (safety run-in, first n=6) or nivolumab + ipilimumab (all subsequent n=27 patients). One patient went off-study after receiving neoadjuvant nivolumab + ipilimumab and received nivolumab maintenance therapy at another institute. 32 patients were included in safety and feasibility analysis. Three patients did not undergo surgery, leaving 29 patients for pathologic response evaluation of the resected primary tumor and subsequent survival analyses.

Supplementary Figure 2

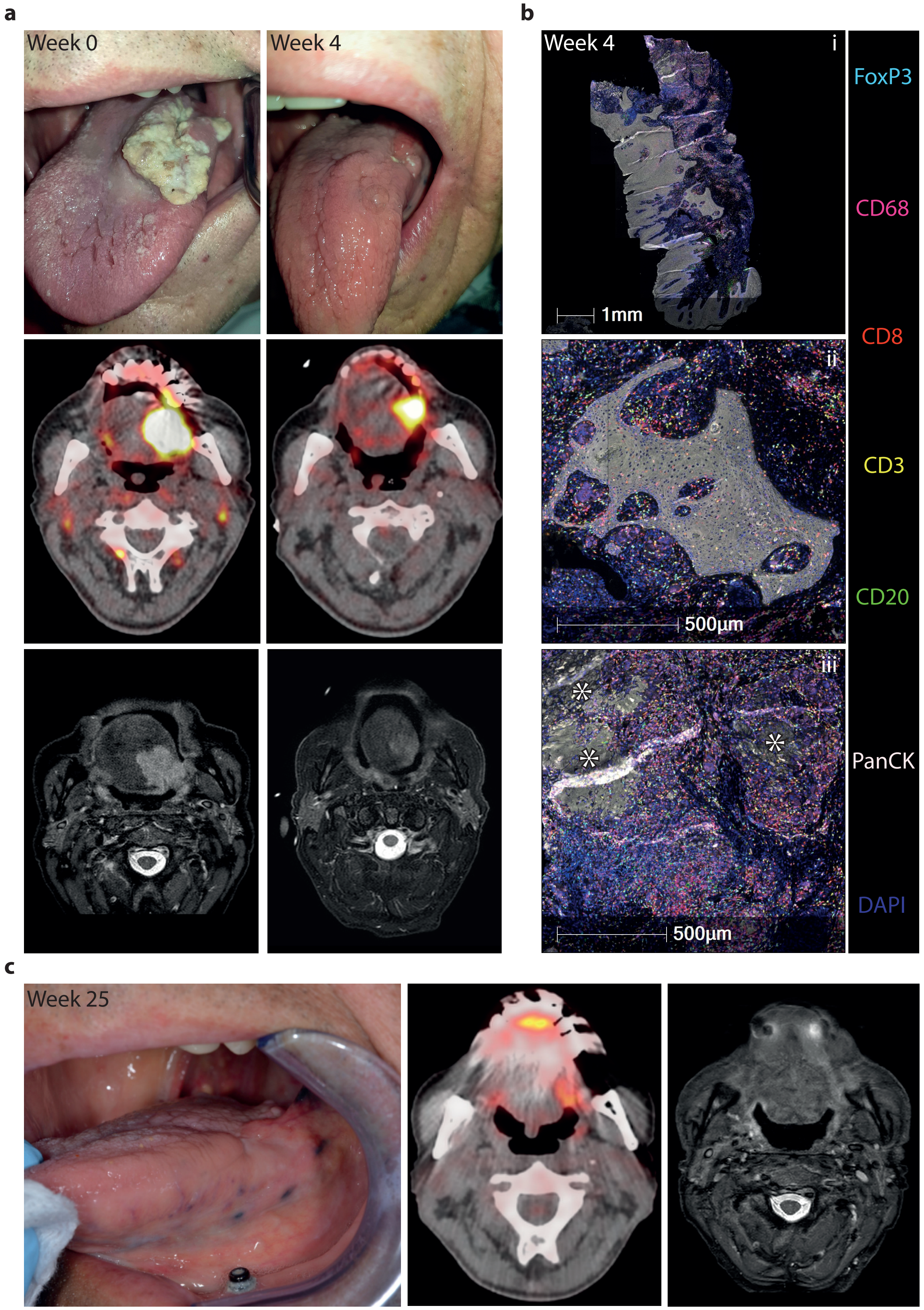

Supplementary Fig. 2| Clinical photography, imaging, and on-treatment biopsy of pt21, who did not undergo curative surgery due to synchronous, incurable esophageal carcinoma. a, Clinical response after two cycles of nivolumab + ipilimumab at the tongue carcinoma site (upper panels), accompanied by a 74% decrease in total lesion glycolysis assessed per FDG-PET (middle panels), and a partial response per MR-RECIST (bottom panels). **b**, Multiplex immunofluorescence histology of the on-treatment biopsy in week 4 (overview in **i**) reveals a small focus of residual viable tumor (shown in **ii**) and several areas of anuclear keratinous debris (marked with asterisks in **iii**). A dense infiltrate of CD8+ and CD68+ cells is present. **c**, The ICB response was ongoing when imaging was repeated in week 25. The tattoo ink in the clinical photograph (left panel) shows the primary tumor border, marked at baseline. The patient died of disseminated esophageal carcinoma without evidence of tongue carcinoma, 8 months after the second and last ICB cycle.

Supplementary Figure 3

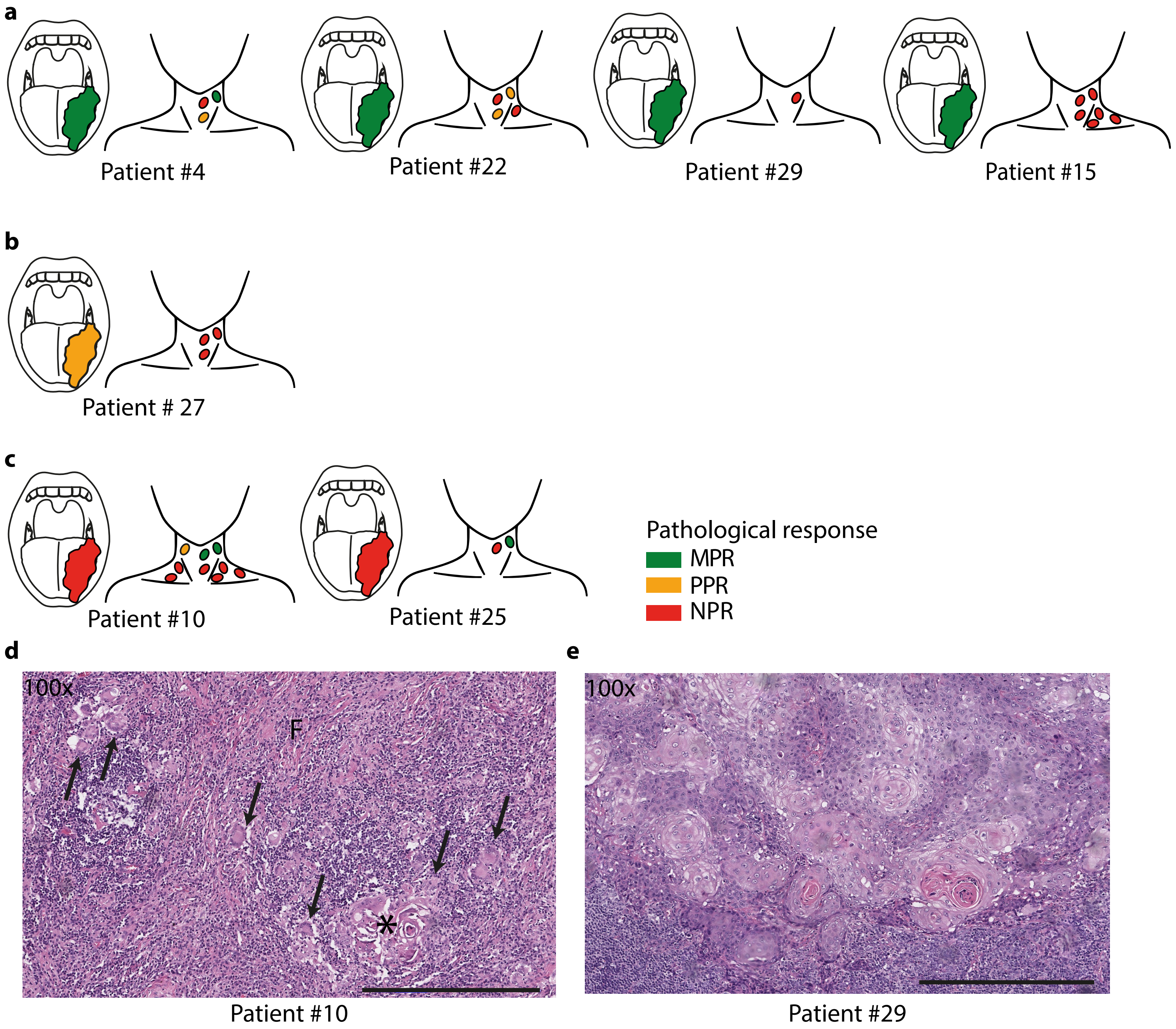

Supplementary Fig. 3| Dissociated treatment effect in primary tumors and lymph node metastases. a–c, Schematic representation of all 7 patients with a discordant ICB pathologic response (PR) at the primary tumor and lymph node metastatic site. **d,** Patient 10 had NPR at the primary tumor yet MPR at two lymph node sites, characterized by a substitution of normal lymph node architecture by fibrosis (F), keratinous debris (asterisk) and multinucleated giant cells (arrows). **e**, Viable lymph node metastasis showing no evidence of treatment response in pt29, who achieved 100% MPR (complete response) at the primary tumor site. Bars measure 0.5mm.

Supplementary Figure 4

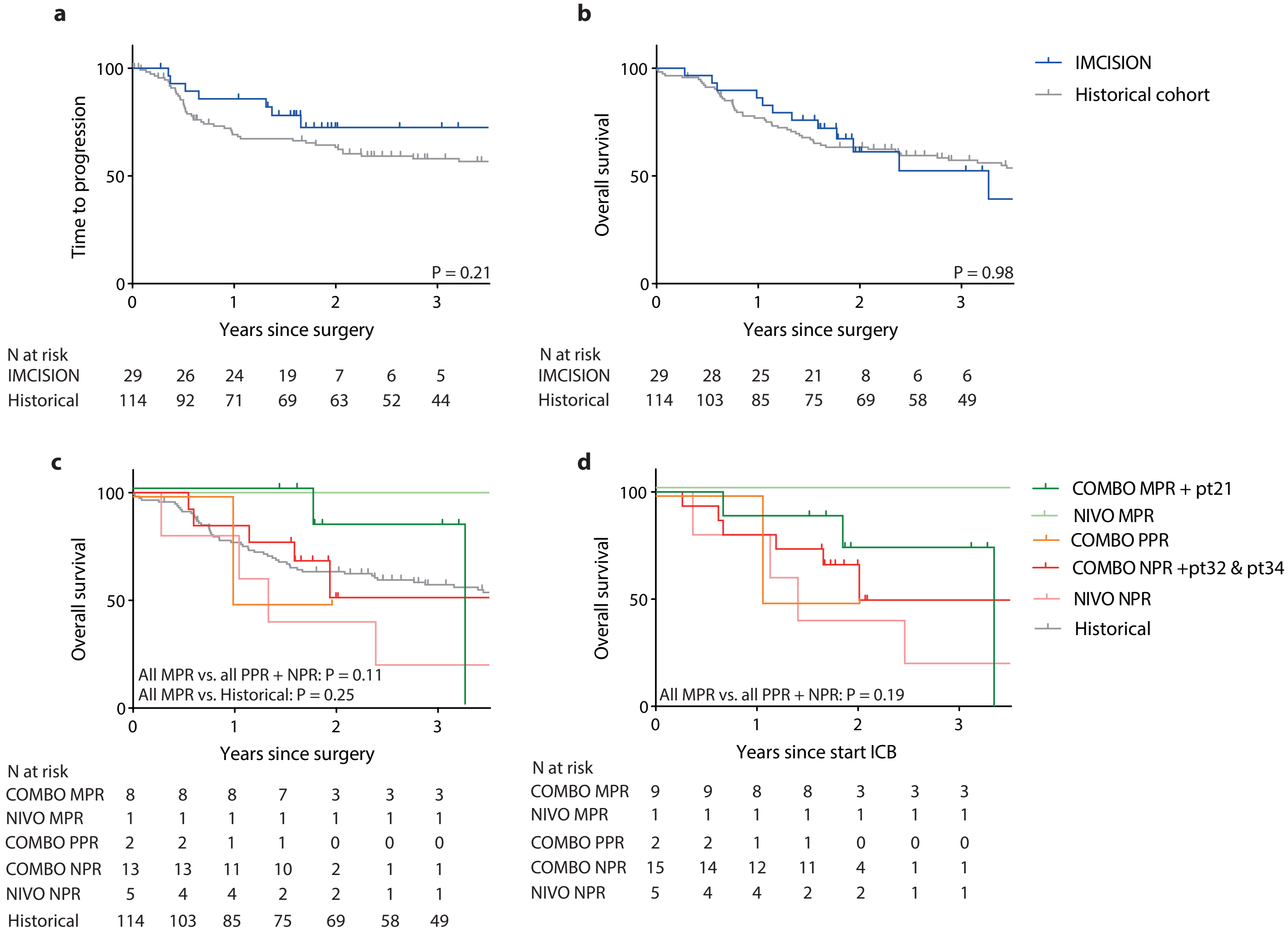

Supplementary Fig. 4| Kaplan-Meier survival estimates of IMCISION and historical HNSCC patients. a, Time to progression-analysis of the 29 IMCISION patients who underwent surgery (blue) and the historical cohort consisting of patients undergoing (salvage) surgery for locally advanced HNSCC without neoadjuvant treatment at our institute (gray). An exact *P*-value was calculated using a two-sided log-rank test. **b**, Overall survival of the 29 IMCISION patients who underwent surgery (blue) and the historical cohort (gray). An exact *P*-value was calculated using a two-sided log-rank test. **c**, Identical overall survival analysis as shown in **b**, but with the 29 IMCISION patients shown per neoadjuvant treatment and pathological response category. Exact *P*-values were calculated using a two-sided log-rank test. **d**, Additional overall survival analysis according to the intention-to-treat principle, of all 32 IMCISION that were treated with neoadjuvant ICB per treatment and per response group, including the 3 COMBO patients who did not undergo surgery and who were unevaluable for pathological response: 1 (pt21) with likely MPR and 2 (pt32 and pt34) with likely NPR. An exact *P*-value was calculated using a two-sided log-rank test.

Supplementary Figure 5

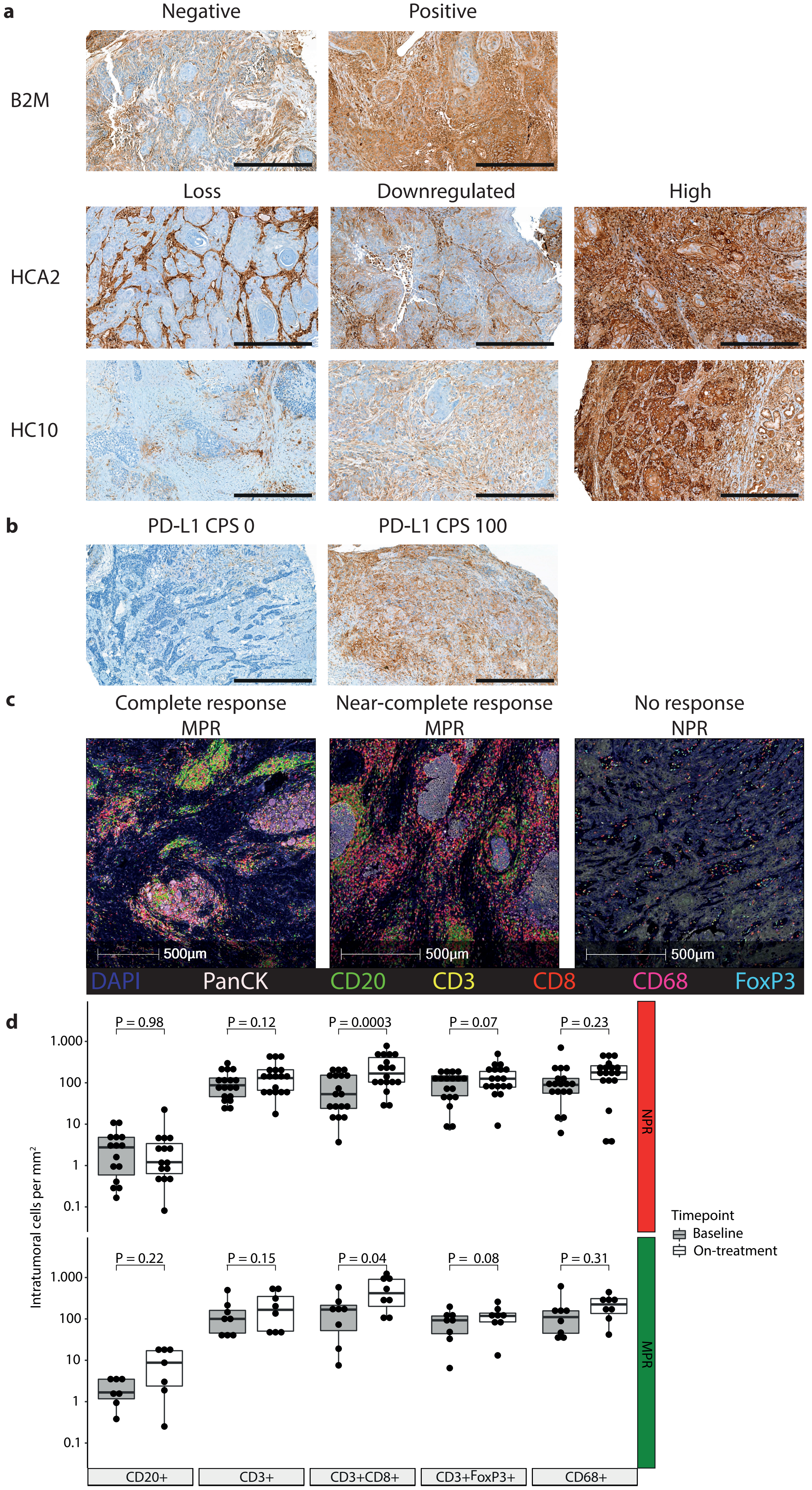

Supplementary Fig. 5| HLA and PD-L1 immunohistochemistry (IHC) and multiplex immunofluorescence (MPIF). **a**, Examples of expression of B2M, HCA2, and HC10, assessed per IHC as markers for HLA class 1 proficiency. All patients scored at least `downregulated' for HCA2 or HC10 or `positive' for B2M: all samples were thus considered proficient for HLA class 1. Bars measure 0.5mm. **b**, PD-L1 IHC and combined positive score (CPS) calculation, which varied from 0 (left) to 100 (right) in baseline tumor samples. Bars measure 0.5 mm. **c**, Examples of MPIF of tumor cells and five different immune cell populations performed on on-treatment surgical samples. Left and middle images show two samples with MPR: one complete response (left) and one near-complete response (middle). Right image shows a sample with no pathological response. **d**, Quantification of intratumoral immune cell densities at baseline and on-treatment, obtained through digital analysis of MPIF-stained slides. Exact *P*-values were calculated using a two-sided Wilcoxon signed-rank test. Only patients with paired pre- and on-treatment samples that contained tumor were included here: N = 26 pairs (8 MPR, 0 PPR, 18 NPR). Boxplots represent the median and 25th and 75th percentile, the whiskers extend from the hinge to the minimal and maximal data point but no further than 1.5x IQR.

Supplementary Figure 6

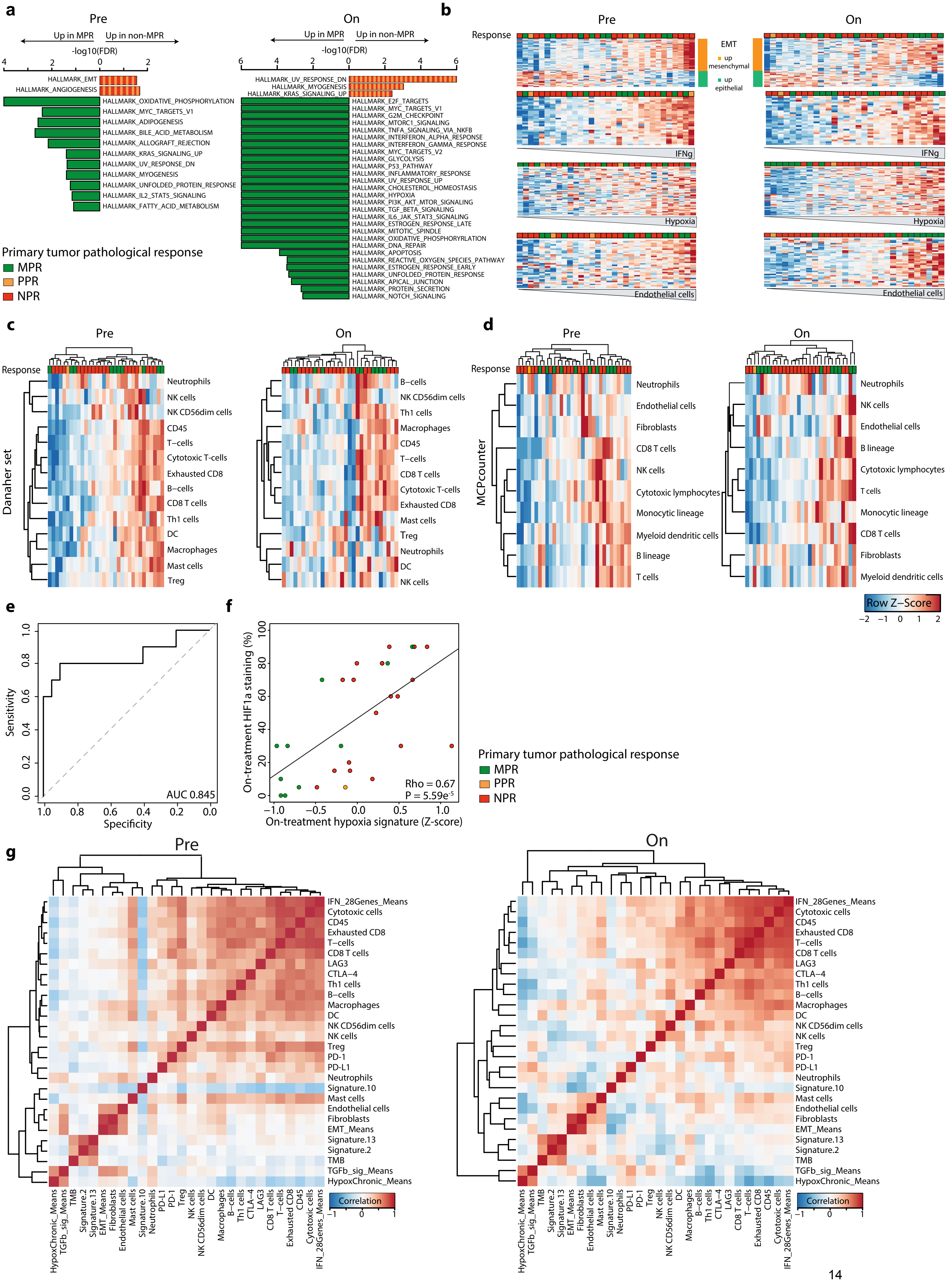

Supplementary Fig. 6| RNA sequencing of baseline and on-treatment primary tumor biopsies. a, Gene set enrichment analysis of baseline and on-treatment samples of MPR (green) and non- MPR patients (red). Source data are provided as a Source Data file. **b**, Heat maps showing baseline (left) and on-treatment (right) epithelial-to-mesenchymal-transition[1] (EMT, top), IFNγ[2], hypoxia[3], and endothelial cell[4] signature expression. Top bars represent ICB response category. Source data are provided as a Source Data file. **c**,**d**, Heat maps of baseline and on-treatment tumor immune cell infiltration based on the Danaher[5] (**c**) and MCPcounter[4] (**d**) set. Top bars represent ICB response category. Source data are provided as a Source Data file. **e**, Receiver operating characteristic (ROC) of hypoxia signature expression in on-treatment samples as a test for MPR. Source data are provided as a Source Data file. **f**, Two-tailed Spearman correlation of HIF-1α protein and hypoxia gene signature expression in on-treatment samples. Spearman's rho and an exact *P*-value is reported. Source data are provided as a Source Data file. **g**, Correlation matrix of baseline (left) and on-treatment (right) expression of multiple immune-related signatures. Color intensity shows the strength of positive (red) and negative (blue) correlations between gene expression signatures. Source data are provided as a Source Data file.

Patients unevaluable for pathological efficacy (n=3) are included based on their clinical response in this figure: 1 likely MPR and 2 likely NPR. In all panels, baseline (pre) N = 32 (10 MPR, 2 PPR, 20 NPR) and on-treatment (on) N = 30 (10 MPR, 1 PPR, 19 NPR).

Supplementary Figure 7

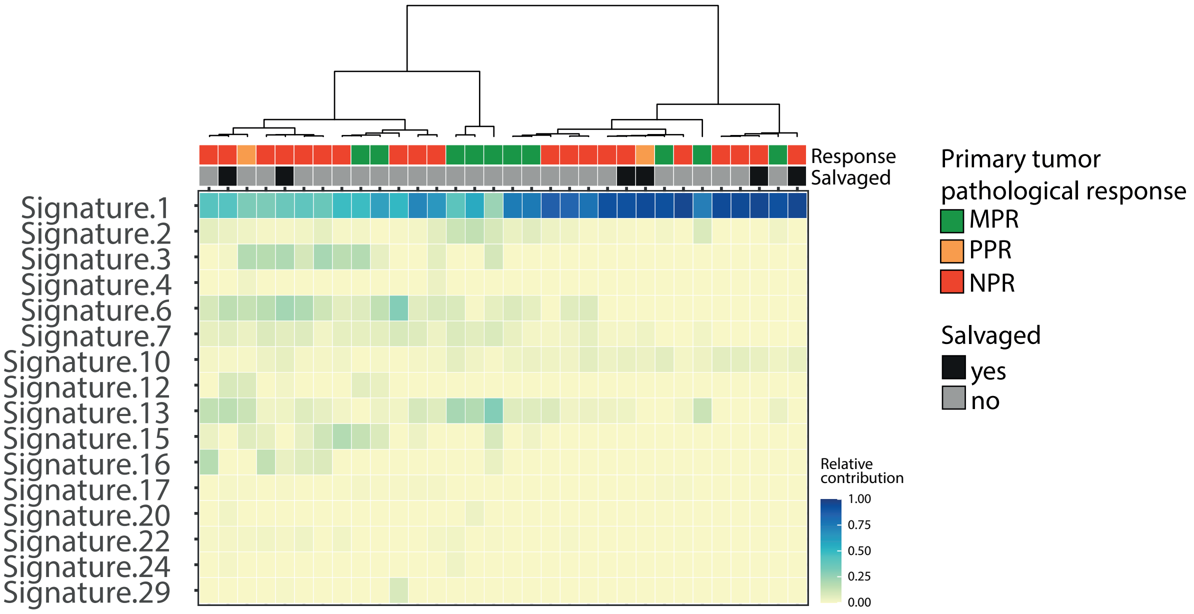

Supplementary Fig. 7| Baseline COSMIC[6] mutational profiles of primary tumor samples. COSMIC mutational signatures (rows) per baseline tumor samples (columns) assessed by whole-exome sequencing. Top bars indicate ICB response category and the previously irradiated tumors (`salvaged'). Patients unevaluable for pathological efficacy (n=3) are included based on their clinical response in this figure: 1 likely MPR and 2 likely NPR. N = 32 (10 MPR, 2 PPR, 20 NPR). Source data are provided as a Source Data file.

Supplementary Figure 8

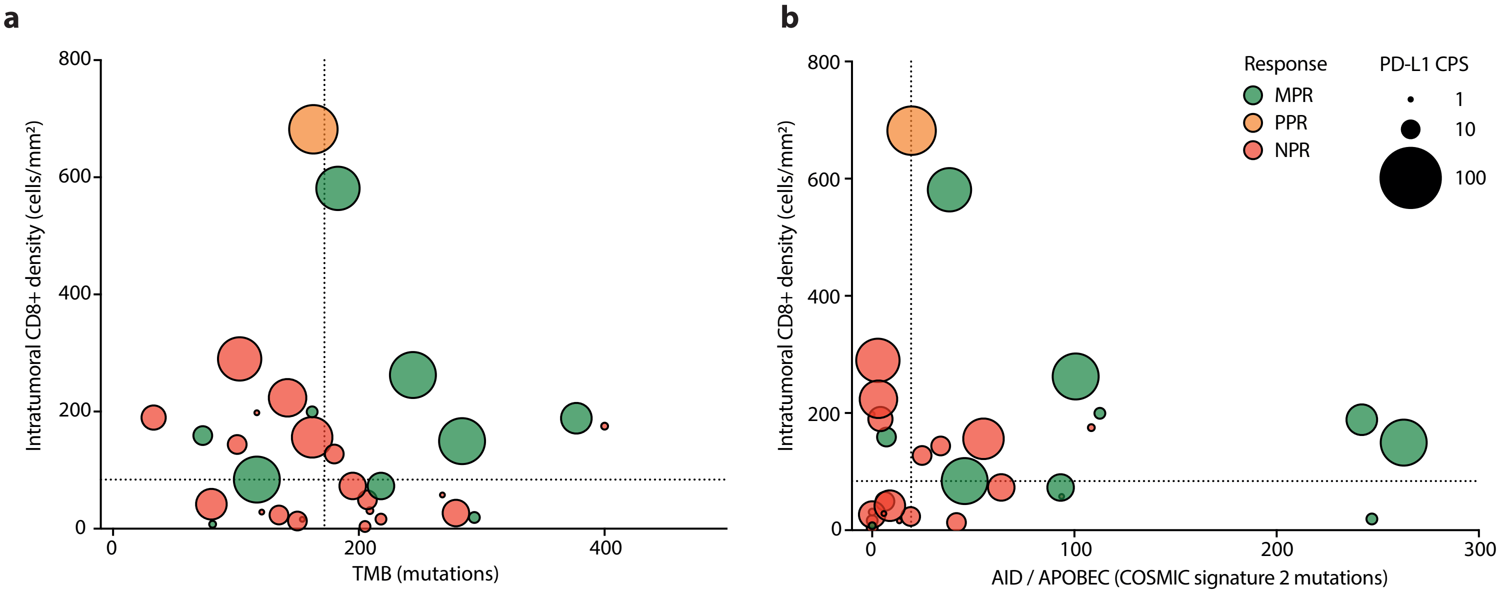

Supplementary Fig. 8| Combination of baseline biomarkers for ICB response. a, Bubble plot demonstrating baseline intratumoral CD3+CD8+ T-cell infiltration, TMB, and PD-L1 CPS in correlation with primary tumor pathological response. N = 31 (10 MPR, 1 PPR, 20 NPR). **b**, Bubble plot constructed with the same parameters as shown under **a**, but with TMB swapped for the number of COSMIC signature 2 (AID / APOBEC) -associated mutations. N = 31 (10 MPR, 1 PPR, 20 NPR).
Dotted lines represent medians. Source data are provided as a Source Data file. For visualization, one patient with CPS 0 (who had an NPR) is included here with CPS 1.

Supplementary References

1. Mak, M.P., *et al.* A Patient-Derived, Pan-Cancer EMT Signature Identifies Global Molecular Alterations and Immune Target Enrichment Following Epithelial-to-Mesenchymal Transition. *Clinical cancer research : an official journal of the American Association for Cancer Research* **22**, 609-620 (2016).
2. Ayers, M., *et al.* IFN-γ–related mRNA profile predicts clinical response to PD-1 blockade. *The Journal of Clinical Investigation* **127**, 2930-2940 (2017).
3. van der Heijden, M., *et al.* Acute Hypoxia Profile is a Stronger Prognostic Factor than Chronic Hypoxia in Advanced Stage Head and Neck Cancer Patients. *Cancers* **11**, 583 (2019).
4. Becht, E., *et al.* Estimating the population abundance of tissue-infiltrating immune and stromal cell populations using gene expression. *Genome Biology* **17**, 218 (2016).
5. Danaher, P., *et al.* Gene expression markers of Tumor Infiltrating Leukocytes. *Journal for ImmunoTherapy of Cancer* **5**, 18 (2017).
6. Alexandrov, L.B., *et al.* Signatures of mutational processes in human cancer. *Nature* **500**, 415-421 (2013).

Immuno**M**odulation by the **C**ombination of **I**pilimumab and nivolumab neoadjuvant to **S**urgery **I**n advanced **O**r recurrent Head and **N**eck Carcinoma

IMCISION, a phase-Ib/II trial

Version 7.1_2019

CA209-703, N16IMC

C.L. Zuur, MD, PhD, Head and Neck Surgeon
c.zuur@nki.nl

Netherlands Cancer Institute Antoni van Leeuwenhoek
Plesmanlaan 121, 1066 CX, Amsterdam, The Netherlands
0031-20-5122550

PROTOCOL TITLE

ImmunoModulation by the Combination of Ipilimumab and nivolumab neoadjuvant to Surgery In advanced Or recurrent Head and Neck Carcinoma (*IM*CISION)

**Protocol ID**	**CA209-703 / N16IMC**
**Short title**	IMCISION<br>Ipilimumab and nivolumab neoadjuvant to (salvage) surgery in HNSCC
**EudraCT number**	**2016_002366_31**
**Version**	**7.1**
**Date**	**26-02-2019**
**Coordinating investigator/project leader**	**Dr. C.L. Zuur**<br>**NKI AVL, Plesmanlaan 121**<br>**1066 CX, Amsterdam**
**Principal investigator(s) (in Dutch: hoofdonderzoeker/ uitvoerder)**	**Dr. C.L. Zuur**<br>Dr. J.P. de Boer<br>Drs. J.B.W. Elbers<br>Drs. J.L. Vos<br>Drs. A. van der Leun<br>Dr. A. Al-Mamgani<br>Dr. S.M. Willems<br>Dr. W.V. Vogel<br>Prof. Dr. M.W.M. van den Brekel<br>Dr. C.U. Blank<br>Prof. Dr. J.B.A.G. Haanen<br>Prof. Dr. T.N.M. Schumacher

**Sponsor (in Dutch: verrichter/opdrachtgever)**	**NKI-AVL**
**Subsidising party**	**Bristol-Myers Squibb**
**Independent expert (s)**	**Dr. M.S. van der Heijden, Medical Oncologist**<br>**NKI-AVL, Plesmanlaan 121**<br>**1066 CX Amsterdam**
**Laboratory sites**	**NKI AVL**
**Pharmacy**	**Pharmacy NKI AVL / Slotervaart Hospital**

TABLE OF CONTENTS

1. LIST OF ABBREVIATIONS AND RELEVANT DEFINITIONS 8

2. SUMMARY 10

2.1 Rationale 10

2.1.1 Advanced HNSCC 10

2.1.2 Checkpoint blockade neoadjuvant to standard of care 10

2.1.3 Translational biomarker research... 11

2.1.1 Hypoxia 11

2.1.2 Microbiome and metabolome 12

2.1.3 Measuring immunotherapy response in liquid biopsies 12

2.1.4 Single cell sequencing of matched patient samples 13

2.1.5 Dendritic cells in tumor-draining lymph nodes 13

2.1.6 In summary 14

2.2 Objectives 14

2.3 Study design 15

2.4 Study population and accrual time 15

2.5 Intervention 16

2.6 Study endpoints 19

2.7 Risk benefit assessment 20

3. INTRODUCTION AND RATIONALE 23

3.1 Current standard treatment 23

Neoadjuvant nivolumab and ipilimumab: A risk benefit assessment 23

3.2 23

3.3 Hypoxia as a potential biomarker in immunotherapy 26

3.4 Dysbiosis as a biomarker for anti-cancer treatment 29

3.5 Measuring immunotherapy response in liquid biopsies 31

3.6 Single cell sequencing of matched patient samples 32

3.7 Dendritic cells in tumor-draining lymph nodes 32

4. STUDY ENDPOINTS 34

5. STUDY POPULATION 36

5.1 Population (base) 36

5.2 Inclusion criteria 36

5.3 Exclusion criteria 37

6. INVESTIGATIONAL PRODUCT 39

6.1 Name and description of investigational product(s) 39

6.2 Description and justification of route of administration and dosage ... 39

6.3 Dosages, dosage modifications and method of administration 39

6.4 Dose Delay Criteria 40

6.5 Criteria to Resume Treatment 42

6.6 Discontinuation Criteria 43

6.7 Treatment of Nivolumab or Ipilimumab Related Infusion Reactions 44

6.8 Preparation and labeling of Investigational Medicinal Product............. 46
6.9 Drug accountability 46
6.10 Use of co-intervention 46
6.10.1 Prohibited medication 46
6.10.2 Permitted medication 46
6.10.3 Contraception 47

7. TUMOR RESPONSE ANALYSIS / TRANSLATIONAL RESEARCH................ 48
7.1 Imaging.. 48
7.1.1 Multi-parametric MRI.. 48
7.1.2 FDG-PET 48
7.2 Tissue samples and assays 48
7.2.1 T cell capacity 52
7.2.2 Tumor immune infiltrates 52
7.2.3 Tumor cytokine production.. 52
7.2.4 Monitoring tumor tissue hypoxia. 53
7.2.5 Tumor pathological response to neoadjuvant immunotherapy.............. 53
7.2.6 Dysbiosis as a biomarker for response to immunotherapy 54
7.3 Tattoo tumor delineation 55
7.4 Sample assays: PBMCs, serum, microbiome, ctDNA and single cell-omics 55
7.4.1 PBMCs.. 55
7.4.2 Serum / Luminex.. 55
7.4.3 Microbiome and metabolome.. 55
7.4.4 ctDNA in liquid biopsies 56
7.4.5 Single cell sequencing of matched patient samples.............................. 56
7.4.6 Dendritic cells in tumor-draining lymph nodes....................................... 57

8. STUDY METHODS 58
8.1 Study endpoints 58
8.2 Screening phase.. 59
8.3 Off study criteria.. 60
8.4 Randomization, blinding and treatment allocation................................ 60
8.5 During treatment.. 60
8.6 Post treatment evaluation... 63
8.7 Withdrawal of individual subjects... 63
8.8 Replacement of individual subjects after withdrawal 63
8.9 Follow-up of subjects withdrawn from treatment................................... 63
8.10 Premature termination of the study... 64

9. SAFETY REPORTING.. 65
9.1 Section 10 WMO event 65
9.2 AEs, SAEs and SUSARs 65
9.2.1 Adverse events (AEs) 65
9.2.2 Serious adverse events (SAEs) 67

9.2.3 Reporting of SAEs 68
9.2.4 Suspected unexpected serious adverse reactions (SUSARs) 69
9.3 Annual safety report 71
9.4 Follow-up of adverse events 71
9.5 Data Safety Monitoring Board (DSMB) / Safety Committee 71
10. STATISTICAL ANALYSIS 72
10.1 Sample size calculation 72
10.2 Safety analysis 73
10.3 Immune- and pathologic response analysis 73
10.4 Clinical response evaluation 73
10.5 Interim analysis 73
11. ETHICAL CONSIDERATIONS 75
11.1 Regulation statement 75
11.2 Recruitment and consent 75
11.2.1 Informed consent (IC) 75
11.2.2 Recruitment 75
11.3 Benefits and risks assessment 76
11.4 Compensation for injury 76
11.5 Incentives 76
12. ADMINISTRATIVE ASPECTS, MONITORING AND PUBLICATION 77
12.1 Subject identification 77
12.2 Randomization of the patients 77
12.3 Storage and coding of patient material 77
12.3.1 Tissue samples 77
12.3.2 Lymph node samples 78
12.3.3 Blood samples 78
12.3.4 Microbiome and metabolome 79
12.3.1 ctDNA in liquid biopsies 79
12.3.2 Single-cell sequencing 79
12.3.3 cDC1 cells in tumor tissue and tumor-draining lymph nodes 79
12.4 Data management 79
12.5 Monitoring and Quality Assurance 80
12.6 Amendments 80
12.7 Annual progress report 80
12.8 End of study report 80
12.9 Public disclosure and publication policy 81
13. REFERENCES 82
14. APPENDIX 86
14.1 Safety algorithms 86
14.2 ECOG Performance Status Score Definition 93
14.3 Common Terminology Criteria for Adverse Events (CTCAE) 94

14.4 Response Evaluation Criteria in Solid Tumors (RECIST) 95

1. LIST OF ABBREVIATIONS AND RELEVANT DEFINITIONS

ABR	ABR form, General Assessment and Registration form, is the application form that is required for submission to the accredited Ethics Committee (In Dutch, ABR = Algemene Beoordeling en Registratie)
AE	Adverse Event
AR	Adverse Reaction
BMS	Bristol Myers Squibb
CA	Competent Authority
CCMO	Central Committee on Research Involving Human Subjects; in Dutch: Centrale Commissie Mensgebonden Onderzoek
CTLA-4	cytotoxic T-lymphocyte-associated protein 4
DFS	Disease Free Survival
DSMB	Data Safety Monitoring Board
EU	European Union
EUA	Examination Under Anesthetics
EudraCT	European drug regulatory affairs Clinical Trials
GCP	Good Clinical Practice
HNSCC	Head and Neck Squamous Cell Carcinoma
IB	Investigator’s Brochure
IC	Informed Consent
IHC	immunohistochemistry
irAE	Immune related adverse event
IMP	Investigational Medicinal Product
IMPD	Investigational Medicinal Product Dossier
IT	Immunotherapy
METC	Medical research ethics committee (MREC); in Dutch: medisch ethische toetsing commissie (METC)
PD-1	Programmed death receptor-1
(S)AE	(Serious) Adverse Event
SOC	Standard Of Care
SPC	Summary of Product Characteristics (in Dutch: officiële productinfomatie IB1-tekst)
Sponsor	The sponsor is the party that commissions the organisation or

	performance of the research, for example a pharmaceutical company, academic hospital, scientific organisation or investigator. A party that provides funding for a study but does not commission it is not regarded as the sponsor, but referred to as a subsidising party.
SUSAR	Suspected Unexpected Serious Adverse Reaction
TIL	Tumor Infiltrating Lymphocytes
Wbp	Personal Data Protection Act (in Dutch: Wet Bescherming Persoonsgevens)
WMO	Medical Research Involving Human Subjects Act (in Dutch: Wet Medisch-wetenschappelijk Onderzoek met Mensen

2. SUMMARY

2.1 Rationale

2.1.1 Advanced HNSCC

Head and neck squamous cell carcinoma (HNSCC) is the 7th most common cancer in men and the 9th most common cancer in women. In 2011, 3000 patients were diagnosed with head and neck cancer in the Netherlands [1]. In advanced stage oral cavity carcinoma and salvage surgery after failed (chemo)radiation, patients generally suffer extensive mutilating surgery, and nevertheless have a very poor prognosis of 37% 5-year overall survival in stage IV oral HNSCC [2] and 20-40% 2-year overall survival after salvage surgery [3]. Although multiple (neo)adjuvant chemotherapeutic regimens have been evaluated, clinical benefit fails to appear [4].

2.1.2 Checkpoint blockade neoadjuvant to standard of care

T cell checkpoint blockade by anti-CTLA and/or anti-PD1 is currently the most promising in immunomodulation anticancer therapies. In HNSCC, pembrolizumab (anti-PD1 monoclonal antibody) given at a fixed dose of 200 mg every 3 weeks was well tolerated and demonstrated a clinically meaningful overall response rate of 24.8% in patients with recurrent/metastatic disease, irrespective of HPV status [5]. In addition, biweekly Nivolumab 3 mg/kg in recurrent or metastatic setting of HNSCC has doubled the 1 year survival rate from 16% to 36% [6].

The rationale behind combining aPD1 and aCTLA4 is that nivolumab and ipilimumab enhance T-cell antitumor activity through distinct but complementary mechanisms resulting in both enhanced T-cell priming and enhanced local T-cell-mediated tumor destruction. The complementary effect of both checkpoint inhibitors was first proven in a phase III trial treating metastatic melanoma with response rates of 58% [7]. Shortly after, a study involving thirty-nine stage IIIB/IV Non-Small Cell Lung Carcinoma patients treated with nivolumab 3 mg/kg and ipilimumab 1 mg/kg, 8 and 2 infusions respectively, and an overall treatment period of 15-18 weeks, resulted in 31% durable response rates [8].

Moreover, very recently, it was postulated that offering the combination of nivolumab and ipilimumab in neo-adjuvant setting would exert even stronger immunomodulation and increased tumor responses to treatment, when compared to adjuvant immunotherapy. One hypothesis is that the presence of tumor load before surgery offers increased neo-antigen presentation with consequently more efficient T-cell receptor triggering. In addition, reduced tumor heterogeneity, as compared to treatment in the metastatic setting, and improved immune status during earlier disease status are factors that are likely to positively influence the efficacy of immunomodulation in this setting. Indeed, recent, early clinical data evaluating the activity of neo-adjuvant nivolumab and ipilimumab in stage III melanoma provide support for this hypothesis [9].

2.1.3 Translational biomarker research

Although immunotherapy has proven to be effective in various tumor types, a reliable predictive biomarker for treatment response does not exist. Hypoxia is a well-known biomarker for treatment response (RT, chemotherapy and surgery) in various solid tumors (e.g. lung, kidney, HNSCC) and a vast amount of preclinical data indicates a key role for hypoxia on T cell (both CD4 and CD8) metabolism, fate and function. Therefore, hypoxia may be a clinical biomarker for tumor response to immunotherapy in solid cancers in general. See paragraph 3.3 for an overview of preclinical literature on tumor hypoxia in detail.

2.1.1 Hypoxia

Tumor hypoxia will be assessed by RNA expression hypoxia signatures developed over the years [10-12]. Such signatures are used to identify patients for selective treatment to overcome hypoxia. Recently, a 15-gene hypoxia classifier was validated in 323 patients with HNSCC randomized for hypoxic modification or placebo in combination with radiotherapy. Tumors categorized as hypoxic on the basis of the classifier were associated with a significantly poorer clinical outcome than non-hypoxic tumors [13]. In addition, technical validation of the 15-gene hypoxia classifier demonstrated that it is suitable for implementation in prospective clinical trials as well [14].

2.1.2 Microbiome and metabolome

Another potential biomarker can be found in the human microbiota. Microbial imbalance (dybiosis) on or inside the body may affect oncogenesis, tumor progression and response to cancer therapy [15, 16]. Microbes present at mucosal sites may exert different immune-modulatory effects. Segmented filamentous bacteria, which can breach the gut mucus layer and attach to intestinal epithelial cells (IECs) are a potent inducer of T helper 17 cells and for instance Helicobacter Pylori may lead to an increase in loco-regional T-regs [17, 18]. During cancer therapy, radiation or drug regimens may exert toxic effects on bacteria, thereby promoting dysbiosis. On the other hand, (gut) microbiota influences the therapeutic efficacy and side effects of cancer drugs via pharmacodynamics and immunological mechanisms [15, 16]. Indeed, recent work has shown that gut microbiota interfere with tumor responses to chemotherapy and aPD1 or aCTLA4 immunotherapy [17, 19, 20]. In HNSCC, the oral cavity harbors more than 700 bacterial species and is one of the most densely populated areas of the human body. Differences in bacterial- and viral composition have been described for oral cancers in patient series [21], potentially serving as biomarker for oral cancer. The microbiome, its metabolome, and its potential effect on cancer treatment in HNSCC was never studied before. For translational research purposes.

2.1.3 Measuring immunotherapy response in liquid biopsies

Levels of circulating tumor DNA (ctDNA) in 'liquid biopsies' (e.g. blood, saliva) could serve as a novel, non-invasive biomarker for determining response to treatment [22-25]. 80-90% of non-HPV HNSCC patiënts carry TP53 mutations, which can be detected in ctDNA [26, 27]. Therefore, screening for changes in the levels of TP53 mutations in the blood of HNSCC-patients which receive immunotherapy may provide an ideal biomarker for predicting treatment response [28]. Low levels of TP53 mutations can be reliably detected among the other cell-free DNA using a new technique, Cyclomics, which is based on Oxford Nanopore MinION sequencing of concatenated copies of a single DNA molecule. We will use this technique to determine the levels of TP53 mutations in the blood of 20 stage III-IV HNSCC patients with a TP53 mutation both prior to and after immunotherapy. By comparing these TP53 mutation levels with e.g. MRI scans, we aim to dissect whether a liquid

biopsy provides a more accurate (and less invasive) prediction for immunotherapy response.

2.1.4 Single cell sequencing of matched patient samples

Whereas bulk sequencing efforts lack the depth and sensitivity to detect disease mechanisms in heterogenous cell populations, single-cell RNA sequencing is able to identify transcriptional changes on a single-cell basis. This provides insight into how cells interact, and may identify previously overlooked cell types involved in disease development, disease progression and the interaction with the immune system, including in HNSCC patients [29]. In addition, single-cell sequencing of matched patient samples before and after neoadjuvant immunotherapy may efficiently identify responding cell populations and, on a larger scale, patients who are more likely to benefit from immunotherapy [30]. This might provide a rationale for future trial and drug design. We will use single-cell sequencing technology to analyze our patient's tumor samples at baseline, after immunotherapy and in the case of tumor recurrence.

2.1.5 Dendritic cells in tumor-draining lymph nodes

The role of Type 1 conventional dendritic cells (cDC1) in tumor-draining lymph node (tdLN) is to cross-present tumor antigens and prime anti-tumor CTL response, and in the tumor microenvironment (TME) is to re-stimulate tumor infiltrating T cells and help to sustain the tumor-specific cytotoxic T lymphocyte (CTL) response in tumor site locally, which explains why the abundance of cDC1s in TME is positively correlates with the responsiveness to anti–PD-1 therapy and results in a better survival of cancer patients (Böttcher *et al.* The Role of Type 1 Conventional Dendritic Cells in Cancer Immunity. *Trends in Cancer* 2018). Our preliminary study indicates that the human cDC1 is the most capable DC subset in relaying the "help" signal from the $CD4^+$ T cell to generate a tumor-specific CTL response (Xiao *et al.* unpublished data). Therefore in addition of monitoring tumor infiltrating CD8 T cells, macrophages and MDSCs (as described in 7.2.2 of the IMCISION proposal), the presence of cDC1 and activated CD4 T cells and their phenotype in tdLN and TME also need to be measured. This information could be an indicator for identifying the responder patients.

2.1.6 In summary

Herewith, we present a research protocol that allows us to examine feasibility and safety of checkpoint blockade neoadjuvant to standard of care (SOC) in a patient population in need for improved clinical outcome and in tumors likely to respond to neoadjuvant aPD1 and aCTLA4. In addition, with this research protocol we can assess the potential impact of intratumoral hypoxia on tumor infiltrating lymphocyte (TIL) abundance, differentiation and effector function, and the potentially divergent effects of T cell checkpoint blockade in hypoxic versus normoxic tumors. Also, we aim to assess the oral and gut microbiome (comprising bacteria, archea, viruses and fungi) and the oral metabolome profile in patients with advanced HNSCC before and after anti-cancer immune therapy. Finally, we aim to investigate whether single cell sequencing and/or ctDNA and/or dendritic cells in lymph nodes (only after immunotherapy) obtained before and after immunotherapy may serve as a biomarker for treatment response.

2.2 Objectives

Primary objectives:

- Phase Ib: Primary objective is feasibility and safety (NCI CTCAE v4.0) of the administration of nivolumab with or without ipilimumab neoadjuvant to standard of care in terms of SAEs and adherence to the timelines as described for SOC (see Figure 1).
- Phase II: Tumor response to neoadjuvant IT in terms of tumor tissue pathological response at time of surgery compared to RECIST 1.1 (FDG-PET and MRI)

Secondary objectives:

- We will monitor immune cell subsets and cytokines in the peripheral blood and tumor compartment.
- Rate and type of late AEs (NCI CTCAE v 4.0) up to 2 years follow-up after SOC (see Figure 1).
- Relapse free survival (RECIST 1.1) and overall survival at 2 years follow-up
- To evaluate tumor hypoxia and its correlation with treatment response and disease outcomes.

- To evaluate the oral and gut microbiome (comprising bacteria, archea, viruses and fungi) and the oral metabolome profile in patients with advanced HNSCC before and after anti-cancer immune therapy.
- To evaluate ctDNA and its applicability as a biomarker in patients with advanced HNSCC before and after immunotherapy.
- To evaluate single-cell sequencing of tumor material and its applicability as a biomarker in patients with advanced HNSCC before and after immunotherapy.
- To determine the abundance of cDC1 cells in tumor tissue and draining lymph nodes.

Note: For specific **endpoints**, see paragraph 2.6 of this protocol.

2.3 Study design

This is a Phase 1b/II trial. For the design see figure 1.

The **phase Ib** is designed as 3 + 3, with primary objective feasibility and toxicity.
Of Note: we wish to see endpoints reached in all 6 patients of cohort 1 and 2, before we will continue to the next cohort.

The **phase II** is designed as a single arm design with primary endpoint efficacy.

In **phase Ib**, two cohorts will be used (cohort 1: nivolumab only and cohort 2: nivolumab and ipilimumab neoadjuvant to surgery) to define which neoadjuvant immunotherapy regimen will be taken towards the expansion cohort 3.

Thirty-two patients will be treated with nivolumab (240 mg flat dose, week 1 and week 3, twice in total) as a single agent OR the combination of ipilimumab (1 mg/kg) + nivolumab (240mg flat dose) in week 1, and nivolumab 240mg flat dose in week 3, neoadjuvant to SOC (surgery with or without adjuvant (C)RT).

2.4 Study population and accrual time

1. Patients naïve for immunotherapy, more than 18 years old, and
2. Patients with histologically confirmed T2-4N0-3M0 HNSCC of the oral cavity, oropharynx, hypopharynx or larynx, eligible for major curative surgery (e.g.

COMMANDO-procedure, laryngectomy) as primary treatment or major salvage surgery after failed (chemo)radiation.

We intend to include 32 patients in 2.5 year time.

2.5 Intervention

For a timeline, see figure 1.

Patients will be treated with

- 2x nivolumab 240 mg flat dose, weeks 1 and 3, OR
- the combination of 1x ipilimumab 1 mg/kg + nivolumab 240 mg flat dose in week 1 and nivolumab mono-therapy 240 mg flat dose in week 3.

Immunotherapy (IT) is given neoadjuvant to standard of care (SOC: surgery with or without adjuvant (C)RT). Surgery will be planned in week 5. Adjuvant (C)RT will start 4-6 weeks after surgery.

Figure 1: Study design and timeline of diagnosis, screening, treatment, study assays and follow-up

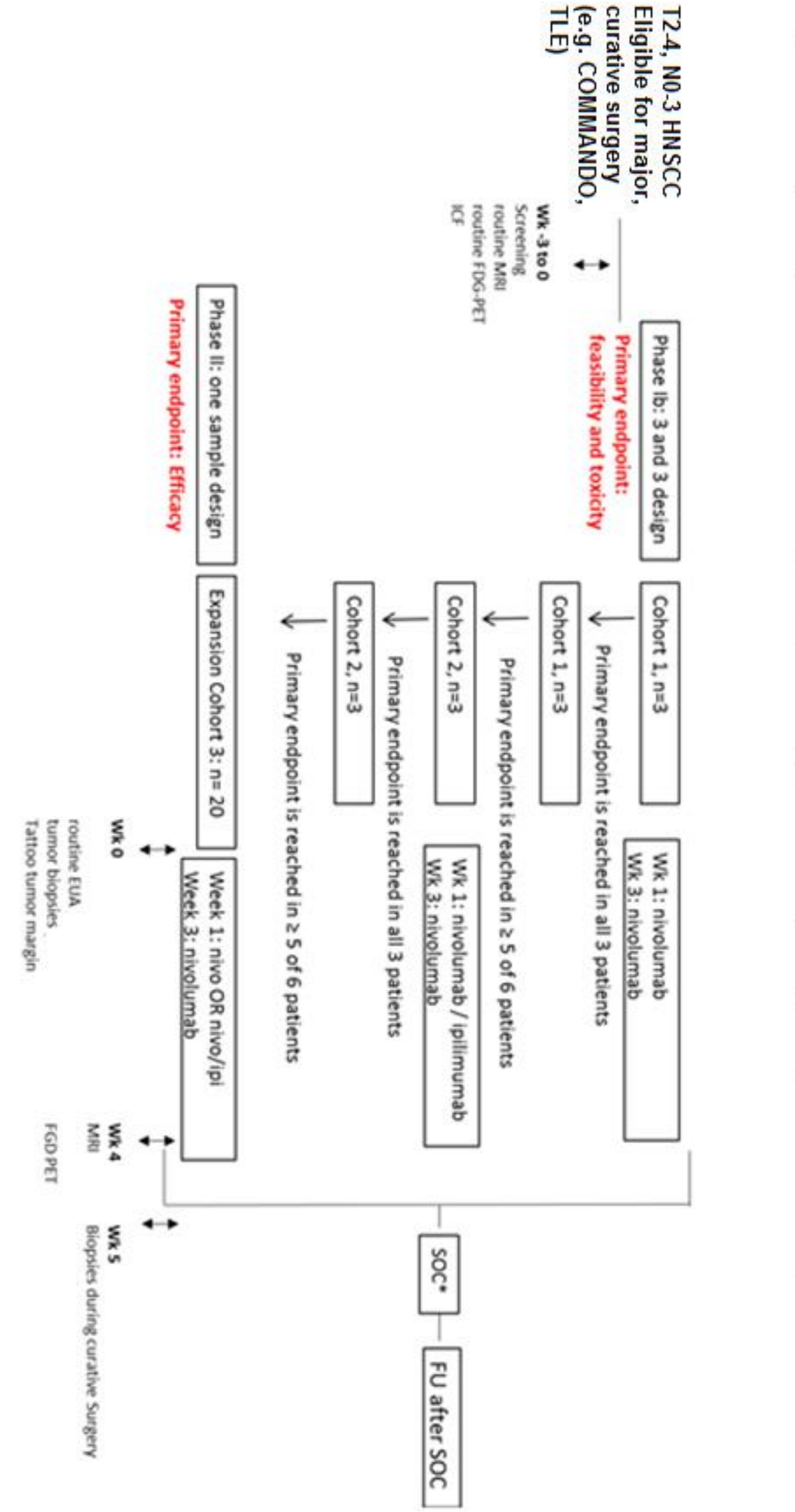

SOC*=Standard of care: surgery w/wo adjuvant (C)RT, surgery is planned in week 5, adjuvant (C)RT will be planned 4-6 weeks after surgery.

Research treatment and assessments are shown in table 1. Alterations added in version 7.1 of the research protocol have been marked yellow.

Table 1. Detailed routine (white) and research (blue) treatment and assessments

	routine work-up and screening	week -1	week 1-3	week 4-5	week 5	2 year FU after SOC[1,2]	
**Treatment**			Nivo or Nivo-Ipi		SOC: surgery w/wo (C)RT		
Medical history	x						
Physical examination[3]	x		x		x	4x	3x
ECG	x						
Examination under anesthesia (EUA)		x					
Signing ICF	x						
Blood collection							
a. Hematology[4]	x		x		x	4x	4x[11]
b. Chemistry[5]	x		x		x	4x	4x[11]
c. Serology[6]	x						
d. beta-HCG pregnancy test[7]	x		x				
e. Coagulation: PT/INR and aPTT	x						
f. PBMC[8]		x	x				4x[11]
g. Luminex[9]		x	x				4x[11]
h. ctDNA blood sample[13]		x			x		
Imaging							
a. MRI	x			x			
b. FDG PET	x			x			
Biopsies							
a. Tumor (2x8mm)[10]	x	x			during curative surgery	x[12]	x[12]
b. Benign (1x2mm)		x					
c. Tumorous lymph node (half)14					during curative surgery		
d. Benign lymph node (half)					during curative surgery		
Microbiome and metabolome							
a. Oral swabs		x			x		
b. Faeces		x		x			
Adverse events			x		x		7x

1: Standard Of Care (SOC) is surgery w/wo (C)RT. Adjuvant (C)RT will be planned 4-6 weeks after surgery. FU starts after SOC.
2: For study purposes FU of 2 years after SOC is needed. No extra visits are needed for research.
Routine FU: Year 1: every 3 months. Year 2: every 4 months. In total 7 visits in 2 years.
3: ECOG performance status, weight, temperature, pulse, blood pressure
4: Hb, ANC, platelet count incl. differentiation, Hct, 3 mL total
5: LDH, phosphorus, sodium, potassium, magnesium, chloride, calcium, creatinine, albumin, total protein, SGOT (AST), SGPT (ALT), bilirubin (ind + dir), GGT, alkaline phosphatase, glucose, lipase, TSH, fT4, ACTH, cortisol, LH, FSH, testosterone/oestradiol, S100, CRP, ESR. 5 mL total.
6: HIV, HbsAG, HCV, anti-CMV, HSV, EBV and lues; 10 mL total.
7: Only WOCBP, goes with chemistry, no extra mL blood needed.
8: At screening 100mL, all other samples 50mL
9: 5mL (serum bottle)
10: 1x 8mm biopsy will be stored here, the other will be halved with half sent to the USA for analysis by Celsius and other to be stored here
11: One blood draw will be taken directly 1 week after sugery
12: In routine care, 1 biopsy is taken for diagnosis in case of disease progression. Patients will be asked for 1 extra biopsy of 2mm for research (not obligatory).
13: ctDNA blood sample 10 mL, before immune therapy and after immune therapy, 20 mL in total per patient
14: Patients will be asked for half of a tumorous lymph node and half of a benign node. This is not obligatory. Nodes will be obtained via standard-of-care neck dissection.

Notes:
I: at screening, 13 mL extra blood is needed (coagulation and serology. For pregnancy test no extra mL blood is needed).
II: during the study, the first time 105 mL extra blood is needed (PBMC 100 mL, Luminex 5 mL), for the second infusion 63mL.
Thereafter, at 4 time points extra blood is needed in more than 2 years time: 63 mL (PBMC 50, Luminex 5, Chemistry 5, Hematology 3)
III: In total, for study purposes in more than 2 years time 453 mL extra blood is needed.
IV: In total patients will undergo 7 extra blood draws for study purposes: week -1, twice before immunotherapy infusion, and 4 times during 2 years FU.
These blood draws are taken during (study) treatnment or routine FU. Patients do not need to come extra for this.

2.6 Study endpoints

Primary endpoints:

- Phase Ib: Primary endpoint is measured as the number of patients that will not endure a delay in surgery (surgery should be performed in week 5-6) due to neoadjuvant immunotherapy (nivolumab, ipilimumab) related toxicity (measured in terms of SAEs and CTCAE v4.0) OR the treatment of immunotherapy related toxicity (ie high dose corticosteroids)**.
  ** To meet this endpoint, all patients will be discussed in our immunotherapy team meeting (consisting of at least medical oncologist and head and neck surgeon) the week before surgery, to evaluate whether immunotherapy-related toxicity or treatment of immunotherapy-related toxicity will lead to delay in surgery or not.
  ** Delay in surgery due to logistical problems (i.e. no IC bed after surgery) or other co-morbidity (i.e. bacterial pneumonia) will not be considered dose-limiting toxicity.
- Phase II: Tumor response to neoadjuvant IT in terms of tumor tissue pathological response[1] at time of surgery compared to RECIST 1.1 (FDG-PET and perfusion and diffusion weighted MRI).

Secondary endpoints:

- We will monitor immune cell subsets and cytokines in the peripheral blood and tumor[2] compartment.
- Rate and type of late AEs (NCI CTCAE v 4.0) up to 2 years FU after SOC (see Figure 1).
- Relapse free survival (RECIST 1.1) and overall survival at 2 years follow-up.
- The rate of tumor hypoxia before and after immunotherapy[3].
- The oral and gut microbiome (comprising bacteria, archea, viruses and fungi) and the oral metabolome profile in patients with advanced HNSCC before and after anti-cancer immune therapy[4].
- The prevalence of ctDNA before, during and after treatment with immunotherapy and in the event of tumor recurrence[5].
- The transcriptome[5] of all, single cells in patient tumor material before and after two cycles of neoadjuvant immunotherapy and in the event of tumor recurrence.

- The abundance of cDC1 cells in tumor tissue and draining lymph nodes.

[1] Defined as percentage residual tumor cells after neoadjuvant immunotherapy by comparing the tumor tissue biopsies before and after nivolumab w/wo ipilimumab, according to existing guidelines to assess pathological tumor response to neoadjuvant therapy [31], see paragraph 7.3.5). Also, the tumor immune infiltrate will be scored (see paragraph 7.3.5).

[2] Tumor T-cell abundance by IHC, tumor T-cell transcriptome / RNA sequencing after T cell sorting, bulk Tumor IHC and Luminex and RNA sequencing.

[3]Tumor sample hypoxia or normoxia will be further assessed by comparison of RNAseq data on obtained biopsies with validated bulk RNA hypoxia signatures [13], and by tumor HIF1alpha IHC [32]

[4] Obtained via oral swabs and collection of feces before and after treatment with neoadjuvant immunotherapy. Performed and LUMC.

[5] Using Cyclomics-technology of peripheral blood obtained at baseline and after neoadjuvant immunotherapy. Performed in UMCU.

[5] One half of the 8mm tumor core will be sent for single-cell omics at baseline, after immunotherapy and in case of recurrence.

2.7 Risk benefit assessment

Benefit

Advanced primary or recurrent HNSCC is treated with major surgery (among others commando-procedure) with or without (C)RT. Despite the use of surgical free vascular reconstruction flaps –allowing for the reconstruction of large defects and proper resection margins- and despite adding high-dose cisplatin concurrently to adjuvant RT, the overall survival of these patients remained 20-40% 5-year [2, 3]. In addition, no improvement of clinical outcome was established by applying neo-adjuvant chemotherapy regimens [4].

In 2015, with Pembrolizumab a new and promising treatment modality for this type of patients was introduced, as Seiwert found 25% tumor response rate in metastatic HNSCC, in both HPV- and non-HPV related disease [5]. In addition, in recurrent and

metastatic disease, Nivolumab has shown to double the one-year survival rates of HNSCC form 17% to 39% [6].
Meanwhile, it was shown that combining aPD1 and aCTLA4 checkpoint inhibitors leads to superior immunomodulation, resulting in increased durable tumor response rates of 58% and 31% in other carcinomas with a high-mutational load as melanoma and lung carcinoma, respectively [7, 8].

Therefore, participation in this trial may offer these patients the chance for significant improved clinical outcome in terms of loco-regional control and survival, based on the above observations.

Risk

In palliative HNSCC setting, Nivolumab monotherapy 3 mg/kg every 2 weeks for a treatment time of 1.9 months has resulted in 13% grade 3-4 side effects [6]. In a previous trial involving lung carcinoma and the combination treatment of (neoadjuvant) nivolumab 3 mg/kg (median 8 doses, every 2 weeks) and ipilimumab 1 mg/kg (median 2 doses, every 6 weeks) was accompanied by 28% grade 3-4 AEs [8]. As lung carcinoma patients may be relatively comparable to head and neck SCC patients concerning age and smoking status, it could be that the toxicity data of this lung carcinoma trial may reflect the toxicity to be expected in our patient population. Of Note: Our IMCISION trial involves less infusions (two dosages) of immunotherapy and consequently a shorter immunotherapy treatment time (3 weeks), when compared to the above described trials.

Preliminary data of our institute show that our hospital has gained the expertise to manage a nivo/ipi combination scheme in a neoadjuvant setting without delaying the time of surgery [9]. Nevertheless, of caution, the proposed IMCISION treatment cohort 2 will offer the combination of nivo/ipi (nivo 3 mg/kg and ipi 1mg/kg) once in the first week, and it will offer nivo monotherapy (3 mg/kg) in week 3, whereas the OPACIN trial offered combined nivo/ipi (3 and 3 mg/kg) in both weeks 1 and 4. As in our proposed study the combination of nivolumab and ipilimumab will be given 4 weeks prior to surgery, and as the last infusion of nivolumab will be given two weeks prior to surgery, patients will be enabled to recover from acute side effects.

In summary, we believe that the expected grade 3-4 toxicity in the proposed IMCISION trial will be manageable and will not delay the time of surgery.

For this trial, patients will undergo 2-3 extra tumor biopsies for research purposes twice: the first time during routine investigation under general anesthesia, and the second time during routine surgery. The first time, it is our experience that patients may endure slight temporarily discomfort due taking extra biopsies and a very small enlarged risk (< 1%) for bleeding and infection afterwards. The second time, patients will obviously not experience any side-effects from harvesting these biopsies, as the tumor resection specimen will be dissected during surgery.
Patients will undergo twice 5x2 oral mucosal swabs before and after immune therapy (see patients and methods) for microbiome and metabolite research purposes. The first time during routine investigation under general anesthesia, and the second time during routine surgery. This procedure is safe and patients will not experience any burden as they are under anesthesia while taking the swabs. Patients will collect stool at home using a stool collection kit and hand in the sample during their routine hospital visit. This procedure is considered simple and non-invasive.

3. INTRODUCTION AND RATIONALE

3.1 Current standard treatment

Currently, primary surgery with or without adjuvant (chemo)radiation is the standard in advanced stage HNSCC of the oral cavity and for salvage after failed primary treatment. Patients generally suffer mutilating surgery, and nevertheless have a very poor prognosis of 37% 5-year overall survival in stage IV oral HNSCC [2] and 20-40% 2-year overall survival after salvage surgery [3].

In our clinic, indication for postoperative chemoradiation (cisplatin) is indisputable if incomplete resection and/or extracapsular growth. Radiation without concurrent chemotherapy is indicated in case of pT4 disease. Adjuvant radiation is also indicated in case of 2 or more of the following criteria: pT3 disease, infiltrative growth, close margins (1-5 mm), perineural growth, lymphovascular invasion and infiltration depth >10mm. Based on lymph node involvement, adjuvant radiation is indicated if ≥ pN2b status.

3.2 Neoadjuvant nivolumab and ipilimumab: A risk benefit assessment

Benefit

Currently, primary surgery with or without adjuvant (chemo)radiation is the standard in advanced stage HNSCC of the oral cavity and for salvage after failed primary treatment. Patients generally suffer mutilating surgery, and nevertheless have a very poor prognosis of 37% 5-year overall survival in stage IV oral HNSCC [2] and 20-40% 2-year overall survival after salvage surgery [3]. The introduction of free pedicle transplantation flaps to reconstruct and allow for larger surgical defects and therefore wider surgical margins, did not lead to an improvement in clinical outcome over the last decades. To date, although multiple (neo)adjuvant chemotherapeutic regimens have been evaluated, clinical benefit fails to appear [4].

T cell checkpoint blockade by anti-CTLA and/or anti-PD1 is currently the most promising in immunomodulation anticancer therapies. Recently, published by others, pembrolizumab (PD1 blockade) given at a fixed dose of 200 mg every 3 weeks was well tolerated and demonstrated a clinically meaningful overall response rate (ORR) of 25% in patients with recurrent/metastatic HNSCC, irrespective of HPV status [5].

In addition, Nivolumab monotherapy, given biweekly at a dosage of 3 mg/kg, for approximately 2 months, has resulted in doubling of 1 year survival rates from 17% to 35% of a large HNSCC patient cohort in palliative setting [6].

The rationale behind combining aPD1 and aCTLA4 is that nivolumab and ipilimumab enhance T-cell antitumor activity through distinct but complementary mechanisms resulting in both enhanced T-cell priming and enhanced local T-cell-mediated tumor regression. The complementary effect of both checkpoint inhibitors was first proven in a phase III trial treating metastatic melanoma resulting in significant durable response rates of 58% [7]. Shortly thereafter, a lung carcinoma trial was performed, concerning 39 patients with stage IIIB/IV NSCLC treated with nivolumab 3 mgr/kg and ipilimumab 1 mgr/kg, 8 and 2 infusions respectively and overall treatment period of 15-18 weeks, resulting in a 31% durable partial response rate [8].

Moreover, very recently, it was postulated that offering the nivolumab and ipilimumab in neo-adjuvant setting would exert even stronger immunomodulation and increased tumor responses to treatment, when compared to adjuvant immunotherapy. The rationale is amongst others that the presence of tumor load before surgery offers increased neo-antigen presentation with consequently more efficient T-cell receptor triggering. In addition, reduced tumor heterogeneity, as compared to treatment in the metastatic setting, and improved immune status during earlier disease status are factors that are likely to positively influence the efficacy of immunomodulation in this setting. Indeed, recent preliminary clinical data provide support for this hypothesis [9].

Therefore, participation in this trial, may offer our patients the chance for a significant improved clinical outcome in terms of loco-regional control and survival, based on the above observations.

Risk

Pembrolizumab (aPD-L1) monotherapy in recurrent and metastatic setting in HNSCC is followed by drug-related AEs grade ≥ 3 AEs of 9.8%, of which pneumonitis (2) and face swelling (2) were most common. [5]. Nivolumab monotherapy 3 mg/kg every 2 weeks for a treatment time of 1.9 months in a palliative setting for HNSCC has resulted in 13% grade 3-4 side effects mostly fatigue and anemia [6]. In a previous

trial involving lung carcinoma and the combination treatment of (neoadjuvant) nivolumab 3 mg/kg (median 8 doses, every 2 weeks) and ipilimumab 1 mg/kg (median 2 doses, every 6 weeks) was accompanied by 28% grade 3-4 AEs, more specifically endocrine (5%), gastrointestinal (5%), hepatic (5%), pneumonitis (3%), and skin (5%) [8]. As lung carcinoma patients may be relatively comparable to head and neck SCC patients concerning age and smoking status, it could be that the toxicity data of this lung carcinoma trial may reflect the toxicity to be expected in our patient population. Of Note: Our IMCISION trial involves less infusions (two dosages) of immunotherapy and consequently a shorter immunotherapy treatment time (3 weeks), when compared to the above described trials.

Preliminary data of our institute show that our hospital has gained the expertise to manage a nivo/ipi combination scheme in a neoadjuvant setting without delaying the time of surgery [9]. Nevertheless, of caution, the proposed IMCISION treatment cohort 2 will offer the combination of nivo/ipi (nivo 240 mg and ipi 1mg/kg) once in the first week, and it will offer nivo monotherapy (240 mg) in week 3, whereas the OPACIN trial offered combined nivo/ipi (3 and 3 mg/kg) in both weeks 1 and 4. In addition, as in our proposed study the combination of nivolumab and ipilimumab will be given 4 weeks prior to surgery, and as the last infusion of nivolumab will be given two weeks prior to surgery, patients will be enabled to recover from acute side effects.

In summary, nivolumab and ipilimumab coincide with manageable transient and mostly-immune related toxicity. In view of the above observations, we believe that the expected grade 3-4 toxicity in the proposed IMCISION trial will be manageable and will not delay the time of surgery.

Surgery will be planned on the first day in week 5 (day 29), in agreement with the Dutch Guideline for treating HNC. The radiation burden for an extra FDG PET/CT is estimated at 5 mSv for 200 MBq FDG + 3 mSv for low dose CT = 8 mSv. These doses are in the range of normal diagnostic procedures, are in risk category IIIa (justified research in normal heathy adults for prevention or cure of diseases in the future) of the Nederlandse commissie voor stralingsdosimetrie, and the additional risk

is considered irrelevant in the cohort of patients with a malignant tumor in the head-neck area.
When taken biopsies the first time, it is our experience that patients may endure slight temporarily discomfort due taking extra biopsies and a very small enlarged risk for bleeding afterwards (see also paragraph 7). The second time, patients will obviously not experience any side-effects from harvesting these biopsies, as the tumor resection specimen will be dissected during surgery.

3.3 Hypoxia as a potential biomarker in immunotherapy

Hypoxia is a common feature of solid tumors [33] related to poor prognosis in malignant disease including NSCLC and HNSCC. Hypoxia clearly uniformly causes radio-resistance and RT strategies targeting hypoxia are among the few that succeeded in improving locoregional control [34, 35]. In addition, hypoxic cells are less sensitive to cytotoxic agents, on the one hand through lower drug concentrations in hypoxic areas (diffusion limitation) and on the other hand because hypoxic cells proliferate more slowly and are therefore more resistant to proliferation-dependent drugs. Finally, in case of surgical resection, tumor hypoxia is correlated with distant failure after loco-regional treatment [36], while hypoxia has been shown to promote cancer invasion and metastasis formation [34].

Although impressive improvement in overall survival has been found with immune checkpoint inhibitors, the effects of variation in the tumor micro-environment on clinical response to therapy are not yet fully understood, with a corresponding lack of an available clinical biomarker for individual treatment response. With this research we would like to explore the relationship between clinically observed hypoxia on the effect of nivolumab/ nivolumab + ipilimumab on T cell function in HNSCC. The working model we aim to test is that hypoxia may limit the positive effect of check-point blockade on T cell function.

Extensive preclinical data support impact of hypoxia on T cell capacity. Hypoxia causes increased formation of the HIF1 heterodimer, which enters the nucleus of the cell to target hypoxia response elements (HRE) and genes for altering the transcriptome towards anaerobic metabolism needed for cell survival [37]. More specifically, one effect of hypoxia is stabilization of the alpha subunit of the hypoxia-

inducible factor (HIF-)1α transcription factor. HIF-1α-stabilization increases transcription of proteins that improve cellular survival under these hypoxic circumstances through various mechanisms [38]. Glucose influx is increased by upregulating glucose transporter (GLUT)1 and intracellular acidosis is prevented by upregulation of carbonic anhydrase IX (CA-IX). Moreover, angiogenesis is stimulated by increased expression of the vascular endothelial growth factor (VEGF). Overall, increased HIF1α results in an increase in glycolysis and reduced oxidative phosphorylation, even in the presence of oxygen, a phenomenon known as the Warburg effect.

Several researchers have studied expression of HIF-1α, GLUT-1 or CA-IX in relation to survival in HNSCC and found that overexpression of these proteins was associated with decreased survival [39, 40]. Furthermore, robust RNA expression hypoxia signatures have been developed over the years. Such signatures are used to identify patients for selective treatment to overcome hypoxia such as RT with Nimorazole [13, 41].

While tumor hypoxia is correlated to clinical cancer progression and clinical cancer treatment resistance as described above, recently, several mechanisms of hypoxia-mediated modulation of cancer immune interactions were discussed [42-44]. First, exposure of human cancer cells to hypoxia led to increased expression levels of important molecular targets such as PD-L1. Second, hypoxic zones in tumors attract immunosuppressive cells such as myeloid-derived suppressor cells (MDSCs), macrophages, and regulatory T cells [43, 45]. The authors indicated that hypoxia selectively upregulated PD-L1 via HIF-1α (but not HIF-2α) in tumor cells and immune cells themselves such as MDSCs, macrophages and dendritic cells, an observation that may suggest that PD-1 blockade is of particular importance at such sites. On the other hand, the increased presence of MDSC and Tregs may also interfere with T cell function through processes that are entirely independent of PD-1 – PD-L1 interactions, thereby potentially negating the positive effect of nivolumab in these areas. Finally, the direct effect of intra-tumor hypoxia on T cell function is difficult to predict, as during transition towards the effector cell stage, T cells are programmed to switch to anaerobic glycolysis, making it uncertain how encounter of a hypoxic state would further influence them. Below, we briefly describe the various data as obtained in prior preclinical in vitro and in vivo studies concerning this latter topic.

Hypoxia-inducible factors regulate T cell metabolism and function [37]. Naive T cells depend on oxidative phosphorylation (OXPHOS) providing ATP for the transition to an activated T cell state [46]. In contrast, activated effector T cells depend on anaerobic glycolysis (Warburg effect) for proliferation [47] and the production of effector cytokines as IL2 and IFNɣ [46]. The memory T cells that remain upon clearance of antigen again depend on OXPHOS, fatty acid metabolism and increased mitochondrial mass for capacity in case of reinfection [47]. Thus, changes in lymphocyte metabolism accompany key cell-fate decisions and, vice versa, T cell differentiation and effector function may be dependent on increased HIF1α and/or hypoxia. Moreover, in vitro, both CD4+ and CD8+ T cells switch towards glycolysis, however CD4+ T cells retain a proportionally stronger oxidative metabolism [48]. Consequently, the effect of hypoxia on T cell metabolism may differ per T cell subset.

The effect of hypoxia and/or increased HIF1 in T cells has been addressed in previous preclinical studies with potentially conflicting outcome (figure 2).

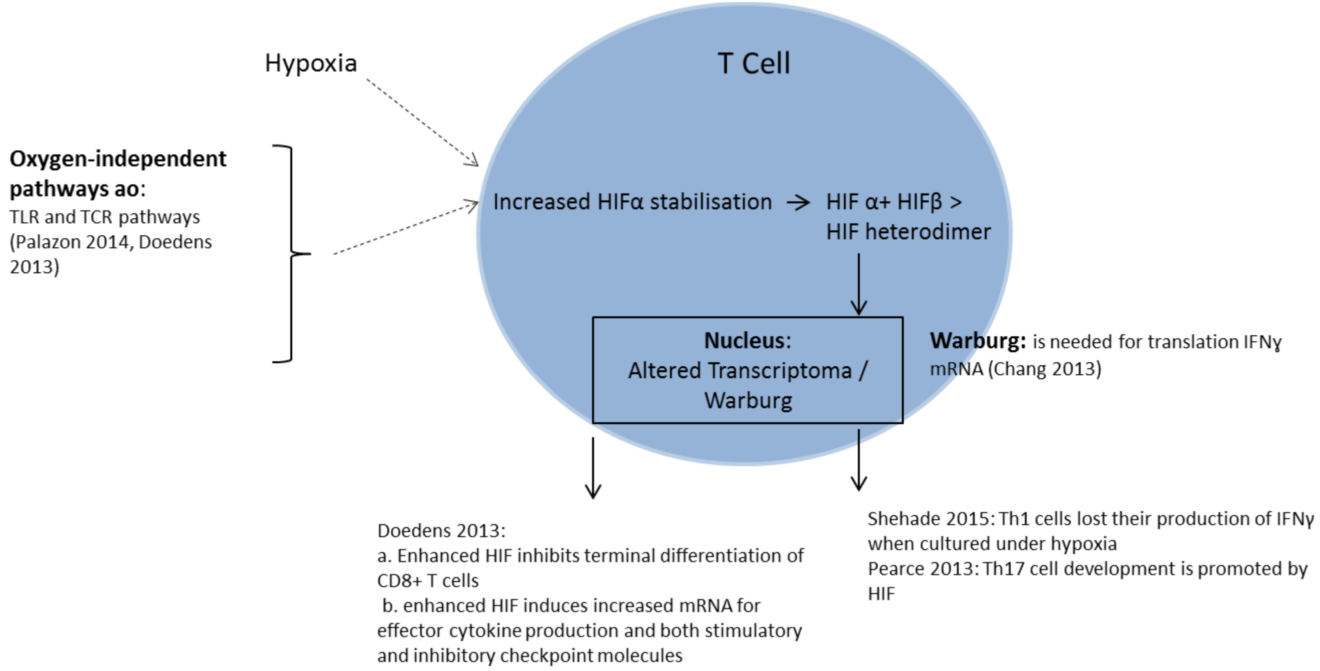

Figure 2: the effect of hypoxia in T cells; data collected from various preclinical studies

As expected, VHL deficient CD8+ T cells showed upregulation of mRNA encoding molecules for glycolytic metabolism and a lower oxygen consumption rate [49]. However, while Doedens found an increase in mRNA for effector molecules (granzyme B, TNF), Th1 cells lost their capacity to produce IFNɣ when cultured under hypoxia in a HIF1α dependent manner in another study [50]. In addition, Doedens et al. found an upregulation of co-stimulatory receptors GITR and OX40 in CTLs, however also inhibitory receptors LAG-3 and CTLA4. Moreover, enhanced HIF activity seems to inhibit or delay differentiation of CD8+ effector T cells in mice [49], while HIF1α was crucial for proper Th17 development in another study [47, 51]. Hence, the net effect of hypoxia and/or HIF1 α stabilization on T cell capacity in human tumors remains unclear and may differ per T cell subset.
Interestingly, in in vitro assays, altered metabolism influences translation of mRNA of T cell effector proteins (rather than transcription) [46]. Research aiming at analysis of the effect of hypoxia on intratumoral T cell capacity should therefore also include cytokine assays.
Finally, HIF stabilization is increased by TLR and TCR signaling pathways in an O_2-independent manner in both myeloid and T cells [44]. Hence, it is of additional interest to investigate whether or to what extent hypoxia in tumors does re-program T cells or not.

In summary, hypoxia is a well-known feature of various solid tumors and a biomarker for unfavorable cancer treatment response. A vast amount of preclinical data suggests a potential key role for hypoxia in tumor response to immunotherapy. However, the net effect of hypoxia on TME and more specifically on T cell function in human solid cancer after PD-1 blockade is currently unknown. As such, the results of our study may have implications for the development of combination treatments that aim to further enhance tumor-specific T cell activity in various solid tumors during checkpoint blockade therapy.

3.4 Dysbiosis as a biomarker for anti-cancer treatment

Another potential biomarker can be found in the human microbiota. Microbial imbalance (dybiosis) on or inside the body may affect oncogenesis, tumor progression and response to cancer therapy [15, 16]. Microbes present at mucosal

sites may exert different immune-modulatory effects. Segmented filamentous bacteria, which can breach the gut mucus layer and attach to intestinal epithelial cells (IECs) are a potent inducer of T helper 17 cells and for instance Helicobacter Pylori may lead to an increase in loco-regional T-regs [17, 18]. During cancer therapy, radiation or drug regimens may exert toxic effects on bacteria, thereby promoting dysbiosis. On the other hand, (gut) microbiota influences the therapeutic efficacy and side effects of cancer drugs via pharmacodynamics and immunological mechanisms [15, 16]. Indeed, recent work has shown that gut microbiota interfere with tumor responses to chemotherapy and aPD1 or aCTLA4 immunotherapy [17, 19, 20]. In HNSCC, the oral cavity harbors more than 700 bacterial species and is one of the most densely populated areas of the human body. Differences in bacterial- and viral composition have been described for oral cancers in patient series [21], potentially serving as biomarker for oral cancer. The microbiome, its metabolome, and its potential effect on cancer treatment in HNSCC was never studied before. For translational research purposes.

The human microbiota is defined as the collective of microorganisms that reside in the human body or its surfaces. The “microbiome” is reserved for the genomes of this collective group of microorganisms. Microbial imbalance (dybiosis) on or inside the body may affect oncogenesis, tumor progression and response to cancer therapy [15, 16]. First of all, risk factors for dysbiosis such as antibiotics, smoking, diet and alcohol consumption, may also promote carcinogenesis directly. Secondly, an altered gut microbiota may affect the incidence and progression of both locoregional carcinogenesis and extra-intestinal cancers [15]. Of interest, microbes present at mucosal sites may exert different immune-modulatory effects. Segmented filamentous bacteria, which can breach the gut mucus layer and attach to intestinal epithelial cells (IECs) are a potent inducer of T helper 17 cells and for instance Helicobacter Pylori may lead to an increase in loco-regional T-regs [17, 18].

During cancer therapy, radiation or drug regimens may exert toxic effects on bacteria, thereby promoting dysbiosis. On the other hand, (gut) microbiota influences the therapeutic efficacy and side effects of cancer drugs via pharmacodynamics and immunological mechanisms [15, 16]. Indeed, recent work has shown that gut

microbiota interfere with tumor responses to chemotherapy and aPD1 or aCTLA4 immunotherapy [17, 19, 20].

In HNSCC, the oral cavity harbors more than 700 bacterial species and is one of the most densely populated areas of the human body. The oral cavity the largest core of commonly shared microbes among unrelated individual and provides an ideal source for biomarker discoveries due to low inter- and intra- biological variations. The oral cavity includes several distinct habitats as the tooth surface/gingival crevice, tongue, tonsils and oropharynx each hosting a distinct microbiota [52]. Differences in bacterial- and viral composition have been described for oral cancers in patient series [21], potentially serving as biomarker for oral cancer. The microbiome, its metabolome, and its potential effect on cancer treatment in HNSCC was never studied before.

With this study we aim to assess the oral and gut microbiome (comprising bacteria, archea, viruses and fungi) and the oral metabolome profile in patients with advanced HNSCC before and after anti-cancer immune therapy. Data will be analyzed in view of the other primary and secondary endpoints of the N16IMC trial: (single cell) intra-tumor T cell function at steady state (before IT) and after immunotherapy, and pathological-, MRI and PET tumor response to immunotherapy.

3.5 Measuring immunotherapy response in liquid biopsies

Levels of circulating tumor DNA (ctDNA) in ‘liquid biopsies’ (e.g. blood, saliva) could serve as a novel, non-invasive biomarker for determining response to treatment [22-25]. 80-90% of non-HPV HNSCC patiënts carry TP53 mutations, which can be detected in ctDNA [26, 27]. Therefore, screening for changes in the levels of TP53 mutations in the blood of HNSCC-patients which receive immunotherapy may provide an ideal biomarker for predicting treatment response [28]. Low levels of TP53 mutations can be reliably detected among the other cell-free DNA using a new technique, Cyclomics, which is based on Oxford Nanopore MinION sequencing of concatenated copies of a single DNA molecule. We will use this technique to determine the levels of TP53 mutations in the blood of 20 stage III-IV HNSCC patients with a TP53 mutation both prior to and after immunotherapy. By comparing

these TP53 mutation levels with e.g. MRI scans, we aim to dissect whether a liquid biopsy provides a more accurate (and less invasive) prediction for immunotherapy response.

3.6 Single cell sequencing of matched patient samples

Whereas bulk sequencing efforts lack the depth and sensitivity to detect disease mechanisms in heterogenous cell populations, single-cell RNA sequencing is able to identify transcriptional changes on a single-cell basis. This provides insight into how cells interact, and may identify previously overlooked cell types involved in disease development, disease progression and the interaction with the immune system, including in HNSCC patients [29]. In addition, single-cell sequencing of matched patient samples before and after neoadjuvant immunotherapy may efficiently identify responding cell populations and, on a larger scale, patients who are more likely to benefit from immunotherapy [30]. This might provide a rationale for future trial and drug design. We will use single-cell sequencing technology to analyze our patient's tumor samples at baseline, after immunotherapy and in the case of tumor recurrence.

3.7 Dendritic cells in tumor-draining lymph nodes

The role of Type 1 conventional dendritic cells (cDC1) in tumor-draining lymph node (tdLN) is to cross-present tumor antigens and prime anti-tumor CTL response, and in the tumor microenvironment (TME) is to re-stimulate tumor infiltrating T cells and help to sustain the tumor-specific cytotoxic T lymphocyte (CTL) response in tumor site locally, which explains why the abundance of cDC1s in TME is positively correlates with the responsiveness to anti–PD-1 therapy and results in a better survival of cancer patients (Böttcher *et al.* The Role of Type 1 Conventional Dendritic Cells in Cancer Immunity. *Trends in Cancer* 2018). Our preliminary study indicates that the human cDC1 is the most capable DC subset in relaying the “help” signal from the $CD4^+$ T cell to generate a tumor-specific CTL response (Xiao *et al.* unpublished data). Therefore in addition of monitoring tumor infiltrating CD8 T cells, macrophages and MDSCs (as described in 7.2.2 of the IMCISION protocal), the presence of cDC1 and activated CD4 T cells and their phenotype in tdLN and TME also need to be measured. This information could be an indicator for identifying the responder

patients. We propose to obtain biopsies of tdLN, non tdLN (control samples) and tumor tissues (size = 4mm). All biopsy samples will be cryopreserved according to the procedure of viable lymph node and tumor tissue preparation until final use.

4. STUDY ENDPOINTS

Primary endpoints:

- Phase Ib: Primary endpoint is measured as the number of patients that will not endure a delay in surgery (surgery should be performed in week 5-6) due to neoadjuvant immunotherapy (nivolumab, ipilimumab) related toxicity (measured in terms of SAEs and CTCAE v4.0) OR the treatment of immunotherapy related toxicity (ie high dose corticosteroids)**.
  ** To meet this endpoint, all patients will be discussed in our immunotherapy team meeting (consisting of at least medical oncologist and head and neck surgeon) the week before surgery, to evaluate whether immunotherapy-related toxicity or treatment of immunotherapy-related toxicity will lead to delay in surgery or not.
  ** Delay in surgery due to logistical problems (i.e. no IC bed after surgery) or other co-morbidity (i.e. bacterial pneumonia) will not be considered dose-limiting toxicity.
- Phase II: Tumor response to neoadjuvant IT in terms of tumor tissue pathological response[1] at time of surgery compared to RECIST 1.1 (FDG-PET and perfusion and diffusion weighted MRI).

Secondary endpoints:

- We will monitor immune cell subsets and cytokines in the peripheral blood and tumor[2] compartment.
- Rate and type of late AEs (NCI CTCAE v 4.0) up to 2 years FU after SOC (see Figure 1).
- Relapse free survival (RECIST 1.1) and overall survival at 2 years follow-up.
- The rate of tumor hypoxia before and after immunotherapy[3].
- The oral and gut microbiome (comprising bacteria, archea, viruses and fungi) and the oral metabolome profile in patients with advanced HNSCC before and after anti-cancer immune therapy[4].
- The prevalence of ctDNA before, during and after treatment with immunotherapy and in the event of tumor recurrence[5].
- The transcriptome[5] of all, single cells in patient tumor material before and after two cycles of neoadjuvant immunotherapy and in the event of tumor recurrence.

- The abundance of cDC1 cells in tumor tissue, tumor-draining lymph nodes and non-tumor draining lymph nodes.

[1] Defined as percentage residual tumor cells after neoadjuvant immunotherapy by comparing the tumor tissue biopsies before and after nivolumab w/wo ipilimumab, according to existing guidelines to assess pathological tumor response to neoadjuvant therapy [31], see paragraph 7.3.5). Also, the tumor immune infiltrate will be scored (see paragraph 7.3.5).

[2] Tumor T-cell abundance by IHC, tumor T-cell transcriptome / RNA sequencing after T cell sorting, bulk Tumor IHC and Luminex and RNA sequencing.

[3]Tumor sample hypoxia or normoxia will be further assessed by comparison of RNAseq data on obtained biopsies with validated bulk RNA hypoxia signatures [13], and by tumor HIF1alpha IHC [32]

[4] Obtained via oral swabs and collection of feces before and after treatment with neoadjuvant immunotherapy. Performed and LUMC.

[5] Using Cyclomics-technology of peripheral blood obtained at baseline and after neoadjuvant immunotherapy. Performed in UMCU.

[5] Using Celsius Inc.'s single-cell sequencing platform. Performed in Cambridge, MA, USA. One 8mm tumor core will be halved, with one half sent for single-cell omics at baseline, after immunotherapy and in case of recurrence.

5. STUDY POPULATION

5.1 Population (base)

Thirty-two patients fit for curative surgery for extensive (recurrent) HNSCC, naïve for immunotherapy, more than 18 years old will be eligible for this trial.

The scientific advantage of employing this specific patient population is that mucosal carcinomas of the oral cavity and oropharynx are relatively easily exposed, allowing for multiple tumor biopsies prior to neoadjuvant immunotherapy (during routine diagnostic investigation under general anesthesia) and again after neoadjuvant immunotherapy (during curative surgery).

5.2 Inclusion criteria

In order to be eligible to participate in this study, a subject must meet all of the following eligibility criteria:

- Adults age 18 years and older
- Patients with histologically confirmed T2-4N0-3M0 HNSCC of the oral cavity, oropharynx, hypopharynx or larynx, eligible for major curative surgery (e.g. COMMANDO-procedure, laryngectomy) as primary treatment or major salvage surgery after failed (chemo)radiation.
- Performance Status ECOG 0 or 1, e.g.:
    - 0 – Asymptomatic (fully active, able to carry on all predisease activities without restriction).
    - 1 – Symptomatic but completely ambulatory (restricted in physically strenuous activity but ambulatory and able to carry out work of a light or sedentary nature).
- No immunosuppressive medications within 6 months prior study inclusion
- Screening laboratory values must meet the following criteria: WBC ≥ 2.0×10^9/L, Neutrophils ≥ 1.5×10^9/L, Platelets ≥ 100 $\times 10^9$/L, Hemoglobin ≥ 5.5 mmol/L, Creatinine ≤ 1.5x ULN, AST ≤ 3 x ULN, ALT ≤ 3 x ULN, Total Bilirubin ≤1.5 X ULN (except subjects with Gilbert Syndrome, who can have total bilirubin < 3.0 mg/dL)
- No hard-drug abuse.

- Women of childbearing potential (WOCBP) must use appropriate method(s) of contraception. WOCBP should use an adequate method to avoid pregnancy for 23 weeks (30 days plus the time required for nivolumab to undergo five half-lives after the last dose of investigational drug.
- Women of childbearing potential must have a negative serum or urine pregnancy test (minimum sensitivity 25 IU/L or equivalent units of HCG) within 24 hours prior to the start of nivolumab w/wo ipilimumab.
- Men who are sexually active with WOCBP must use any contraceptive method with a failure rate of less than 1% per year Men receiving nivolumab w/wo ipilimumab and who are sexually active with WOCBP will be instructed to adhere to contraception for a period of 31 weeks after the last dose of investigational product.
- Women who are not of childbearing potential (i.e., who are postmenopausal or surgically sterile) as well as azoospermic men do not require contraception.
- All subjects must have signed and dated the written informed consent.

5.3 Exclusion criteria

A potential subject who meets any of the following criteria will be excluded from participation in this study:

- Distant metastasis
- Active, known or suspected autoimmune disease. Subjects are permitted to enroll if they have vitiligo, type I diabetes mellitus, residual hypothyroidism due to autoimmune condition only requiring hormone replacement, psoriasis not requiring systemic treatment, or conditions not expected to recur in the absence of an external trigger.
- Patients should be excluded if they have a condition requiring systemic treatment with either corticosteroids (> 10 mg daily prednisone equivalents) or other immunosuppressive medications within 14 days of study drug administration. Inhaled or topical corticosteroids and adrenal replacement doses > 10 mg daily prednisone equivalents are permitted in the absence of active autoimmune disease.

- Prior systemic treatment with an anti-PD-1, anti-PD-L1, anti-PD-L2, anti-CTLA-4 antibody, or any other antibody or drug specifically targeting T-cell costimulation or immune checkpoint pathways;
- Patients will be excluded if they are positive test for hepatitis B virus surface antigen (HBV sAg) or hepatitis C virus ribonucleic acid (HCV antibody) indicating acute or chronic infection;
- Patients will be excluded if they have known history of testing positive for human immunodeficiency virus (HIV) or known acquired immunodeficiency syndrome (AIDS);
- Allergies and Adverse Drug Reaction: history of allergy to study drug components, history of severe hypersensitivity reaction to any monoclonal antibody.
- Underlying medical conditions that, in the Investigator's opinion, will make the administration of study drug hazardous or obscure the interpretation of toxicity determination or adverse events;
- Concurrent medical condition requiring the use of immunosuppressive medications, or immunosuppressive doses of systemic or absorbable topical corticosteroids;
- Use of other investigational drugs before study drug administration 30 days and 5 half times before study inclusion;
- Pregnant or nursing.
- As there is potential for hepatic toxicity with nivolumab or nivolumab/ipilimumab combinations, drugs with a predisposition to hepatotoxicity should be used with caution in patients treated with nivolumab-containing regimen.
- In those patients who have a contraindication for cisplatin chemotherapy, Cetuximab might be used as a radiosensitizer for radiotherapy if adjuvant treatment is deemed necessary.

6. INVESTIGATIONAL PRODUCT

6.1 Name and description of investigational product(s)

Cohort 1: nivolumab 240 mg, 2 times, week 1 and 3.

Cohort 2: nivolumab 240 mg and ipilimumab 1 mg/kg, 1 time, week 1, AND nivolumab 240 mg, 1 time, week 3.

**Table**	**Product Description**				
**Product Description and Dosage Form**	**Potency**	**Primary Packaging (Volume)/ Label Type**	**Secondary Packaging (Qty) /Label Type**	**Appearance**	**Storage Conditions (per label)**
Nivolumab BMS-936558-01 Solution for Injection[a]	100 mg (10 mg/mL)	10 mL viall	5-10 vials per carton/ Open-label	Clear to opalescent colorless to pale yellow liquid. May contain particles	2 to 8□C. Protect form light and freezing
Ipilimumab Solution for Injection	200 mg (5 mg/mL)	40 mL vial	4 vials per carton/Open-label	Clear, colorless to pale yellow liquid. May contain particles	2 to 8°C. Protect from light and freezing.

*Nivolumab may be labeled as BMS-936558-01 Solution for Injection

6.2 Description and justification of route of administration and dosage

Both ipilimumab and nivolumab are monoclonal antibodies and need to be i.v. infused.

6.3 Dosages, dosage modifications and method of administration

Nivolumab (240 mg) is to be administered as an approximate 30 minute IV infusion, using a volumetric pump with a 0.2/0.22 micron in-line filter at the protocol-specified doses. It is not to be administered as an IV push or bolus injection. At the end of the infusion, flush the line with a sufficient quantity of normal saline (per institutional

standard of care). Details regarding the mixing and concentrations of the dose (preparation) and administration will be found in the current nivolumab IB.

Ipilimumab (1 mg/kg) is to be administered as an approximate 30-minute IV infusion, using a volumetric pump with a 0.2 to 1.2 micron in-line filter at the protocol-specified dose. Care must be taken to assure sterility of the prepared solutions, since the drug product does not contain any antimicrobial preservatives or bacteriostatic agents. See ipilimumab IB for further details regarding preparation / administration. Ipilimumab and nivolumab may be diluted in 0.9% Sodium Chloride Solution or 5% Dextrose solution.

The dosing calculations should be based on the body weight. If the subject's weight on the day of dosing differs by > 10% from the weight used to calculate the dose, the dose must be recalculated. All doses should be rounded up or to the nearest milligram per institutional standard.

Both study drugs are to be administered on the same day in this study. Separate infusion bags and filters must be used for each infusion. Nivolumab is to be always administered first. The nivolumab infusion must be promptly followed by a saline flush to clear the line of nivolumab before starting the ipilimumab infusion. The second infusion will always be the ipilimumab study drug, and will start no sooner than 30 minutes after completion of the nivolumab infusion.

Following the first doses of the combination of nivolumab and ipilimumab, the second dose of nivolumab will be given no less than 12 days from the previous dose; and dosed up to 3 days after the scheduled date if necessary.

Dose reductions or dose escalations are not permitted.

6.4 Dose Delay Criteria

Because of the potential for clinically meaningful nivolumab-related AEs requiring early recognition and prompt intervention, management algorithms have been developed for suspected AEs of selected categories (see appendices).

Dose delay criteria apply for all drug-related adverse events (regardless of whether or not the event is attributed to nivolumab, ipilimumab or both). All study drugs must be delayed until treatment can resume.

Dose delay may not interfere with the timing of surgery.

Nivolumab and ipilimumab administration should be delayed for the following:

- Any Grade ≥ 2 non-skin, drug-related adverse event, with the following exceptions:
    - Grade 2 drug-related fatigue or laboratory abnormalities do not require a treatment delay
- Any Grade 3 skin, drug-related adverse event
- Any Grade 3 drug-related laboratory abnormality, with the following exceptions for asymptomatic amylase or lipase, AST, ALT, or total bilirubin:
    - Grade 3 amylase or lipase abnormalities that are not associated with symptoms or clinical manifestations of pancreatitis do not require a dose delay. It is recommended to consult with the principle investigator for Grade 3 amylase or lipase abnormalities.
    - If a subject has a baseline AST, ALT, or total bilirubin that is within normal limits, delay dosing for drug-related Grade ≥ 2 toxicity
    - If a subject has baseline AST, ALT, or total bilirubin within the Grade 1 toxicity range, delay dosing for drug-related Grade ≥ 3 toxicity
- Any adverse event, laboratory abnormality, or intercurrent illness, which in the judgment of the investigator, warrants delaying the dose of study medication.

For subjects expected who require more than 4 weeks of corticosteroids or other immunosuppressants to manage an adverse event, the following recommendations should be considered:

- Antimicrobial/antifungal prophylaxis per institutional guidelines to prevent opportunistic infections such as Pneumocystis jiroveci and fungal infections;

- Early consultation with an infectious disease specialist. Depending on the presentation, consultation with a pulmonologist for bronchoscopy or a gastroenterologist for endoscopy may also be appropriate.
- In patients who develop recurrent adverse events in the setting of ongoing or prior immunosuppressant use, an opportunistic infection should be considered in the differential diagnosis.

6.5 Criteria to Resume Treatment

Subjects may resume treatment with study drug when the drug-related AE(s) resolve to Grade ≤1 or baseline value, with the following exceptions:

- Subjects may resume treatment in the presence of Grade 2 fatigue
- Subjects who have not experienced a Grade 3 drug-related skin AE may resume treatment in the presence of Grade 2 skin toxicity
- Subjects with baseline Grade 1 AST/ALT or total bilirubin who require dose delays for reasons other than a 2-grade shift in AST/ALT or total bilirubin may resume treatment in the presence of Grade 2 AST/ALT OR total bilirubin
- Subjects with combined Grade 2 AST/ALT AND total bilirubin values meeting discontinuation parameters (Section 6.6) should have treatment permanently discontinued
- Drug-related pulmonary toxicity, diarrhea, or colitis, must have resolved to baseline before treatment is resumed
- Drug-related endocrinopathies adequately controlled with only physiologic hormone replacement may resume treatment

If the criteria to resume treatment are met, the subject should restart treatment at the next scheduled time-point per protocol. However, if neoadjuvant IT is delayed past week 3, the neoadjuvant immunotherapy will be stopped to enable surgery at week 5, as dose delay may not interfere with timing of surgery.

6.6 Discontinuation Criteria

Neoadjuvant immunotherapy should be permanently discontinued for the following:

- In case of tumor progression during neoadjuvant immunotherapy potentially leading to clinically inoperable disease, surgery will be expedited, in which case the second infusion of nivolumab (week 3) may be dropped.
- Any Grade 2 drug-related uveitis or eye pain or blurred vision that does not respond to topical therapy and does not improve to Grade 1 severity within the re-treatment period OR requires systemic treatment
- Any Grade 3 non-skin, drug-related adverse event lasting > 7 days, with the following exceptions for drug-related laboratory abnormalities, uveitis, pneumonitis, bronchospasm, diarrhea, colitis, neurologic adverse event, hypersensitivity reactions, and infusion reactions
    - Grade 3 drug-related uveitis, pneumonitis, bronchospasm, diarrhea, colitis, neurologic adverse event, hypersensitivity reaction, or infusion reaction of any duration requires discontinuation
    - Grade 3 drug-related laboratory abnormalities do not require treatment discontinuation except those noted below
- Grade 3 drug-related thrombocytopenia > 7 days or associated with bleeding requires discontinuation
- Any drug-related liver function test (LFT) abnormality that meets the following criteria require discontinuation:
    - AST or ALT > 8 x ULN
    - Total bilirubin > 5 x ULN
    - Concurrent AST or ALT > 3 x ULN and total bilirubin > 2 x ULN
- Any Grade 4 drug-related adverse event or laboratory abnormality, except for the following events which do not require discontinuation:
- Isolated Grade 4 amylase or lipase abnormalities that are not associated with symptoms or clinical manifestations of pancreatitis and decrease to < Grade 4 within 1 week of onset.

- Isolated Grade 4 electrolyte imbalances/abnormalities that are not associated with clinical sequelae and are corrected with supplementation/appropriate management within 72 hours of their onset
- If neoadjuvant IT is delayed past week 3, the neoadjuvant immunotherapy will be stopped to enable surgery intended at week 5, as dose delay may not interfere with timing of surgery.
- Any adverse event, laboratory abnormality, or intercurrent illness which, in the judgment of the Investigator, presents a substantial clinical risk to the subject with continued nivolumab or ipilimumab dosing.

6.7 Treatment of Nivolumab or Ipilimumab Related Infusion Reactions

Since nivolumab and ipilimumab contain only human immunoglobulin protein sequences, it is unlikely to be immunogenic and induce infusion or hypersensitivity reactions. However, if such a reaction were to occur, it might manifest with fever, chills, rigors, headache, rash, pruritis, arthralgias, hypo- or hypertension, bronchospasm, or other symptoms.

All Grade 3 or 4 infusion reactions should be reported as an SAE if criteria are met. Infusion reactions should be graded according to NCI CTCAE v 4.0 guidelines.

Treatment recommendations are provided below and may be modified based on local treatment standards and guidelines as appropriate:

For Grade 1 symptoms: (Mild reaction; infusion interruption not indicated; intervention not indicated)

Remain at bedside and monitor subject until recovery from symptoms. The following prophylactic premedications are recommended for future infusions: diphenhydramine 50 mg (or equivalent) and/or paracetamol 325 to 1000 mg (acetaminophen) at least 30 minutes before additional nivolumab administrations.

For Grade 2 symptoms: (Moderate reaction requires therapy or infusion interruption but responds promptly to symptomatic treatment [eg, antihistamines, non-steroidal anti-inflammatory drugs, narcotics, corticosteroids, bronchodilators, IV fluids]; prophylactic medications indicated for 24 hours).

Stop the nivolumab or ipilimumab infusion, begin an IV infusion of normal saline, and treat the subject with diphenhydramine 50 mg IV (or equivalent) and/or paracetamol 325 to 1000 mg (acetaminophen); remain at bedside and monitor subject until resolution of symptoms. Corticosteroid or bronchodilator therapy may also be administered as appropriate. If the infusion is interrupted, then restart the infusion at 50% of the original infusion rate when symptoms resolve; if no further complications ensue after 30 minutes, the rate may be increased to 100% of the original infusion rate. Monitor subject closely. If symptoms recur then no further nivolumab or ipilimumab will be administered at that visit. Administer diphenhydramine 50 mg IV, and remain at bedside and monitor the subject until resolution of symptoms. The amount of study drug infused must be recorded on the electronic case report form (eCRF). The following prophylactic premedications are recommended for future infusions: diphenhydramine 50 mg (or equivalent) and/or paracetamol 325 to 1000 mg (acetaminophen) should be administered at least 30 minutes before additional nivolumab or ipilimumab administrations. If necessary, corticosteroids (recommended dose: up to 25 mg of IV hydrocortisone or equivalent) may be used.

For Grade 3 or Grade 4 symptoms: (Severe reaction, Grade 3: prolonged [ie, not rapidly responsive to symptomatic medication and/or brief interruption of infusion]; recurrence of symptoms following initial improvement; hospitalization indicated for other clinical sequelae [eg, renal impairment, pulmonary infiltrates]). Grade 4: (life threatening; pressor or ventilatory support indicated).

Immediately discontinue infusion of nivolumab or ipilimumab. Begin an IV infusion of normal saline, and treat the subject as follows. Recommend bronchodilators, epinephrine 0.2 to 1 mg of a 1:1,000 solution for subcutaneous administration or 0.1 to 0.25 mg of a 1:10,000 solution injected slowly for IV administration, and/or diphenhydramine 50 mg IV with methylprednisolone 100 mg IV (or equivalent), as needed. Subject should be monitored until the investigator is comfortable that the symptoms will not recur. Nivolumab or ipilimumab will be permanently discontinued. Investigators should follow their institutional guidelines for the treatment of anaphylaxis. Remain at bedside and monitor subject until recovery from symptoms. In the case of late-occurring hypersensitivity symptoms (eg, appearance of a localized or generalized pruritis within 1 week after treatment), symptomatic treatment may be given (eg, oral antihistamine, or corticosteroids).

6.8 Preparation and labeling of Investigational Medicinal Product

Preparation and labeling of the investigational medicinal products will be performed by the pharmacy of the NKI according to in-house SOP.

6.9 Drug accountability

Drug accountability will be performed by the pharmacy of the NKI according to internal standards.

6.10 Use of co-intervention

6.10.1 Prohibited medication

1) Concurrent chemotherapy, hormonal therapy, immunotherapy regimens, standard or investigational.
2) Use of growth factors including, but not limited to, granulocyte colony stimulating factor (G-CSF), granulocyte macrophage colony stimulating factor (GM-CSF), or erythropoietin stimulating agents are not permitted, unless deemed necessary by investigator and discussed with medical monitor.
3) Use of systemic corticosteroids at > 10 mg daily prednisone equivalent, unless required for the treatment of infusion reactions, other adverse events, or for palliation as determined by the investigator.
4) Steroids must not be given as prophylactic anti-emetic therapy.
5) Use of herbal remedies is not permitted.

6.10.2 Permitted medication

The use of prescription and over-the-counter medications (except medications from categories outlined above) is permitted at the discretion of the Investigator and must be recorded on CRF.
Subjects are permitted the use of topical, ocular, intranasal, intra-articular, and inhalational corticosteroids (with minimal systemic absorption). Immunosuppressive doses (e.g., prednisone > 10 mg/day or equivalent) and/or physiologic replacement doses of systemic corticosteroids (e.g., prednisone <10 mg/day) are permitted in the context of treating adverse events. A brief course of corticosteroids for prophylaxis

(e.g., contrast dye allergy) or for treatment of non-autoimmune conditions (e.g., delayed-type hypersensitivity reaction caused by a contact allergen) is permitted. Prophylactic anti-emetics, with the exception of steroids, may be administered at the discretion of the treating physician before any doses of study drug.
Use of the seasonal killed influenza vaccine during therapy is permitted without restriction. However, influenza vaccines containing live attenuated virus (Flumist ®) or other clinically indicated vaccinations for infectious diseases (killed or attenuated, e.g., Pneumovax ®, varicella, MMR, etc) may be permitted, but must be discussed with the PI and may require a study drug washout period prior to and after administration of the vaccine.

6.10.3 Contraception

A Woman of Childbearing Potential (WOCBP) is defined as any female who has experienced menarche and who has not undergone surgical sterilization (hysterectomy or bilateral oophorectomy) and is not postmenopausal. Menopause is defined as 12 months of amenorrhea in a woman over age 45 years in the absence of other biological or physiological causes. In addition, females under the age of 55 must have a serum follicle stimulating hormone, (FSH) level > 40IU/L to confirm menopause.
Females treated with hormone replacement therapy, (HRT) are likely to have artificially suppressed FSH levels and may require a washout period in order to obtain a physiologic FSH level. The duration of the washout period is a function of the type of HRT used. The duration of the washout period below are suggested guidelines and the investigators should use their judgment in checking serum FSH levels. If the serum FSH level is > 40 mIU/ml at any time during the washout period, the woman can be considered postmenopausal:

- 1 week minimum for vaginal hormonal products, (rings, creams, gels)
- 4 week minimum for transdermal products
- 8 week minimum for oral products

Other parenteral products may require as long as 6 months.

7. TUMOR RESPONSE ANALYSIS / TRANSLATIONAL RESEARCH

Tumor response analyses will be performed using various modalities: imaging, tissue samples and peripheral blood samples.

7.1 Imaging

In addition to standard baseline work-up of patients, one extra MRI and one extra FDG-PET scan are made after neoadjuvant immunotherapy but before surgery.

7.1.1 Multi-parametric MRI

In addition to the standard-of-care baseline MRI, one extra MRI will be made after neoadjuvant immunotherapy but before surgery. It will be used for secondary endpoint measurements. MRI uses intravenous administered Gadolinium contrast to visualize and quantify perfusion parameters, and diffusion of water molecules to reflect tissue cellularity. Tissue perfusion and cellularity are related to tumor hypoxia and necrosis, and also to local availability of systemic treatments. Many publications have shown prognostic and predictive value of MRI-derived parameters in head-neck cancer. First results in literature suggest potential correlations with response to immunotherapy [53].

7.1.2 FDG-PET

For study purposes 1 extra FDG PET is made after immunotherapy but before surgery. Tumor response to neoadjuvant immunotherapy will be assessed in terms of pathological response in view of RECIST 1.1 criteria (MRI and FDG PET). Patients will undergo one FDG PET routinely before treatment.

7.2 Tissue samples and assays

Tumor biopsies will be taken before and after nivolumab w/wo ipilimumab neoadjuvant to standard of care (SOC, surgery w/wo adjuvant (Cisplatin)RT).

The first tumor biopsies (before neoadjuvant treatment) are taken during routine diagnostic investigation under general anesthesia. During EUA, the tumor will be tattoo marked with a dotted line, to ensure optimal resection without compromising surgical margins in case of tumor response to immunotherapy. The second biopsies

(after neoadjuvant treatment) are taken from the therapeutic surgical tumor excision specimen.

In total, patients will undergo 2 extra tumor biopsies (8 mm) for research purposes twice: 1. during routine diagnostic EUA and 2. during curative surgical treatment. During EUA, also1 small biopsy (1-2 mm) is taken from benign tissue adjacent to the tumor.

See **figure 3** for a schematic overview of tumor and normal tissue biopsies needed for the various assays according to priority, in case not enough tissue can be harvested to perform all assays we wish for.

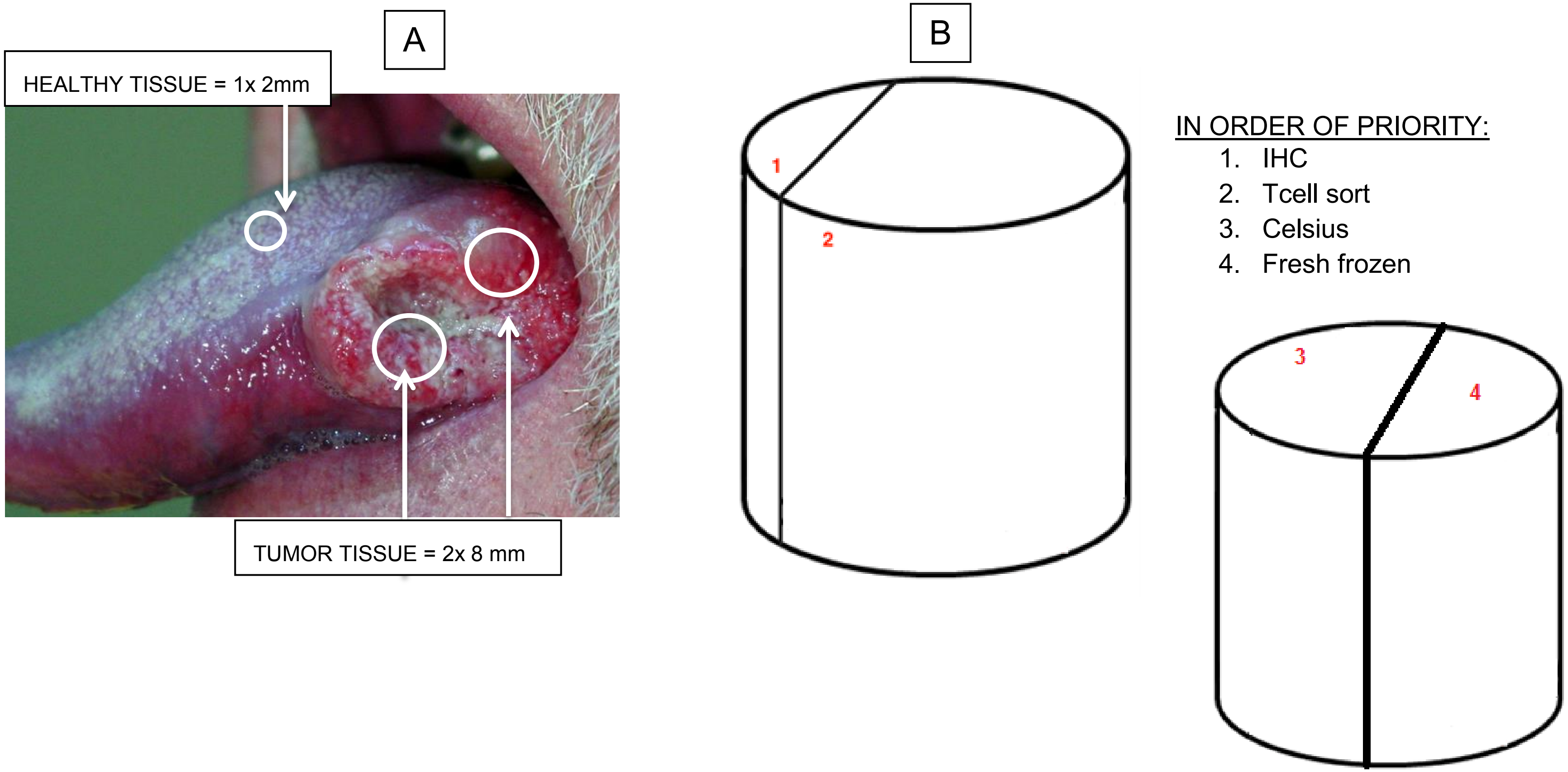

Figure 3A: Before and after neoadjuvant immunotherapy, two 8-mm biopsies will be taken from the tumor. In addition, one 1-2 mm biopsy will be taken from adjacent normal mucosa (to set up the Luminex assay), only once, before neoadjuvant immunotherapy. Figure 3B: Schematic representation of the two tumor biopsies, divided in proportions needed for the various assays, according to priority. First priority is IHC for immune infiltrates and HIF1α; second priority is digest for T cell sort; third priority (second tumor biopsy) material storage and shipping to Celsius, and the fourth priority is storing fresh-frozen tumor material for analyses including Luminex / bulk RNAseq / determining of DCs. Of note: the tumor pathological response to neoadjuvant immunotherapy will be determined by our HN pathologist comparing the fully surgically excised tumor in comparison to the biopsy that was taken for diagnosis during routine work-up of the patient.

When the biopsies are taken during routine EUA (see figure 1 of this trial), it is our experience that patients may experience slight discomfort. There is a small chance of <1% of complications (i.e. infection or bleeding). Biopsies will be taken from well and safe accessible primary tumors by an experienced head and neck surgeon or fellow. Standard procedures will be followed in case of complications, to assure the safety of the patient. When the biopsies are taken during curative surgery, patient will obviously not endure any extra side-effects post-surgery as the tumor specimen will be fully resected.

Various fundamental assays are applied to the tissue samples to study T cell function (7.2.1), tumor immune infiltrates (7.2.2), cytokine production (7.2.3), hypoxia (7.2.4), microbiomics and metabolomics (7.2.6), ctDNA (7.4.4), single cell omics (7.4.5) and dendritic cells in tumor-draining lymph nodes (7.4.6).

In addition, the tumor pathological response to neoadjuvant immunotherapy will be assessed by our HN pathologist the tumor pathological response to neoadjuvant immunotherapy will be determined by our HN pathologist comparing the fully surgically excised tumor in comparison to the biopsy that was taken for diagnosis during routine work-up of the patient (7.3.5)

7.2.1 T cell capacity

T cell capacity (function / transcriptional state) in hypoxic and normoxic tumor tissue will be assessed using immunological assays. After the tumor is digested to obtain a single cell suspension, FAC-sorting of T-cells will be performed and transcriptome analysis of the tumor-infiltrating T cells will be performed.
With the sort, the surface expression of activation and exhaustion markers on the T cells are analyzed to overlay phenotypical characteristics with gene expression profiles. Single cell RNA sequencing will be performed in collaboration with a third party outside the NKI-AVL, the Weizmann Institute of Science, Rehovot.

7.2.2 Tumor immune infiltrates

Abundance of T cells and other immune cells will be investigated using the following stains:

- T cells (CD3/CD8/FOXp3)
- M1 / M2 macrophages: CD163, CD68 and CD206.
- Myeloid cells: CD68+, CD163+. Myeloid maturation/activation: HLA-DR+. For completion of the myeloid panel, CK and CD66b will be included.
- MDSCs: HLA-DR- CD163+, and HLA-DR- CD66b+
- Molecular targets in immunotherapy will be assessed by:
    - HLA IHC class I: Beta-2-Microglobulin/HLA_A/HLA_BC
    - HLA IHC class II: HLA_DRA/HLA_DR_DP_DQ
    - checkpoint molecules (PD-1 / PD-L1).

Gene expression analysis of bulk tumor tissue will be done to amongst others to look for metabolic and immune signatures within the biopsies taken from hypoxic and normoxic areas.

7.2.3 Tumor cytokine production

Luminex assay will be used to assess the cytokines of the immune infiltrates in both tumor and blood. The Luminex assay will be performed in the Luminex core facility of the University Medical Center of Utrecht.

7.2.4 Monitoring tumor tissue hypoxia.

Gene expression analysis of bulk tumor tissue will be done amongst others to look for validated hypoxia signatures within the biopsies taken from hypoxic and normoxic areas. Recently, a 15-gene hypoxia classifier was validated in 323 patients with HNSCC randomized for hypoxic modification or placebo in combination with radiotherapy. Tumors categorized as hypoxic on the basis of the classifier were associated with a significantly poorer clinical outcome than nonhypoxic tumors [13]. In addition, technical validation of the 15-gene hypoxia classifier demonstrated that it is suitable for implementation in prospective clinical trials as well [14].

7.2.5 Tumor pathological response to neoadjuvant immunotherapy.

The pathological response of the tumor to neoadjuvant immunotherapy will be scored by our head and neck pathologists.
It is defined as percentage residual tumor cells after neoadjuvant immunotherapy by comparing the tumor tissue biopsies before and after nivolumab w/wo ipilimumab, according to existing guidelines to assess pathological tumor response to neoadjuvant therapy [31].

Categories will be:

- Complete pathological response: or (i) no residual invasive carcinoma, or (ii) no residual invasive carcinoma, however still epithelial carcinoma in situ.
- Partial reponse: or (i) minimal residual invasive carcinoma (only some single or disjoined groups of tumor cells, or (ii) clear response to therapy, with 10-15% residual invasive carcinoma, or (iii) >50% invasive tumor present, however with characteristics of response (ao fibrosis).
- No response: No indication for tumor response to neoadjuvant immunotherapy.

In addition, the tumor immune infiltrate will be scored by a head and neck pathologist. Analogous to scoring in melanoma categories will be: absent, non-brisk and brisk. In case of “brisk” the pathologist will distinguish between central in the tumor, the invasive front of the tumor and the peripheral stroma.

7.2.6 Dysbiosis as a biomarker for response to immunotherapy

This translational research will be performed in collaboration with the Department of Medical Microbiology, Experimental Microbiology and Chemical Immunology from the Leiden University Medical Center (LUMC).

Swabs will be taken at the tumor site and at 4 surrounding tissue sites in the oral cavity and oropharynx (see figure below). In total, 20 swabs are taken: 5 sites times 2 swabs (one for microbiome, one for metabolome), before and after immunotherapy. Swabs are taken at routine investigation under general anesthesia before immunotherapy and during surgery after immunotherapy. Swabs will be frozen (-80°C) until further processing for microbiome and metabolite analysis.

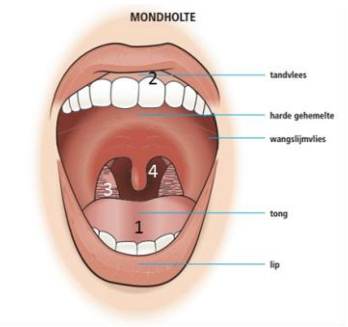

Figure 1: Swabs will be taken before and after immunotherapy at distinct habitats in the oral cavity: tooth surface/gingival crevice (1), tongue (2), tonsils (3) and oropharynx (4) with specific microbes, and at the primary tumor site.

Stool will be collected before and after immunotherapy. Patients will receive a stool collection kit with instructions for the collection of stool at home. Patients will be requested to hand in the stool sample in a feces container (15mL) at the days they visit the hospital for routine investigation or surgery on the same days mucosal swabs will be collected. Stool will be frozen (-80°C) until further processing for microbiome analysis.

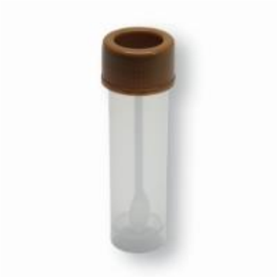

7.3 Tattoo tumor delineation

During EUA (before neoadjuvant treatment), the tumor will be tattoo marked with single dots, to ensure adequate resection of the original tumor tissue volume in case of clinically overt tumor response to neoadjuvant immunotherapy. Diabolo Genesis Ultimate Black ink [54] and a Kwadron round liner, 0,3mm long taper will be used [55], with a distance in between punctures of 0,5 - 1cm and a puncture depth of approximately 1,5mm. Punctures should be performed WITHOUT TWISTING of the needle. After consulting with specialists in the field [56], no significant complications are expected (e.g. pain, swelling, infection).

7.4 Sample assays: PBMCs, serum, microbiome, ctDNA and single cell-omics

7.4.1 PBMCs

PBMCs will be used to analyze the gene expression profiles of T cells in blood before and after neo-adjuvant immunotherapy, representing the gene expression changes in steady state circulating T cells. In addition, blood can be used for phenotypical analysis of T cells in blood before and after immunotherapy. For blood collection, 5x10mL natrium heparinized blood bottles are used.

7.4.2 Serum / Luminex

For longitudinal analysis over time, peripheral blood (serum) will be used. Serum can be extracted and stored in serum blood bottles. One bottle of 5mL should be sufficient for analysis. Serum tube should stand for 30-45 minutes (max 60 minutes) at room temperature. Next it should be centrifuged 10-15 minutes (depending on rotor 1000-2000 RCF). Serum aliquots must be processed as fast as possible (within 4 hours maximum) and stored at -80°C. 1 mL should be sufficient for analysis. It's imperative that the same blood drawing tube (brand and volume) is used at all times.

7.4.3 Microbiome and metabolome

To assess the oral-, oropharyngeal and fecal microbiome, which includes bacteria, archea, viruses and fungi. RNA and DNA will be isolated from oral swabs and feces

for sequencing of the metagenome. In addition, the oral and oropharynx metabolite profile will be determined by mass spectrometry.

Mucosal swab samples will be extracted with methanol and the methanol fraction will be lyophilized. The sample will then be prepared for metabolite analysis by mass spectrometry, and data will be analyzed by the information platform available at the LUMC (Department of Medical Microbiology and Chemical Immunology Research team: J.J.C. Neefjes, M.A. Giera, R.D. Zwittink, E.J. Kuijper, A.L. Ciurli, M. Slingerland).

7.4.4 ctDNA in liquid biopsies

Pre-and post-immunotherapy blood will be obtained for various clinical evaluations and study-related objectives. During the venous puncture, additional blood will be obtained and cryopreserved for this analysis. ctDNA will be isolated through the Quick-cfDNA serum and plasma kit (Zymo). The frequency of TP53 mutations in the blood will be determined using a novel technique (Cyclomics), based on Oxford Nanopore MinION sequencing of concatenated copies of a single DNA molecule. Levels of TP53 mutation in peripheral blood before and after immunotherapy will be compared and correlated to clinical outcome and other parameters such as the MRI and FDG-PET scans, to dissect whether it may act as a minimally invasive biomarker for immunotherapy response. Analyses of ctDNA will be performed at the UMCU Center for Molecular Medicine, under supervision of Wigard Kloosterman, Jeroen de Ridder and Myrthe Jager.

7.4.5 Single cell sequencing of matched patient samples

We will use single-cell sequencing technology to analyze our patient’s tumor samples at baseline, after immunotherapy and in the case of tumor recurrence. One half of the 8mm tumor core samples will be dedicated to this analysis.
For single-cell sequencing of patient tumor samples, a collaboration with a third party outside of the NKI-AVL Celsius Therapeutics Inc. (Cambridge, MA, USA) has been established. Celsius Therapeutics Inc. has developed a commercial single-cell sequencing pipeline and automated data analysis that we will use to process our patient’s tumor samples (half of an 8mm punch biopsy) obtained at baseline (pre-treatment), after neoadjuvant immunotherapy and in case of tumor recurrence. Samples will be stored and preserved in shipping kits provided by Celsius

Therapeutics Inc. (including tubes, stabilization buffer and shipping boxes with cooling system) shortly after collection. Shipping kits will be sent to the Celsius Therapeutics Inc. laboratory in the USA for analysis.

7.4.6 Dendritic cells in tumor-draining lymph nodes

The role of Type 1 conventional dendritic cells (cDC1) in tumor-draining lymph node (tdLN) is to cross-present tumor antigens and prime anti-tumor CTL response, and in the tumor microenvironment (TME) is to re-stimulate tumor infiltrating T cells and help to sustain the tumor-specific cytotoxic T lymphocyte (CTL) response in tumor site locally, which explains why the abundance of cDC1s in TME is positively correlates with the responsiveness to anti–PD-1 therapy and results in a better survival of cancer patients (Böttcher *et al.* The Role of Type 1 Conventional Dendritic Cells in Cancer Immunity. *Trends in Cancer* 2018). Our preliminary study indicates that the human cDC1 is the most capable DC subset in relaying the “help” signal from the $CD4^+$ T cell to generate a tumor-specific CTL response (Xiao *et al.* unpublished data). Therefore in addition of monitoring tumor infiltrating CD8 T cells, macrophages and MDSCs (as described in 7.2.2 of the IMCISION proposal), the presence of cDC1 and activated CD4 T cells and their phenotype in tdLN and TME also need to be measured. This information could be an indicator for identifying the responder patients.

We propose to obtain biopsies of tdLN, non tdLN (control samples) and tumor tissues (size = or 4mm). All biopsy samples will be cryopreserved according to the procedure of viable lymph node and tumor tissue preparation until final use.

8. STUDY METHODS

8.1 Study endpoints

Primary endpoints:

- Phase Ib: Primary endpoint is measured as the number of patients that will not endure a delay in surgery (surgery should be performed in week 5-6) due to neoadjuvant immunotherapy (nivolumab, ipilimumab) related toxicity (measured in terms of SAEs and CTCAE v4.0) OR the treatment of immunotherapy related toxicity (ie high dose corticosteroids)**.
  ** To meet this endpoint, all patients will be discussed in our immunotherapy team meeting (consisting of at least medical oncologist and head and neck surgeon) the week before surgery, to evaluate whether immunotherapy-related toxicity or treatment of immunotherapy-related toxicity will lead to delay in surgery or not.
  ** Delay in surgery due to logistical problems (i.e. no IC bed after surgery) or other co-morbidity (i.e. bacterial pneumonia) will not be considered dose-limiting toxicity.
- Phase II: Tumor response to neoadjuvant IT in terms of tumor tissue pathological response at time of surgery compared to RECIST 1.1 (FDG-PET and perfusion and diffusion weighted MRI).

Secondary endpoints:

- We will monitor immune cell subsets and cytokines in the peripheral blood and tumor compartment.
- Rate and type of late AEs (NCI CTCAE v 4.0) up to 2 years FU after SOC (see Figure 1).
- Relapse free survival (RECIST 1.1) and overall survival at 2 years follow-up.
- The rate of tumor hypoxia before and after immunotherapy.
- The oral and gut microbiome (comprising bacteria, archea, viruses and fungi) and the oral metabolome profile in patients with advanced HNSCC before and after anti-cancer immune therapy.
- The applicability of ctDNA in 'liquid biopsies' as a biomarker for immunotherapy response.

- The transcriptome[5] of all, single cells in patient tumor material before and after two cycles of neoadjuvant immunotherapy and in the event of tumor recurrence.
- The abundance of cDC1 cells in tumor-draining lymph nodes, non-tumor draining lymph nodes and primary tumor tissue.

For details concerning the imaging and assays needed for reaching these endpoint, see chapter 7.

8.2 Screening phase

Following signing of the informed consent form for screening and enrolment into the study, the remainder of screening procedures and tests will be completed.

- Complete physical examination including height, weight, performance status, and vital signs, noting in detail the exact size and location of any lesions that exist.
- Patients may enter the study with a pathologic diagnosis of HNSCC from any institution. Diagnosis must then be confirmed by revision or new biopsies taken at the NKI, Department of Pathology. HPV status of the tumor will be determined by PCR.
- Hematology (Absolute neutrophil count (ANC), Hb, platelet count), Chemistry (ALAT/ ASAT, serum creatinine, total bilirubin))
- Routine baseline PET/CT and MRI head and neck to evaluate the status of disease.
- ECG HIV antibody titer and HbsAg determination, Anti-HCV, anti-CMV antibody titer, and HSV serology, EBV panel and lues panel.
- beta-HCG pregnancy test on all women of child-bearing potential
- PBMC’s and serum will be taken before start of treatment
- Routine MRI and PET scanning.
- Signed ICF

8.3 Off study criteria

In case of screening failure, the study subject will be excluded for investigational treatment, by which the original treatment slot will be released.

8.4 Randomization, blinding and treatment allocation

There is no randomization and no blinding.

Patients fulfilling the eligibility criteria will be enrolled in the study. Patients will receive either nivolumab as a single agent (Cohort 1) or a combination of nivolumab+ipilimumab (Cohort 2) neoadjuvant to surgery. From the phase Ib we will determine which neoadjuvant immunotherapy regimen (from cohort I or 2) will be applied in the phase II extension cohort 3.

8.5 During treatment

Figure 1 summarizes timelines, study design, diagnosis, screening, treatment, imaging and study assays.

Figure 1: Study design and timeline of diagnosis, screening, treatment, study assays and follow-up

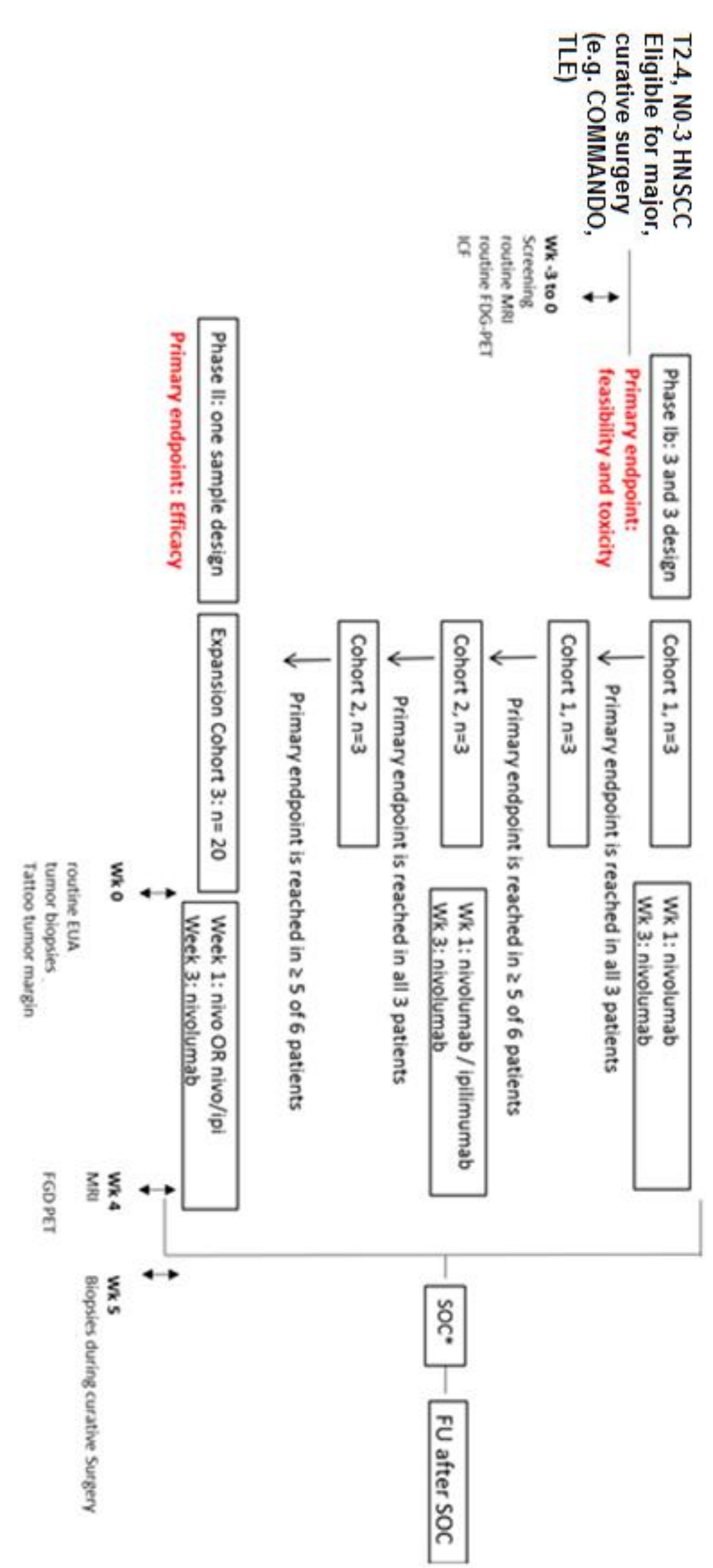

SOC*=Standard of care: surgery w/wo adjuvant (C)RT, surgery is planned in week 5, adjuvant (C)RT will be planned 4-6 weeks after surgery.

Research treatment and assessments are shown in table 1 (alterations to the protocol added in this version (7.1) are marked yellow)

Table 1. Detailed routine (white) and research (blue) treatment and assessments

	routine work-up and screening	week -1	week 1-3	week 4-5	week 5	2 year FU after SOC[1,2]	
**Treatment**			Nivo or Nivo-Ipi		SOC: surgery w/wo (C)RT		
Medical history	x						
Physical examination[3]	x		x		x	4x	3x
ECG	x						
Examination under anesthesia (EUA)		x					
Signing ICF	x						
Blood collection							
a. Hematology[4]	x		x		x	4x	4x[11]
b. Chemistry[5]	x		x		x	4x	4x[11]
c. Serology[6]	x						
d. beta-HCG pregnancy test[7]	x		x				
e. Coagulation: PT/INR and aPTT	x						
f. PBMC[8]		x	x				4x[11]
g. Luminex[9]		x	x				4x[11]
h. ctDNA blood sample[13]		x			x		
Imaging							
a. MRI	x			x			
b. FDG PET	x			x			
Biopsies							
a. Tumor (2x8mm)[10]	x	x			during curative surgery	x[12]	x[12]
b. Benign (1x2mm)		x					
c. Tumorous lymph node (half)14					during curative surgery		
d. Benign lymph node (half)					during curative surgery		
Microbiome and metabolome							
a. Oral swabs		x			x		
b. Faeces		x		x			
Adverse events			x		x		7x

1: Standard Of Care (SOC) is surgery w/wo (C)RT. Adjuvant (C)RT will be planned 4-6 weeks after surgery. FU starts after SOC.
2: For study purposes FU of 2 years after SOC is needed. No extra visits are needed for research.
Routine FU: Year 1: every 3 months. Year 2: every 4 months. In total 7 visits in 2 years.
3: ECOG performance status, weight, temperature, pulse, blood pressure
4: Hb, ANC, platelet count incl. differentiation, Hct, 3 mL total
5: LDH, phosphorus, sodium, potassium, magnesium, chloride, calcium, creatinine, albumin, total protein, SGOT (AST), SGPT (ALT), bilirubin (ind + dir), GGT, alkaline phosphatase, glucose, lipase, TSH, fT4, ACTH, cortisol, LH, FSH, testosterone/oestradiol, S100, CRP, ESR. 5 mL total.
6: HIV, HbsAG, HCV, anti-CMV, HSV, EBV and lues; 10 mL total.
7: Only WOCBP, goes with chemistry, no extra mL blood needed.
8: At screening 100mL, all other samples 50mL
9: 5mL (serum bottle)
10: 1x 8mm biopsy will be stored here, the other will be halved with half sent to the USA for analysis by Celsius and other to be stored here
11: One blood draw will be taken directly 1 week after sugery
12: In routine care, 1 biopsy is taken for diagnosis in case of disease progression. Patients will be asked for 1 extra biopsy of 2mm for research (not obligatory).
13: ctDNA blood sample 10 mL, before immune therapy and after immune therapy, 20 mL in total per patient
14: Patients will be asked for half of a tumorous lymph node and half of a benign node. This is not obligatory. Nodes will be obtained via standard-of-care neck dissection.

Notes:
I: at screening, 13 mL extra blood is needed (coagulation and serology. For pregnancy test no extra mL blood is needed).
II: during the study, the first time 105 mL extra blood is needed (PBMC 100 mL, Luminex 5 mL), for the second infusion 63mL.
Thereafter, at 4 time points extra blood is needed in more than 2 years time: 63 mL (PBMC 50, Luminex 5, Chemistry 5, Hematology 3)
III: In total, for study purposes in more than 2 years time 453 mL extra blood is needed.
IV: In total patients will undergo 7 extra blood draws for study purposes: week -1, twice before immunotherapy infusion, and 4 times during 2 years FU.
These blood draws are taken during (study) treatnment or routine FU. Patients do not need to come extra for this.

8.6 Post treatment evaluation

Primary endpoints will be reached 12 weeks after SOC. For secondary endpoints a 2-year FU is needed. After that, patients will go into routine follow up, to a total of 5 years (3 more years).

In case of tumor relapse biopsies, PBMC and serum collection will be performed. In routine care, in case of disease progression one biopsy for diagnosis is taken. Also, the patient will be asked to allow for harvesting one extra biopsy for research

8.7 Withdrawal of individual subjects

Subjects can leave the study at any time for any reason if they wish to do so without any consequences. The investigator can decide to withdraw a subject from the study for urgent medical reasons. If after having signed informed consent but before initiation of the treatment, patients do not fulfill the eligibility criteria anymore, patients will be withdrawn from the study.

8.8 Replacement of individual subjects after withdrawal

Patients that are withdrawn prior to initiation of the treatment (defined as start neoadjuvant immunotherapy) and patients that do not fulfill the eligibility criteria will be replaced.

8.9 Follow-up of subjects withdrawn from treatment

Patients that are withdrawn from study before administration of the first dose of immunotherapy will receive standard treatment of care and follow up. If distant metastases are identified during imaging after drug administration though prior to surgery, patients will be treated (palliatively) off-study conform standard of care, defined multidisciplinary. These, and/or patients withdrawn from study due to treatment toxicity will continue study follow up at least until 12 weeks after SOC or until treatment related toxicity has resolved to grade 2 or less (CTCAE 4.0). Thereafter, patients will receive standard follow-up.

8.10 Premature termination of the study

In case of unexpected toxicity or feasibility the sponsor will discuss premature termination of the study with the ethical committee and with BMS, see paragraph 10.1.

9. SAFETY REPORTING

9.1 Section 10 WMO event

In accordance to section 10, subsection 1, of the WMO, the investigator will inform the subjects and the reviewing accredited METC if anything occurs, on the basis of which it appears that the disadvantages of participation may be significantly greater than was foreseen in the research proposal. The study will be suspended pending further review by the accredited METC, except insofar as suspension would jeopardise the subjects' health. The investigator will take care that all subjects are kept informed.

9.2 AEs, SAEs and SUSARs

9.2.1 Adverse events (AEs)

An Adverse Event (AE) is defined as any new untoward medical occurrence or worsening of a preexisting medical condition in a clinical investigation subject administered an investigational (medicinal) product and that does not necessarily have a causal relationship with this treatment. An AE can therefore be any unfavorable and unintended sign (such as an abnormal laboratory finding), symptom, or disease temporally associated with the use of investigational product, whether or not considered related to the investigational product.

The causal relationship to study drug is determined by a physician and should be used to assess all adverse events (AE).
Items to be considered when assessing the relationship of an adverse event to the study treatment are:

- Temporal relationship of the onset of the event to the initiation of the study treatment;
- The course of the event, considering especially the effect of discontinuation of study treatment or reintroduction of study treatment, as applicable;
- Whether the event is known to be associated with the study treatment, or with other similar treatments;
- The presence of risk factors in the study subject known to increase the occurrence of the event;

- The presence of non-study treatment related factors, which are known to be associated with the occurrence of the event.

The relationship with an adverse event to study treatment will be reported in the patient's file and defined as: 'unrelated', 'unlikely', 'possible', 'probable' or 'definite'.

Unrelated: the event is clearly related to other factors such as the patient's clinical state, other therapeutic interventions or concomitant drugs administered to the patient,
Unlikely: the toxicity is doubtfully related to the investigational agent. The event was most likely related to other factors, such as the patient's clinical state, other therapeutic interventions, or concomitant drugs,
Possible: the event follows a reasonable temporal sequence from the time of drug administration, but could have been produced by other factors such as the patient's clinical state, other therapeutic interventions or concomitant drugs,
Probable: the event follows a reasonable temporal sequence from the time of drug administration, and follows a known response pattern to the study drug. The toxicity cannot be reasonably explained by other factors such as the patient's clinical state, therapeutic interventions or concomitant drugs,
Definite: the event follows a reasonable temporal sequence from the time of drug administration, and follows a known response pattern to the study drug.

Adverse events can be spontaneously reported or elicited during open-ended questioning, examination, or evaluation of a subject. (In order to prevent reporting bias, subjects should not be questioned regarding the specific occurrence of one or more AEs.)

<u>Non-serious Adverse Event</u>

- Non-serious Adverse Events are to be provided to BMS in aggregate via interim or final study reports as specified in the agreement or, if a regulatory requirement [e.g. IND US trial] as part of an annual reporting requirement.
- Non-serious AE information should also be collected from the start of a placebo lead-in period or other observational period intended to establish a baseline status for the subjects.

A non-serious adverse event is an AE not classified as serious.

Non-serious Adverse Event Collection and Reporting

The collection of non-serious AE information should begin at initiation of study drug. All non-serious adverse events (not only those deemed to be treatment-related) should be collected continuously during the treatment period and for a minimum of 100 days following the last dose of study treatment.

Non-serious AEs should be followed to resolution or stabilization, or reported as SAEs if they become serious. Follow-up is also required for non-serious AEs that cause interruption or discontinuation of study drug and for those present at the end of study treatment as appropriate.

9.2.2 Serious adverse events (SAEs)

A Serious Adverse Event (SAE) is any untoward medical occurrence that at any dose:

- Results in death
- Is life-threatening (defined as an event in which the subject was at risk of death at the time of the event; it does not refer to an event which hypothetically might have caused death if it were more severe)
- Requires inpatient hospitalization or causes prolongation of existing hospitalization (see note below)
- Results in persistent or significant disability/incapacity
- Is a congenital anomaly/birth defect
- Is an important medical event (defined as a medical event(s) that may not be immediately life-threatening or result in death or hospitalization but, based upon appropriate medical and scientific judgment, may jeopardize the subject or may require intervention [eg, medical, surgical] to prevent one of the other serious outcomes listed in the definition above.) Examples of such events include, but are not limited to, intensive treatment in an emergency room or at home for allergic bronchospasm; blood dyscrasias or convulsions that do not result in hospitalization.)

- Potential drug induced liver injury[#], renal failure, or pneumonitis are also considered an important medical event.

[#] Potential Drug Induced Liver Injury (DILI): wherever possible, timely confirmation of initial liver-related laboratory abnormalities should occur prior to the reporting of a potential DILI event. All occurrences of potential DILIs, meeting the defined criteria, must be reported as SAEs Potential drug induced liver injury is defined as:

1) ALT or AST elevation > 3 times upper limit of normal (ULN) AND
2) Total bilirubin > 2 times ULN, without initial findings of cholestasis (elevated serum alkaline phosphatase) AND
3) No other immediately apparent possible causes of AST/ALT elevation and hyperbilirubinemia, including, but not limited to, viral hepatitis, pre-existing chronic or acute liver disease, or the administration of other drug(s) known to be hepatotoxic.

- Suspected transmission of an infectious agent (eg, pathogenic or nonpathogenic) via the study drug is an SAE.
- Although pregnancy, overdose, and cancer are not always serious by regulatory definition, these events must be handled as SAEs.

9.2.3 Reporting of SAEs

All Serious Adverse Events (SAE) occurring from registration until 100 days after the last protocol treatment/administration should be documented and reported (the study specific SAE form should be used) immediately to the NKI-AVL Safety Desk by fax: 0031 (0)20-5122679 between 09.00 and 17.00 hours Monday to Friday. If a fax is not possible due to technical problems, the Data Centre should be contacted by telephone: 0031 (0)20-5122668) between 09.00 and 17.00 hours Monday to Friday.

The Safety Officer will notify the study coordinator immediately of any serious adverse event (as defined above) experienced by a patient. The study coordinator will evaluate the SAE and will decide whether the event reported could be related to the protocol treatment and whether it is, both, unexpected and serious (SUSAR, see 9.2.4.).

The NKI-AVL Safety Desk will report the SAE's by fax to the subsidizing party BMS Company Global Pharmacovigilance & Epidemiology, Fax Number: 609-818-3804 Email: worldwide.safety@bms.com. SAEs will be followed up by the safety department of the AVL. Updates on the SAE will be sent to BMS similarly.
In addition, adverse events will be reported by the NKI-AVL Safety Desk to the METC and the CCMO. All SAE's will be reported once yearly, as described in the section 8.3. SAE's will not be reported through the web portal ToetsingOnline to the METC.

Serious adverse events occurring more than 100 days after the last study medication will NOT be reported unless the investigator feels that the study drug or a protocol procedure may have caused the event.

Any adverse event leading to hospitalization or prolongation of hospitalization will be considered as 'serious', UNLESS at least one of the following exceptions are met:

- The admission is pre-planned (e.g. elective or scheduled surgery, documented in the patient's file);
- Hospitalization for technical (e.g. study drug administration), practical or social reasons, in the absence of an adverse event.

Common toxicities observed for progressive disease and events secondary to progressive disease are generally excluded from reporting. However, in cases where the specificity or severity of an event is not consistent with the risk information, the event should be reported.

All SAE reports will be filed in the Investigator Study File.
In case the SAE is unexpected a SUSAR will be reported (see section 12.2.4).

9.2.4 Suspected unexpected serious adverse reactions (SUSARs)

Adverse reactions are all untoward and unintended responses to an investigational product related to any dose administered.

Unexpected adverse reactions are SUSARs if the following three conditions are met:

1. The event must be serious (see chapter 9.2.2.);

2. There must be a certain degree of probability that the event is a harmful and an undesirable reaction to the medicinal product under investigation, regardless of the administered dose;
3. The adverse reaction must be unexpected, that is to say, the nature and severity of the adverse reaction are not in agreement with the product information as recorded in:
   - Summary of Product Characteristics (SPC) for an authorised medicinal product;
   - Investigator's Brochure for an unauthorised medicinal product.

The sponsor will report expedited the following SUSARs through the web portal *ToetsingOnline* to the METC:
- SUSARs that have arisen in the clinical trial that was assessed by the METC;
- SUSARs that have arisen in other clinical trials of the same sponsor and with the same medicinal product, and that could have consequences for the safety of the subjects involved in the clinical trial that was assessed by the METC.

The remaining SUSARs are recorded in an overview list (line-listing) that will be submitted once every half year to the METC. This line-listing provides an overview of all SUSARs from the study medicine, accompanied by a brief report highlighting the main points of concern.
The expedited reporting of SUSARs through the web portal ToetsingOnline is sufficient as notification to the competent authority.
The sponsor will report expedited all SUSARs to the competent authorities in other Member States, according to the requirements of the Member States.

The expedited reporting will occur not later than 15 days after the sponsor has first knowledge of the adverse reactions. For fatal or life threatening cases the term will be maximal 7 days for a preliminary report with another 8 days for completion of the report.

9.3 Annual safety report

In addition to the expedited reporting of SUSARs, the sponsor will submit, once a year throughout the clinical trial, a safety report to the accredited METC, competent authority, and competent authorities of the concerned Member States.
This safety report consists of:

- A list of all suspected (unexpected or expected) serious adverse reactions, along with an aggregated summary table of all reported serious adverse reactions, ordered by organ system, per study;
- A report concerning the safety of the subjects, consisting of a complete safety analysis and an evaluation of the balance between the efficacy and the harmfulness of the medicine under investigation.

9.4 Follow-up of adverse events

All AEs will be followed until they have abated, or until a stable situation has been reached. Depending on the event, follow up may require additional tests or medical procedures as indicated, and/or referral to the general physician or a medical specialist.
SAEs will to be reported till 100 days after discontinuation of the study drug.

9.5 Data Safety Monitoring Board (DSMB) / Safety Committee

No DSMB will be set-up.

10.STATISTICAL ANALYSIS

10.1 Sample size calculation

Phase Ib: 3 +3 design
When primary endpoint is reached in cohort 1 (**interim analysis** after 3 patients), we will continue to cohort 2.
When primary endpoint is reached in cohort 2 (**interim analysis** after 3 patients), we will expand to cohort 3.
When primary endpoint is not reached in cohort 2, we will expand cohort 1 to cohort 3.

Note: we need to see endpoints reached in all 6 patients of cohort 1 and 2, before we will continue to the next cohort.

Phase II: Single arm design
The extension cohort (cohort 3) will exist of at least 20 patients.

An overall < 10% incidence of pathological tissue response is considered not clinically relevant for patients enduring either neoadjuvant immunotherapy regimen.

With an expansion cohort of at least 20 patients, the total number of patients receiving a specific immunotherapy regimen will be ≥ at least 26.

A total of ≥ 26 patients will allow for 90% power and 90% confidence that in the population at large the incidence of pathological response is indeed ≥ 10%.

This calculation is based on the expected pathological response of HNC to nivolumab (approximately 30%, as tissue response is probably 10% higher than clinical response, C. Blank). In case of nivo/ipi combination , we expect that this percentage is higher.

We will perform an **interim analysis** after the first 10 patients of cohort 3 to reveal potential unexpected toxicity. Unacceptable toxicity is defined as >1 from 10 patients

that will experience a delay in surgery (surgery should be performed in week 5-6) due to neoadjuvant immunotherapy (nivolumab, ipilimumab) related toxicity (measured in terms of SAEs and CTCAE v4.0) OR the treatment of immunotherapy related toxicity (ie high dose corticosteroids). If this happens in cohort 1, the study will be closed until we have been able to formulate a new neoadjuvant treatment regimen / design for this arm, approved by the METC.

10.2 Safety analysis

Safety will be evaluated through the analysis of adverse events (AE), laboratory tests, physical examination, vital signs and performance status. All patients who received at least one administration of immunotherapy will be included in safety analysis. Adverse events will be tabulated by system organ class and by severity according to NCI CTCAE criteria, version 4.0.

10.3 Immune- and pathologic response analysis

See paragraph 7.

10.4 Clinical response evaluation

After collection of all the histological and immunological samples, observatory statistics will be used.

10.5 Interim analysis

Phase Ib:

Interim analysis will be performed after every 3 patients treated in cohort 1 and 2.

We need to see endpoints reached in all 6 patients of cohort 1 and 2, before we will continue to the next cohort.

Primary endpoint is measured as the number of patients that will not endure a delay in surgery (surgery should be performed in week 5-6) due to neoadjuvant immunotherapy (nivolumab, ipilimumab) related toxicity (measured in terms of SAEs and CTCAE v4.0) OR the treatment of immunotherapy related toxicity (ie high dose corticosteroids)**.

** To meet this endpoint, all patients will be discussed in our immunotherapy team meeting (consisting of at least medical oncologist and head and neck surgeon) the week before surgery, to evaluate whether immunotherapy-related toxicity or treatment of immunotherapy-related toxicity will lead to delay in surgery or not.
** Delay in surgery due to logistical problems (i.e. no IC bed after surgery) or other co-morbidity (i.e. bacterial pneumonia) will not be considered dose-limiting toxicity.

<u>Phase II:</u>
We will perform an interim analysis after the first 10 patients of cohort 3 to reveal potential unexpected toxicity.

Unacceptable toxicity is defined as >1 from 10 patients of cohort 3 that will experience a delay in surgery (surgery should be performed in week 5-6) due to neoadjuvant immunotherapy (nivolumab, ipilimumab) related toxicity (measured in terms of SAEs and CTCAE v4.0) OR the treatment of immunotherapy related toxicity (ie high dose corticosteroids).

As we need all patients from cohort 1 or 2 and 3 (at least 26) to determine efficacy , we will not perform an interim analysis for efficacy.
Efficacy is defined as: Tumor response to neoadjuvant IT in terms of tumor tissue pathological response at time of surgery and RECIST 1.1 (FDG-PET and perfusion and diffusion weighted MRI).

11.ETHICAL CONSIDERATIONS

11.1 Regulation statement

This study will be conducted according to the principles of the Declaration of Helsinki, (Declaration of Helsinki, 59th WMA General Assembly, Seoul, October 2008) and in accordance with the Medical Research Involving Human Subjects Act (WMO). The protocol has been written, and the study will be conducted according to the ICH Harmonized Tripartite Guideline for Good Clinical Practice [57].
The protocol must be approved by the CCMO and competent authority (CA).

11.2 Recruitment and consent

11.2.1 Informed consent (IC)

All patients will be informed of the aims of the study, the possible adverse events, the procedures and possible hazards to which he/she will be exposed, and the mechanism of treatment allocation. They will be informed as to the strict confidentiality of their patient data, but that their medical records may be reviewed for trial purposes by authorized individuals other than their treating physician.
It will be emphasized that the participation is voluntary and that the patient is allowed to refuse further participation in the protocol whenever he/she wants. This will not prejudice the patient’s subsequent care. Documented informed consent will be obtained for all patients included in the study before they are registered in the study. This will be done in accordance with the national and local regulatory requirements. The IC procedure will conform to the ICH guidelines on Good Clinical Practice. This implies that “the written IC form will be signed and personally dated by the patient or by the patient’s legally acceptable representative”.

11.2.2 Recruitment

It is the responsibility of the investigator to give each patient, prior to inclusion in the trial, full and adequate verbal and written information regarding her rights, the objective and procedures of the trial and the possible risks involved. Confidentiality-related information will be provided. The written patient information must be given to each patient. Patients will be given sufficient time for consideration. An independent

physician will be available in accordance with the requirements of the national law. It is the responsibility of the investigator to obtain signed informed consent from every patient prior to the start of any study related procedure. The written patient information is part of the documentation reviewed by the MEC mentioned. The patient information letter and informed consent form are attached as a separate document.

11.3 Benefits and risks assessment

See paragraph 2.7.

11.4 Compensation for injury

The sponsor/investigator has a liability insurance, which is in accordance with article 7, subsection 6 of the WMO.

The NKI-AVL (sponsor)(also) has an insurance which is in accordance with the legal requirements in the Netherlands (Article 7 WMO and the Measure regarding Compulsory Insurance for Clinical Research in Humans of 23th June 2003). This insurance provides cover for damage to research subjects through injury or death caused by the study.
1. € 450.000,-- (i.e. four hundred and fifty thousand Euro) for death or injury for each subject who participates in the Research;
2. € 3.500.000,-- (i.e. three million five hundred thousand Euro) for death or injury for all subjects who participate in the Research;
3. € 5.000.000,-- (i.e. five million Euro) for the total damage incurred by the organisation for all damage disclosed by scientific research for the Sponsor as 'verrichter' in the meaning of said Act in each year of insurance coverage.

The insurance applies to the damage that becomes apparent during the study or within 4 years after the end of the study.

11.5 Incentives

Not applicable.

12.ADMINISTRATIVE ASPECTS, MONITORING AND PUBLICATION

12.1 Subject identification

Following enrolment a patient sequential identification number or code will be allocated to the patients. This number will be used for identification of the patients and should be reported on all case record forms (CRFs). Data and patient material will be handled confidentially and if possible anonymously. When it is necessary to trace data or material to an individual subject, we will use a subject identification number list to link the data to the subject. The code is based on the sequence of enrolment combined with month-year of the patients' birth date. The key to the code will be safeguarded by the Principle Investigator. The handling of personal data complies with the Dutch Personal Data Protection Act (in Dutch: De Wet Bescherming Persoonsgegevens, WBP).

12.2 Randomization of the patients

There will be no randomization

12.3 Storage and coding of patient material

Patient material will be stored for maximum 15 years in the research laboratories to be used for additional research upon new research developments or insights.

12.3.1 Tissue samples

In total, 3 biopsies will be obtained for tissue sampling:

- 2 biopsies from tumor tissue (2 x 8mm core)
- 1 biopsy from healthy tissue for Luminex assay analysis (1-2mm core).

After biopsies are obtained, they will be immediately transported to the pathology department, where they will be coded and labeled in the Laboratory Management System (LMS) of the NKI-AVL.

For the Luminex assay, tissue must be processed with 'Lysis M' buffer (Roche) as fast as possible (within 4 hours maximum) and stored at -80°C.

See **figure 3** for a schematic overview of tumor and normal tissue biopsies needed for the various assays according to priority, in case not enough tissue can be harvested to perform all assays we wish for.

- N16IMC-1 (wk0)
  - A: Benign tissue, adjacent to the tumor (1-2mm)
    - Fresh Frozen (FF) for Luminex assay
  - B: Tumor (8mm)
    - 4mm for storage and shipping according to Celsius guidelines
    - 4mm FF for later analysis, including cDC1 abundance determination
  - C: Tumor (8mm)
    - 2mm FFPE for routine diagnostics and IHC (by CFMPB)
    - 4mm fresh material directly to B3 for T-cell sorting
- N16IMC-2 (wk5)
  - As described above

12.3.2 Lymph node samples

In case of clinical evident node positive disease at week 5, additional tissue will be harvested from lymph nodes in the surgical specimen. One half of a tumor-positive lymph node will be taken from the neck dissection specimen for research purposes, with the other half undergoing routine pathological investigations. In addition, one half of a clinically tumor-negative lymph node will be harvested. The tumor-negative node is to be sampled as far away from the tumor-positive node as possible.

12.3.3 Blood samples

For PBMC blood collection, 5x10mL natrium heparinized blood bottles are used. Serum for the Luminex assay can be extracted and stored in serum blood bottles. One bottle of 5mL should be sufficient for analysis. Serum tube should stand for 30-45 minutes (max 60 minutes) at room temperature. Next it should be centrifuged 10-15 minutes (depending on rotor 1000-2000 RCF). Serum aliquots must be processed as fast as possible (within 4 hours maximum) and stored at -80°C. 1 mL should be sufficient for analysis. It's imperative that the same blood drawing tube (brand and volume) is used at all times.

12.3.4 Microbiome and metabolome

Storage of samples will be performed at the NKI AVL.
Oral cavity mucosal swabs (total n=20 per patient) will be frozen (-80°C) and stored at AKL. Stool specimen (total: 2 per patient) will be frozen (-80°C) and stored at AKL. After the first 10 and after the second 10 patients, all specimens will be sent to the LUMC for analyses (Department of Medical Microbiology and Chemical Immunology Research team: J.J.C. Neefjes, M.A. Giera, R.D. Zwittink, E.J. Kuijper, A.L. Ciurli, M. Slingerland).

12.3.1 ctDNA in liquid biopsies

Storage of samples will be at the NKI AVL.
The frequency of TP53 mutations in the blood of participants (total n=20) before and after immunotherapy will be determined using a novel technique (Cyclomics), based on Oxford Nanopore MinION sequencing of concatenated copies of a single DNA molecule. After the first 10 and after the second 10 patients, specimens will be sent to the UMCU for analyses (Center for Molecular Medicine, involved researchers W. Kloosterman, J. de Ridder and M. Jager).

12.3.2 Single-cell sequencing

Samples will be collected and processed by the coordinating investigator at the NKI-AVL. They will be stored in the preservation boxes provided by Celsius Inc and shipped for analysis to the USA shortly thereafter.

12.3.3 cDC1 cells in tumor tissue and tumor-draining lymph nodes

Tumor and lymph nodal tissue will be cryopreserved for later analysis of cDCs.

12.4 Data management

All data that are relevant for the study will be collected on CRFs developed by the data centre of the AVL. The completed CRFs must be reviewed, signed and dated by the principal investigator or sub-investigator. The data will be collected and kept at the NKI-AVL. Demographic and disease data will be taken from all patients included in the trial. Variables will be Gender, date of birth, height, weight, WHO performance, physical examination, heart rate, blood pressure, temperature, ECG, result pregnancy test, TNM stage tumor.

12.5 Monitoring and Quality Assurance

Source data verification of the CRFs and check of the Investigator Study File documents will be performed by the clinical research monitor of the NKI-AVL, according to the procedures described in the Monitor Plan.

12.6 Amendments

Amendments are changes made to the research after a favorable opinion by the accredited METC has been given. All amendments will be notified to the METC that gave a favorable opinion.

A 'substantial amendment' is defined as an amendment to the terms of the METC application, or to the protocol or any other supporting documentation, that is likely to affect to a significant degree:

- the safety or physical or mental integrity of the subjects of the trial;
- the scientific value of the trial;
- the conduct or management of the trial; or
- the quality or safety of any intervention used in the trial.

All substantial amendments will be notified to the METC and to the competent authority.

Non-substantial amendments will not be notified to the accredited METC and the competent authority, but will be recorded and filed by the investigator.

12.7 Annual progress report

The sponsor/investigator will submit a summary of the progress of the trial to the accredited METC once a year. Information will be provided on the date of inclusion of the first subject, numbers of subjects included and numbers of subjects that have completed the trial, serious adverse events/ serious adverse reactions, other problems, and amendments.

12.8 End of study report

The investigator will notify the accredited METC and the competent authority of the end of the study within a period of 90 days. The end of the study is defined as the last patient's last visit.

In case the study is ended prematurely, the investigator will notify the accredited METC and the competent authority within 15 days, including the reasons for the premature termination.
Within one year after the end of the study, the investigator will submit a final study report with the results of the study, including any publications/abstracts of the study, to the accredited METC and the Competent Authority.

12.9 Public disclosure and publication policy

Prior to initiation, the study will be submitted to the Dutch National Trial register, which is a recognized and accepted by the World Health Organization and International Committee of Medical Journal Editors (ICMJE) and to the NCI's PDQ® Cancer Clinical Trials Registry. All the results will officially be published.

13. REFERENCES

1. Dutch_Comprehensive_Cancer_Center, *Incidence of head and neck cancer,* http://www.cijfersoverkanker.nl/selecties/HeadNeck/img520e166229228?language=en Accessed August 16th, 2013.

2. Rogers, S.N., et al., *Survival following primary surgery for oral cancer.* Oral Oncol, 2009. **45**(3): p. 201-11.

3. Goodwin, W.J., Jr., *Salvage surgery for patients with recurrent squamous cell carcinoma of the upper aerodigestive tract: when do the ends justify the means?* Laryngoscope, 2000. **110**(3 Pt 2 Suppl 93): p. 1-18.

4. Pignon, J.P., et al., *Chemotherapy added to locoregional treatment for head and neck squamous-cell carcinoma: three meta-analyses of updated individual data. MACH-NC Collaborative Group. Meta-Analysis of Chemotherapy on Head and Neck Cancer.* Lancet, 2000. **355**(9208): p. 949-55.

5. Seiwert, T.Y., *ASCO Expanding the Reach of Anti-PD-1 Therapy.* Cancer Discovery 2015. **July; 5(7): 684-685**.

6. Gillison, *Abstract CT099* AACR meeting, 2016.

7. Larkin, J., et al., *Combined Nivolumab and Ipilimumab or Monotherapy in Untreated Melanoma.* N Engl J Med, 2015. **373**(1): p. 23-34.

8. Rizvi, N.A., *Safety and efficacy of first line Nivolumab and Ipilimumab in Non small Cell Lung Cancer (NSCLC).* Abstract, IASLC, 2015.

9. Blank, C.U., *Feasibility Study to Identify the Optimal Adjuvant Combination Scheme of Ipilimumab and Nivolumab (OpACIN) trial.* Preliminary clinical data, 2016.

10. Toustrup, K., et al., *Gene expression classifier predicts for hypoxic modification of radiotherapy with nimorazole in squamous cell carcinomas of the head and neck.* Radiother Oncol, 2012. **102**(1): p. 122-9.

11. Toustrup, K., et al., *Validation of a 15-gene hypoxia classifier in head and neck cancer for prospective use in clinical trials.* Acta Oncol, 2016. **55**(9-10): p. 1091-1098.

12. Tawk, B., et al., *Comparative analysis of transcriptomics based hypoxia signatures in head- and neck squamous cell carcinoma.* Radiother Oncol, 2016. **118**(2): p. 350-8.

13. Toustrup, K., et al., *Development of a hypoxia gene expression classifier with predictive impact for hypoxic modification of radiotherapy in head and neck cancer.* Cancer Res, 2011. **71**(17): p. 5923-31.

14. Toustrup, K., et al., *Validation of a 15-gene hypoxia classifier in head and neck cancer for prospective use in clinical trials.* Acta Oncol, 2016: p. 1-8.

15. Zitvogel, L., et al., *Cancer and the gut microbiota: an unexpected link.* Sci Transl Med, 2015. **7**(271): p. 271ps1.

16. Garrett, W.S., *Cancer and the microbiota.* Science, 2015. **348**(6230): p. 80-6.

17. Honda, K. and D.R. Littman, *The microbiota in adaptive immune homeostasis and disease.* Nature, 2016. **535**(7610): p. 75-84.

18. Felix, K.M., S. Tahsin, and H.J. Wu, *Host-microbiota interplay in mediating immune disorders.* Ann N Y Acad Sci, 2017.

19. Routy, B., et al., *Gut microbiome influences efficacy of PD-1-based immunotherapy against epithelial tumors.* Science, 2018. **359**(6371): p. 91-97.

20. Gopalakrishnan, V., et al., *Gut microbiome modulates response to anti-PD-1 immunotherapy in melanoma patients.* Science, 2018. **359**(6371): p. 97-103.

21. Banerjee, S., et al., *Microbial Signatures Associated with Oropharyngeal and Oral Squamous Cell Carcinomas.* Sci Rep, 2017. **7**(1): p. 4036.

22. Crowley, E., et al., *Liquid biopsy: monitoring cancer-genetics in the blood.* Nat Rev Clin Oncol, 2013. **10**(8): p. 472-84.

23. Bettegowda, C., et al., *Detection of circulating tumor DNA in early- and late-stage human malignancies.* Sci Transl Med, 2014. **6**(224): p. 224ra24.

24. Tsao, S.C., et al., *Monitoring response to therapy in melanoma by quantifying circulating tumour DNA with droplet digital PCR for BRAF and NRAS mutations.* Sci Rep, 2015. **5**: p. 11198.

25. Newman, A.M., et al., *An ultrasensitive method for quantitating circulating tumor DNA with broad patient coverage.* Nat Med, 2014. **20**(5): p. 548-54.

26. Perdomo, S., et al., *Circulating tumor DNA detection in head and neck cancer: evaluation of two different detection approaches.* Oncotarget, 2017. **8**(42): p. 72621-72632.

27. van Ginkel, J.H., et al., *Targeted sequencing reveals TP53 as a potential diagnostic biomarker in the post-treatment surveillance of head and neck cancer.* Oncotarget, 2016. **7**(38): p. 61575-61586.

28. van Ginkel, J.H., et al., *Droplet digital PCR for detection and quantification of circulating tumor DNA in plasma of head and neck cancer patients.* BMC Cancer, 2017. **17**(1): p. 428.

29. Puram, S.V., et al., *Single-Cell Transcriptomic Analysis of Primary and Metastatic Tumor Ecosystems in Head and Neck Cancer.* Cell, 2017. **171**(7): p. 1611-1624.e24.

30. Jerby-Arnon, L., et al., *A Cancer Cell Program Promotes T Cell Exclusion and Resistance to Checkpoint Blockade.* Cell, 2018. **175**(4): p. 984-997.e24.

31. Sataloff DM, M.B., Prestipino AJ, et al., www.oncoline.nl/mammacarcinoom*, see Eusoma.org. Guideline for defining tumor response to neoadjuvant treatment in mamma carcinoma. Pathologic response to induction chemotherapy in locally advanced carcinoma of the breast: a determinant of outcome.* J Am Coll Surg, 1995;180:297–306.

32. Swartz, J.E., et al., *Poor prognosis in human papillomavirus-positive oropharyngeal squamous cell carcinomas that overexpress hypoxia inducible factor-1alpha.* Head Neck, 2016.

33. Hockel, M. and P. Vaupel, *Tumor hypoxia: definitions and current clinical, biologic, and molecular aspects.* J Natl Cancer Inst, 2001. **93**(4): p. 266-76.

34. Semenza, G.L., *The hypoxic tumor microenvironment: A driving force for breast cancer progression.* Biochim Biophys Acta, 2016. **1863**(3): p. 382-91.

35. Overgaard, J., *Hypoxic modification of radiotherapy in squamous cell carcinoma of the head and neck--a systematic review and meta-analysis.* Radiother Oncol, 2011. **100**(1): p. 22-32.

36. Colbert, L.E., et al., *High nuclear hypoxia-inducible factor 1 alpha expression is a predictor of distant recurrence in patients with resected pancreatic adenocarcinoma.* Int J Radiat Oncol Biol Phys, 2015. **91**(3): p. 631-9.

37. Phan, A.T. and A.W. Goldrath, *Hypoxia-inducible factors regulate T cell metabolism and function.* Mol Immunol, 2015. **68**(2 Pt C): p. 527-35.

38. Denko, N.C., *Hypoxia, HIF1 and glucose metabolism in the solid tumour.* Nat Rev Cancer, 2008. **8**(9): p. 705-13.

39. Swartz, J.E., et al., *Clinical implications of hypoxia biomarker expression in head and neck squamous cell carcinoma: a systematic review.* Cancer Med, 2015. **4**(7): p. 1101-16.

40. Hanns, E., et al., *Human Papillomavirus-related tumours of the oropharynx display a lower tumour hypoxia signature.* Oral Oncol, 2015. **51**(9): p. 848-56.

41. Toustrup, K., et al., *Gene expression classifier predicts for hypoxic modification of radiotherapy with nimorazole in squamous cell carcinomas of the head and neck.* Radiother Oncol, 2012. **102**(1): p. 122-9.

42. Barsoum, I.B., et al., *A mechanism of hypoxia-mediated escape from adaptive immunity in cancer cells.* Cancer Res, 2014. **74**(3): p. 665-74.

43. Labiano, S., A. Palazon, and I. Melero, *Immune response regulation in the tumor microenvironment by hypoxia.* Semin Oncol, 2015. **42**(3): p. 378-86.

44. Palazon, A., et al., *HIF transcription factors, inflammation, and immunity.* Immunity, 2014. **41**(4): p. 518-28.

45. Noman, M.Z., et al., *PD-L1 is a novel direct target of HIF-1alpha, and its blockade under hypoxia enhanced MDSC-mediated T cell activation.* J Exp Med, 2014. **211**(5): p. 781-90.

46. Chang, C.H., et al., *Posttranscriptional control of T cell effector function by aerobic glycolysis.* Cell, 2013. **153**(6): p. 1239-51.

47. Pearce, E.L., et al., *Fueling immunity: insights into metabolism and lymphocyte function.* Science, 2013. **342**(6155): p. 1242454.

48. Cao, Y., J.C. Rathmell, and A.N. Macintyre, *Metabolic reprogramming towards aerobic glycolysis correlates with greater proliferative ability and resistance to metabolic inhibition in CD8 versus CD4 T cells.* PLoS One, 2014. **9**(8): p. e104104.

49. Doedens, A.L., et al., *Hypoxia-inducible factors enhance the effector responses of CD8(+) T cells to persistent antigen.* Nat Immunol, 2013. **14**(11): p. 1173-82.

50. Shehade, H., et al., *Cutting Edge: Hypoxia-Inducible Factor 1 Negatively Regulates Th1 Function.* J Immunol, 2015. **195**(4): p. 1372-6.

51. Shi, L.Z., et al., *HIF1alpha-dependent glycolytic pathway orchestrates a metabolic checkpoint for the differentiation of TH17 and Treg cells.* J Exp Med, 2011. **208**(7): p. 1367-76.

52. Lim, Y., et al., *Oral Microbiome: A New Biomarker Reservoir for Oral and Oropharyngeal Cancers.* Theranostics, 2017. **7**(17): p. 4313-4321.

53. Rygh, C.B., et al., *Dynamic contrast enhanced MRI detects early response to adoptive NK cellular immunotherapy targeting the NG2 proteoglycan in a rat model of glioblastoma.* PLoS One, 2014. **9**(9): p. e108414.

54. *http://www.infernal-colour.eu/Tattoozubehoer/Tattoofarben/Diabolo-Genesis/Diabolo-Genesis-30-ml.*

55. *http://kwadron.com/ig-1/round-liner/0-30mm-long-taper.html.*

56. Schiffmacher, *http://tattooing.nl/.*

57. *http://www.ich.org/fileadmin/Public_Web_Site/ICH_Products/Guidelines/Efficacy/E6_R1/ Step4/E6_R1__Guideline.pdf.*

14. APPENDIX

14.1 Safety algorithms

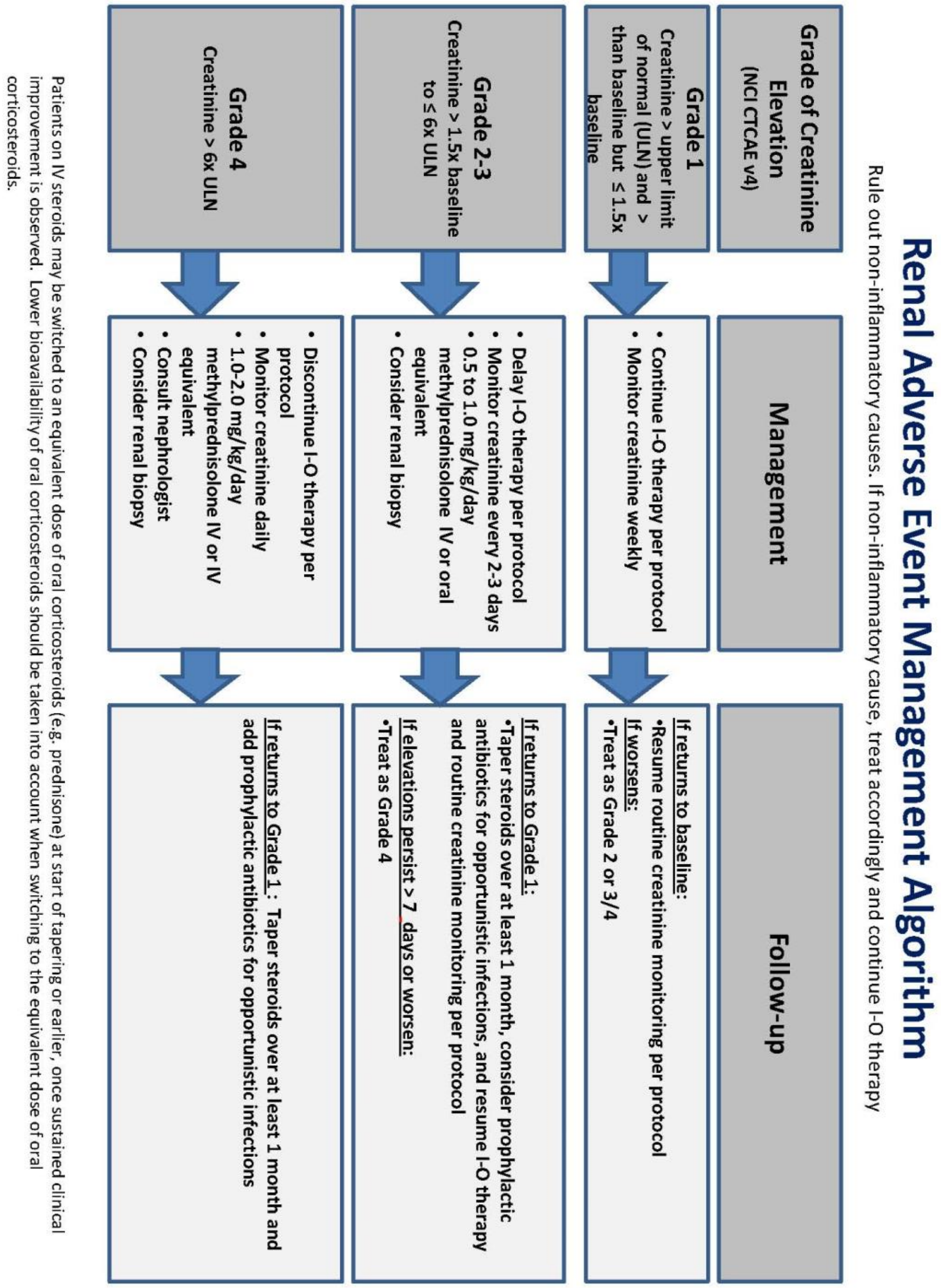

Endocrinopathy Management Algorithm

Rule out non-inflammatory causes. If non-inflammatory cause, treat accordingly and continue I-O therapy. Consider visual field testing, endocrinology consultation, and imaging.

Asymptomatic thyroid stimulating hormone (TSH) elevation →

- Continue I-O therapy per protocol
- If TSH < 0.5 x lower limit of normal (LLN), or TSH > 2 x ULN, or consistently out of range in 2 subsequent measurements: include free thyroxine (fT4) at subsequent cycles as clinically indicated; consider endocrinology consult

Symptomatic endocrinopathy →

- Evaluate endocrine function
- Consider pituitary scan

Symptomatic with abnormal lab/pituitary scan:
- Delay I-O therapy per protocol
- 1-2 mg/kg/day methylprednisolone IV or by mouth (PO) equivalent
- Initiate appropriate hormone therapy

No abnormal lab/pituitary MRI scan but symptoms persist:
- Repeat labs in 1-3 weeks /MRI in 1 month

→

If improves (with or without hormone replacement):
- Taper steroids over at least 1 month and consider prophylactic antibiotics for opportunistic infections
- Resume I-O therapy per protocol
- Patients with adrenal insufficiency may need to continue steroids with mineralocorticoid component

Suspicion of adrenal crisis (e.g. severe dehydration, hypotension, shock out of proportion to current illness →

- Delay or discontinue I-O therapy per protocol
- Rule out sepsis
- Stress dose of IV steroids with mineralocorticoid activity
- IV fluids
- Consult endocrinologist
- If adrenal crisis ruled out, then treat as above for symptomatic endocrinopathy

Patients on IV steroids may be switched to an equivalent dose of oral corticosteroids (e.g. prednisone) at start of tapering or earlier, once sustained clinical improvement is observed. Lower bioavailability of oral corticosteroids should be taken into account when switching to the equivalent dose of oral corticosteroids.

GI Adverse Event Management Algorithm

Rule out non-inflammatory causes. If non-inflammatory cause is identified, treat accordingly and continue I-O therapy. Opiates/narcotics may mask symptoms of perforation. Infliximab should not be used in cases of perforation or sepsis.

Grade of Diarrhea/ Colitis (NCI CTCAE v4)	Management	Follow-up
**Grade 1**<br>Diarrhea:< 4 stools/day over baseline;<br>Colitis: asymptomatic	• Continue I-O therapy per protocol<br>• Symptomatic treatment	• Close monitoring for worsening symptoms.<br>• Educate patient to report worsening immediately<br>If worsens:<br>• Treat as Grade (G) 2 or 3/4
**Grade 2**<br>Diarrhea: 4-6 stools per day over baseline; IV fluids indicated <24 hours (hrs); not interfering with ADL<br>Colitis: abdominal pain; blood in stool	• Delay I-O therapy per protocol<br>• Symptomatic treatment	If improves to grade 1:<br>• Resume I-O therapy per protocol<br>If persists > 5-7 days or recur:<br>• 0.5-1.0 mg/kg/day methylprednisolone or oral equivalent<br>• When symptoms improve to grade 1, taper steroids over at least 1 month, consider prophylactic antibiotics for opportunistic infections, and resume I-O therapy per protocol.<br>If worsens or persists > 3-5 days with oral steroids:<br>• Treat as grade 3/4
**Grade 3-4**<br>Diarrhea (G3): ≥7 stools per day over baseline; incontinence; IV fluids ≥24 hrs; interfering with activities of daily living (ADL)<br>Colitis (G3): severe abdominal pain, medical intervention indicated, peritoneal signs<br>G4: life-threatening, perforation	• Discontinue I-O therapy per protocol<br>• 1.0 to 2.0 mg/kg/day methylprednisolone IV or IV equivalent<br>• Add prophylactic antibiotics for opportunistic infections<br>• Consider lower endoscopy	If improves:<br>• Continue steroids until grade 1, then taper over at least 1 month<br>If persists > 3-5 days, or recurs after improvement:<br>• Add infliximab 5 mg/kg (if no contraindication). Note: Infliximab should not be used in cases of perforation or sepsis

Patients on IV steroids may be switched to an equivalent dose of oral corticosteroids (e.g. prednisone) at start of tapering or earlier, once sustained clinical improvement is observed. Lower bioavailability of oral corticosteroids should be taken into account when switching to the equivalent dose of oral corticosteroids.

Hepatic Adverse Event Management Algorithm

Rule out non-inflammatory causes. If non-inflammatory cause, treat accordingly and continue I-O therapy. Consider imaging for obstruction.

Grade of Liver Test Elevation (NCI CTCAE v4)	Management	Follow-up
**Grade 1**<br>AST or ALT > ULN to 3.0 x ULN and/or Total bilirubin (T. bili) > ULN - 1.5 x ULN	• Continue I-O therapy per protocol	• Continue liver function tests (LFT) monitoring per protocol<br>If worsens:<br>• Treat as Grade 2 or 3-4
**Grade 2**<br>AST or ALT > 3.0 to ≤ 5 x ULN and/or T. bili > 1.5 to ≤ 3 x ULN	• Delay I-O therapy per protocol<br>• Increase frequency of monitoring to every 3 days	If returns to baseline:<br>•Resume routine monitoring, resume I-O therapy per protocol<br>If elevations persist > 5-7 days or worsen :<br>• 0.5-1 mg/kg/day methylprednisolone or oral equivalent and when LFT returns to grade 1 or baseline, taper steroids over at least 1 month, consider prophylactic antibiotics for opportunistic infections, and resume I-O therapy per protocol
**Grade 3-4**<br>AST or ALT > 5 x ULN and /or T.bili >3 x ULN	• Discontinue I-O therapy*<br>• Increase frequency of monitoring to every 1-2 days<br>• 1.0 to 2.0 mg/kg/day methylprednisolone IV or IV equivalent**<br>• Add prophylactic antibiotics for opportunistic infections<br>• Consult gastroenterologist	If returns to grade 2:<br>•Taper steroids over at least 1 month<br>If does not improve in >3-5 days, worsens or rebounds:<br>• Add mycophenolate mofetil 1 gram (g) twice daily (BID)<br>• If no response within an additional 3-5 days, consider other immunosuppressants per local guidelines

Patients on IV steroids may be switched to an equivalent dose of oral corticosteroids (e.g. prednisone) at start of tapering or earlier, once sustained clinical improvement is observed. Lower bioavailability of oral corticosteroids should be taken into account when switching to the equivalent dose of oral corticosteroids.

***I-O therapy may be delayed rather than discontinued if AST/ALT ≤ 8 x ULN and T.bili ≤ 5 x ULN.**

**The recommended starting dose for grade 4 hepatitis is 2 mg/kg/day methylprednisolone IV.

Neurological Adverse Event Management Algorithm

Rule out non-inflammatory causes. If non-inflammatory cause, treat accordingly and continue I-O therapy.

Grade of Neurological Toxicity (NCI CTCAE v4)	Management	Follow-up
**Grade 1**<br>Asymptomatic or mild symptoms; Intervention not indicated	• Continue I-O therapy per protocol	Continue to monitor the patient.<br>If worsens:<br>• Treat as Grade 2 or 3-4
**Grade 2**<br>Moderate symptoms; Limiting instrumental ADL	• Delay I-O therapy per protocol<br>• Treat symptoms per local guidelines<br>• Consider 0.5 to 1.0 mg/kg/day methylprednisolone IV or PO equivalent	If improves to baseline:<br>•Resume I-O therapy per protocol when improved to baseline<br>If worsens:<br>• Treat as Grade 3-4
**Grade 3-4**<br>Severe symptoms; Limiting self-care ADL; Life-threatening	• Discontinue I-O therapy per protocol<br>• Obtain neurology consult<br>• Treat symptoms per local guidelines<br>• 1.0-2.0 mg/kg/day IV methylprednisolone IV or IV equivalent<br>• Add prophylactic antibiotics for opportunistic infections	If improves to Grade 2:<br>• Taper steroids over at least 1 month<br><br>If worsens or atypical presentation:<br>• Consider IVIG or other immunosuppressive therapies per local guidelines

Patients on IV steroids may be switched to an equivalent dose of oral corticosteroids (e.g. prednisone) at start of tapering or earlier, once sustained clinical improvement is observed. Lower bioavailability of oral corticosteroids should be taken into account when switching to the equivalent dose of oral corticosteroids.

Pulmonary Adverse Event Management Algorithm

Rule out non-inflammatory causes. If non-inflammatory cause, treat accordingly and continue I-O therapy. Evaluate with imaging and pulmonary consultation.

Grade of Pneumonitis (NCI CTCAE v4)	Management	Follow-up
**Grade 1**<br>Radiographic changes only	• Consider delay of I-O therapy<br>• Monitor for symptoms every 2-3 days<br>• Consider Pulmonary and Infectious Disease (ID) consults	• Re-image at least every 3 weeks<br>If worsens:<br>• Treat as Grade 2 or 3-4
**Grade 2**<br>Mild to moderate new symptoms	• Delay I-O therapy per protocol<br>• Pulmonary and ID consults<br>• Monitor symptoms daily, consider hospitalization<br>• 1.0 mg/kg/day methyl-prednisolone IV or oral equivalent<br>• Consider bronchoscopy , lung biopsy	• Re-image every 1-3 days<br>If improves:<br>• When symptoms return to near baseline, taper steroids over at least 1 month and then resume I-O therapy per protocol and consider prophylactic antibiotics<br>If not improving after 2 weeks or worsening:<br>• Treat as Grade 3-4
**Grade 3-4**<br>Severe new symptoms; New/worsening hypoxia; Life-threatening	• Discontinue I-O therapy per protocol<br>• Hospitalize<br>• Pulmonary and ID consults<br>• 2-4 mg/kg/day methylprednisolone IV or IV equivalent<br>• Add prophylactic antibiotics for opportunistic infections<br>• Consider bronchoscopy, lung biopsy	If improves to baseline:<br>• Taper steroids over at least 6 weeks<br>If not improving after 48 hours or worsening:<br>• Add additional immunosuppression (e.g. infliximab, cyclophosphamide, intravenous immunoglobulin (IVIG), or mycophenolate mofetil)

Patients on IV steroids may be switched to an equivalent dose of oral corticosteroids (e.g. prednisone) at start of tapering or earlier, once sustained clinical improvement is observed. Lower bioavailability of oral corticosteroids should be taken into account when switching to the equivalent dose of oral corticosteroids.

Skin Adverse Event Management Algorithm

Rule out non-inflammatory causes. If non-inflammatory cause, treat accordingly and continue I-O therapy.

Grade of Rash (NCI CTCAE v4)	Management	Follow-up
**Grade 1-2** Covering ≤ 30% body surface area (BSA)*	• Symptomatic therapy (e.g. antihistamines, topical steroids)<br>• Continue I-O therapy per protocol	<u>If persists > 1-2 weeks or recurs:</u><br>• Consider skin biopsy<br>• Delay I-O therapy per protocol<br>• Consider 0.5-1.0 mg/kg/day methylprednisolone IV or oral equivalent. Once improving , taper steroids over at least 1 month, consider prophylactic antibiotics for opportunistic infections, and resume I-O therapy per protocol<br><u>If worsens:</u><br>• Treat as Grade 3-4
**Grade 3-4** Covering >30% BSA; Life threatening consequences*	• Delay or discontinue I-O therapy per protocol<br>• Consider skin biopsy<br>• Dermatology consult<br>• 1.0-2.0 mg/kg/day IV methylprednisolone IV or IV equivalent	<u>If improves to Grade 1:</u><br>• Taper steroids over at least 1 month and add prophylactic antibiotics for opportunistic infections<br>• Resume I-O therapy per protocol

Patients on IV steroids may be switched to an equivalent dose of oral corticosteroids (e.g. prednisone) at start of tapering or earlier, once sustained clinical improvement is observed. Lower bioavailability of oral corticosteroids should be taken into account when switching to the equivalent dose of oral corticosteroids.

*Refer to NCI CTCAE v4 for term-specific grading criteria.

14.2 ECOG Performance Status Score Definition

0. Fully active, able to carry on all pre-disease activities without restriction
1. Restricted in physically strenuous activity but ambulatory and able to carry out work of a light or sedentary nature, eg, light house work or office work
2. Ambulatory and capable of all selfcare but unable to carry out any work activities. Up and about more than 50% of waking hours
3. Capable of only limited selfcare, confined to bed or chair more than 50% of waking hours
4. Completely disabled. Cannot carry on any selfcare. Totally confined to bed or chair
5. Dead

14.3 Common Terminology Criteria for Adverse Events (CTCAE)

The National Cancer Institute Common Terminology Criteria for Adverse Events (NCI CTCAE), version 4.03, dated 14 June 2010, may be reviewed online at the following NCI website: http://ctep.cancer.gov/reporting/ctc.html

14.4 Response Evaluation Criteria in Solid Tumors (RECIST)

Adapted from E.A. Eisenhauer, et al. New response evaluation criteria in solid tumours: Revised RECIST guideline (version 1.1). European Journal of Cancer 45 (2009) 228–247.

CATEGORIZING LESIONS AT BASELINE

Measurable Lesions

- Lesions that can be accurately measured in at least one dimension.
- Lesions with longest diameter twice the slice thickness and at least 10 mm or greater when assessed by CT or MRI (slice thickness 5-8 mm).
- Lesions with longest diameter at least 20 mm when assessed by Chest X-ray.
- Superficial lesions with longest diameter 10 mm or greater when assessed by caliper.
- Malignant lymph nodes with the short axis 15 mm or greater when assessed by CT.

NOTE: The shortest axis is used as the diameter for malignant lymph nodes, longest axis for all other measurable lesions.

Non-measurable disease

Non-measurable disease includes lesions too small to be considered measurable (including nodes with short axis between 10 and 14.9 mm) and truly non-measurable disease such as pleural or pericardial effusions, ascites, inflammatory breast disease, leptomeningeal disease, lymphangitic involvement of skin or lung, clinical lesions that cannot be accurately measured with calipers, abdominal masses identified by physical exam that are not measurable by reproducible imaging techniques.

- Bone disease: Bone disease is non-measurable with the exception of soft tissue components that can be evaluated by CT or MRI and meet the definition of measurability at baseline.
- Previous local treatment: A previously irradiated lesion (or lesion subjected to other local treatment) is non-measurable unless it has progressed since completion of treatment.

Normal sites

- Cystic lesions: Simple cysts should not be considered as malignant lesions and should not be recorded either as target or non-target disease. Cystic lesions thought to represent cystic metastases can be measurable lesions, if they meet the specific definition above. If non-cystic lesions are also present, these are preferred as target lesions.
- Normal nodes: Nodes with short axis <10 mm are considered normal and should not be recorded or followed either as measurable or non-measurable disease.

RECORDING TUMOR ASSESSMENTS

All sites of disease must be assessed at baseline. Baseline assessments should be done as close as possible prior to study start. For an adequate baseline assessment, all required scans must be done within 28 days prior to treatment and all disease must be documented appropriately. If baseline assessment is inadequate, subsequent statuses generally should be indeterminate.

Target lesions

All measurable lesions up to a maximum of 2 lesions per organ, 5 lesions in total, representative of all involved organs, should be identified as target lesions at baseline.

Target lesions should be selected on the basis of size (longest lesions) and suitability for accurate repeated measurements. Record the longest diameter for each lesion, except in the case of pathological lymph nodes for which the short axis should be recorded. The sum of the diameters (longest for non-nodal lesions, short axis for nodal lesions) for all target lesions at baseline will be the basis for comparison to assessments performed on study.

If two target lesions coalesce the measurement of the coalesced mass is used. If a large target lesion splits, the sum of the parts is used.

Measurements for target lesions that become small should continue to be recorded. If a target lesion becomes too small to measure, 0 mm should be recorded if the lesion is considered to have disappeared; otherwise a default value of 5 mm should be recorded.

NOTE: When nodal lesions decrease to <10 mm (normal), the actual measurement should still be recorded.

Non-target disease

All non-measurable disease is non-target. All measurable lesions not identified as target lesions are also included as non-target disease. Measurements are not required but rather assessments will be expressed as ABSENT, INDETERMINATE, PRESENT/NOT INCREASED, INCREASED. Multiple non-target lesions in one organ may be recorded as a single item on the case report form (eg, 'multiple enlarged pelvic lymph nodes' or 'multiple
liver metastases').

OBJECTIVE RESPONSE STATUS AT EACH EVALUATION

Disease sites must be assessed using the same technique as baseline, including consistent administration of contrast and timing of scanning. If a change needs to be made the case must be discussed with the radiologist to determine if substitution is possible. If not, subsequent objective statuses are indeterminate.

Target disease

- Complete Response (CR): Complete disappearance of all target lesions with the exception of nodal disease. All target nodes must decrease to normal size (short axis <10 mm). All target lesions must be assessed.
- Partial Response (PR): Greater than or equal to 30% decrease under baseline of the sum of diameters of all target measurable lesions. The short diameter is used in the sum for target nodes, while the longest diameter is used in the sum for all other target lesions. All target lesions must be assessed.
- Stable: Does not qualify for CR, PR or Progression. All target lesions must be assessed. Stable can follow PR only in the rare case that the sum increases by less than 20% from the nadir, but enough that a previously documented 30% decrease no longer holds.
- Objective Progression (PD): 20% increase in the sum of diameters of target
- measurable lesions above the smallest sum observed (over baseline if no decrease in the sum is observed during therapy), with a minimum absolute increase of 5 mm.

- Indeterminate. Progression has not been documented and one or more target measurable lesions have not been assessed, or assessment methods used were inconsistent with those used at baseline, or one or more target lesions cannot be measured accurately (eg, poorly visible unless due to being too small to measure), or one or more target lesions were excised or irradiated and have not reappeared or increased.

Non-target disease

- CR: Disappearance of all non-target lesions and normalization of tumor marker levels. All lymph nodes must be ‘normal’ in size (<10 mm short axis).
- Non-CR/Non-PD: Persistence of any non-target lesions and/or tumor marker level above the normal limits.
- PD: Unequivocal progression of pre-existing lesions. Generally the overall tumor burden must increase sufficiently to merit discontinuation of therapy. In the presence of SD or PR in target disease, progression due to unequivocal increase in non-target disease should be rare.
- Indeterminate: Progression has not been determined and one or more non-target sites were not assessed or assessment methods were inconsistent with those used at baseline.

New Lesions

The appearance of any new unequivocal malignant lesion indicates PD. If a new lesion is equivocal, for example due to its small size, continued assessment will clarify the etiology.

If repeat assessments confirm the lesion, then progression should be recorded on the date of the initial assessment. A lesion identified in an area not previously scanned will be considered a new lesion.

Supplemental Investigations

If CR determination depends on a residual lesion that decreased in size but did not disappear completely, it is recommended the residual lesion be investigated with biopsy or fine needle aspirate. If no disease is identified, objective status is CR.

If progression determination depends on a lesion with an increase possibly due to necrosis, the lesion may be investigated with biopsy or fine needle aspirate to clarify

status.

Objective/Subjective Progression

Patients requiring discontinuation of treatment without objective evidence of disease progression should not be reported as PD on tumor assessment CRFs. This should be indicated on the end of treatment CRF as off treatment due to Global Deterioration of Health Status. Every effort should be made to document objective progression even after discontinuation of treatment.

Determination of Best Overall Response

The best overall response is the best response recorded from the start of the treatment until disease progression (taking as reference for progressive disease the smallest sum on study). For CR and PR, the patient’s best response assignment will depend on the achievement of both measurement and confirmation criteria. CR and PR must be confirmed by 2 measurements at least 4 weeks apart. In the case of SD, follow-up measurements must have met the SD criteria at least once after study entry at a minimum interval of 6 weeks.
